# Abstracts from Hydrocephalus 2022: the Fourteenth Meeting of the Hydrocephalus Society

**DOI:** 10.1186/s12987-022-00397-x

**Published:** 2023-01-13

## Analysis of the intracranial pressure waveform in hydrocephalus patients using a non-invasive device

### Raphael Bertani^1^, Caio Perret^2^, Stefan Koester^3^, Paulo Santa Maria^2^, Savio Batista^4^, Tamires Guimarães Cavalcante Carlos de Carvalho^5^, Nicolas Rabelo^1^, Ruy Monteiro^2^

#### ^1^Department of Neurosurgery, University of São Paulo, São Paulo, SP, 01246-000, Brazil; ^2^Department of Neurosurgery, Hospital Municipal Miguel Couto, Rio de Janeiro, RJ, 22430-160, Brazil; ^3^School of Medicine, Vanderbilt University, Nashville, TN, 37240, USA; ^4^Medical school, Universidade Federal do Rio de Janeiro (UFRJ), RJ, 20210-030, Brazil; ^5^Medical school, Universidade Nove de Julho (UNINOVE), São Paulo, SP, 01525-000, Brazil.

##### **Correspondence:** Raphael Bertani (contato@rbertani.com)

*Fluids and Barriers of the CNS* 2022, **19(1)**

**Introduction:** Non-invasive devices that reliably monitor brain compliance can reduce patients’ exposure to potentially harmful or costly imaging modalities. The aim of this study is to assess this situation with a new non-invasive headband device developed by brain4care®.

**Methods:** Patients were submitted to either surgical or valve adjustment procedures and monitored before and after the procedure by the brain4care® headband device. Sixteen symptomatic patients with a previous radiological diagnosis of hydrocephalus, in need of intervention, between the ages of 26 and 73 were analyzed.

**Results:** Out of these patients, 5 were submitted to external ventricular drainages (EVD), 9 to ventriculoperitoneal shunting (VPS), and 2 to valve adjustment. All patients had an abnormal cerebral complacency wave, with P2 > P1 before the procedure, and after, 75% of the patients changed to a normal pattern with P1 > P2. All patients self-reported feeling comfortable with the device.

**Conclusions:** By providing practitioners the Intracranial Pressure (ICP) waveform and values of the P2 / P1 ratio, without quantification of ICP values, this non-invasive device can decrease costs, the time needed to diagnose whether changes or revision of the shunt, and complications of invasive methods for ICP monitoring. Moreover, it can be used in scenarios where invasive ICP monitoring is not indicated, yet would still be insightful.

## Novelties in understanding the pathophysiological functioning of CSF biomarkers in NPH: literature review

### Adéla Bubeníková^1,2^, Petr Skalický^1,2^, Ondřej Bradáč^1,2^

#### ^1^Department of Neurosurgery and Neurooncology, Charles University, Military University Hospital, Prague, Czech Republic; ^2^Department of Neurosurgery, Charles University, Motol University Hospital, Prague, Czech Republic

##### **Correspondence:** Ondřej Bradáč (ondrej.bradac@uvn.cz)

*Fluids and Barriers of the CNS* 2022, **19(1)**

**Introduction:** Considering the high prevalence of comorbidities in normal pressure hydrocephalus (NPH) patients, there is a demand for more precise diagnostic measures of the disease. In relation to its pathogenesis, an investigation of cerebrospinal fluid (CSF) biomarkers could be potentially applicable in clinical practice and might contribute to a more definite diagnosis of NPH.

**Methods:** A literature review of published series dedicated to the topic was performed. The aim was to identify any potential NPH-specific CSF biomarkers, whether there are any significant differences between NPH and non-NPH, healthy controls and other comorbidities and to determine how CSF biomarkers mirror so far known pathophysiological mechanisms in NPH. Additionally,, based on these findings, future implications were introduced.

**Results:** Neurosteroids, particularly dehydroepiandrosterone and its derivatives, are promising candidates for clinical use in NPH diagnosis. Although the concentrations of inflammatory biomarkers, tau- and amyloid-β proteins seem to be well differentiated from healthy controls, the distinction among other neurodegenerative disorders based only on these parameters remains a challenge. Combinations of mentioned biomarkers typically show a better diagnostic accuracy in distinguishing between NPH and movement/cognitive disorders than a single biomarker alone.

**Conclusions:** This research could not unequivocally determine any NPH-specific biomarker. A combination of biochemical analysis, clinical and radiological parameters evaluated through advanced mathematical methods may be a key to precise diagnosis of NPH and exact identification of concurrent comorbidities. Prediction of NPH progression, based on these variables, may be of great importance prospectively to deliver better treatment outcomes and improved prognosis in NPH patients.

## The benefits of automated CSF drainage in normal pressure hydrocephalus

### Sogha Khawari, Maria Kneizeh, Mohamed Elborady, Lewis Thorne, Ahmed Toma, Laurence Watkins

#### ^1^Victor Horsley Department of Neurosurgery, National Hospital for Neurology and Neurosurgery, London, UK.

##### **Correspondence:** Sogha Khawari (sogha.khawari@nhs.net)

*Fluids and Barriers of the CNS* 2022, **19(1)**

**Introduction:** The most commonly-used CSF drainage system remains the manual drip-chamber drain. The LiquoGuard (Möller Medical GmbH, Germany) is an automated CSF management device with dual functionality, measuring ICP and automatic pressure- or volume-led CSF drainage. There is limited research for comparison of devices, particularly in the neurosurgical field, where it has potential to reshape care. Our aim is to compare manual drip-chamber drain versus LiquoGuard system, by assessing accuracy of drainage, associated morbidity and impact on length of stay.

**Methods:** Inclusion criteria consisted of suspected NPH patients undergoing extended lumbar drainage. Patients were randomised into manual drain group versus automated group.

**Results:** Data was analysed from 42 patients; 31 in the manual group versus 11 in the LiquoGuard group. Volumetric over-drainage was seen in 90.3% versus 0% (p < 0.05), and under-drainage in 38.7% versus 0% (p < 0.05), in the manual and automatic group respectively. Symptoms of over-drainage were noted in 54.8% of the manual group, all of which had episodes of volumetric over-drainage, versus 18.2% in automated group, of which neither had actual over-drainage (p < 0.05). Higher over-drainage symptoms of manual drain is likely due to increased fluctuation of CSF drainage, instead of smooth CSF drainage seen with LiquoGuard system. An increased length of stay was seen in 38.7% versus 9% (p < 0.05), in the manual and LiquoGuard group respectively.

**Conclusions:** The LiquoGuard device is a more superior way of CSF drainage in suspected NPH patients, with reduced morbidity and length of stay.

## Resistance to cerebrospinal fluid outflow versus “pseudo” intracranial compliances in hydrocephalus patients

### Cyrille Capel^1,2^, Kimi Owashi^1^, Vakaramoko Kone^1^, Serge Metanbou^1,3^, Roger Bouzerar^4^, Zofia Czosnyka^5^, Marek Czosnyka^5^, Peter Smielewski^5^, Olivier Balédent^1,4^

#### ^1^Chimère UR 7516, Jules Verne University, Amiens, France; ^2^Neurosurgery, Jules Verne University hospital, Amiens, France; ^3^Radiology, Jules Verne University hospital, Amiens, France; ^4^Image processing, Jules Verne University hospital, Amiens, France; ^5^Department of Clinical Neurosciences, University of Cambridge, Cambridge, UK

##### **Correspondence:** Olivier Balédent (olivier.baledent@chu-amiens.fr)

*Fluids and Barriers of the CNS* 2022, **19(1)**

**Introduction:** The resistance to cerebrospinal fluid (CSF) outflow (**R**_**out**_) has been considered as a complementary parameter to predict shunt responsiveness in patients with normal pressure hydrocephalus (NPH). Additionally, a “pseudo” intracranial compliance (**C**_**infus**_) can be defined by considering the change in intracranial pressure (ICP) in response to a known volume change injected during infusion test. We propose to also evaluate a physiological brain compliance (**C**_**physio**_) related to instantaneous intracranial volume (blood and CSF) change and ICP amplitude during the cardiac cycle. We aim to evaluate the relationship between these compliances and **R**_**out**_.

**Methods:** 26 patients (74 ± 9 years) with suspected NPH underwent infusion test and PC-MRI. **C**_**infus**_ was calculated by dividing the volume injected by the ICP change during infusion**.** From PC-MRI, the intracranial liquid volume change (blood and CSF) during the cardiac cycle (CC) was quantified. The ICP change in amplitude at rest over the CC was assessed from ICP monitoring before infusion. Changes in volume and pressure along the CC were used to calculate **C**_**physio**_**.** After infusion, **R**_**out**_ was estimated by ICM + .

**Results:** Correlations between compliances and **R**_**out**_ were respectively R^2^ = 0.24 (p < 0.05) and R^2^ = 0.012 (p < 0.05) for **C**_**infus**_ and **C**_**physio**_**.**

**Conclusions:** These preliminary results show that **C**_**infus**_ and **R**_**out**_ tend to have a higher correlation than **C**_**physio**_ and **R**_**out**_. Indeed, **C**_**infus**_ reflects the brain capacity to respond to continuous volume injection over prolonged time, which could be associated mainly with **R**_**out**_ and venous compliance. Contrary, **C**_**physio**_ reflects instantaneous physiological compliance of intracranial volume directly related to brain biomechanics.

**Study supported by**: Revert Project, Interreg, France (Channel Manche) England, funded by European Regional Development Fund.

## Three types of post-traumatic hydrocephalus

### M. Marek Czosnyka^1^, Laurent Gergele^2^, A. Afroditi Lalou^1^, Alexis J. Joannides^1^, Peter Smielewski^1^, Zofia Czosnyka^1^

#### ^1^Division of Neurosurgery, Department of Clinical Neurosciences, Cambridge University, Cambridge, CB2 0QQ, UK; ^2^Department of Intensive Care, Ramsay Santé, Hôpital privé de la Loire, Saint Etienne, France

##### **Correspondence:** Marek Czosnyka (mc141@medschl.cam.ac.uk)

*Fluids and Barriers of the CNS* 2022, **19(1)**

**Introduction:** There are three forms of post-traumatic hydrocephalus (PtH): 1. External hydrocephalus (EH) or early acute ventriculomegaly. 2. Gradual ventricular dilatation developing after craniectomy. 3. Late non-acute PtH.

Our objective was to characterize their CSF dynamics.

**Method:** 102 patients with traumatic brain injury (TBI) were examined to detect radiological picture of external hydrocephalus. Findings were compared to bedside monitoring data. In patients with craniectomy, CSF dynamics were compared before and after cranioplasty (N = 3). In patients with late non-acute PtH, computerised infusion tests were performed. Those patients (with dilated ventricles, atrophic changes, diminished GCS, and without open craniectomy; N = 33) were compared to patients classified as typical idiopathic normal pressure hydrocephalus (iNPH).

**Results:** In EH, initial ICP was lower than after radiological signs of EH were found (p < 0.05). Patients with EH had a worse outcome and more frequently needed a shunt later (p < 0.05). The tSAH is the most important risk factor to develop EH after TBI. CSF circulation is not disturbed in all cases of ventricular dilatation after craniectomy and not all patients require shunting. CSF dynamics should be assessed after cranioplasty. In both late PtH and NPH baseline ICP was normal and almost the same (cumulative 9.7 ± 4.8 mmHg). Pulse amplitude was significantly greater in iNPH than in PtH (4.5 ± 3.1 mmHg; 2.28 ± 2.9 mmHg, p = 0.0001). Resistance to CSF outflow was higher in iNPH (17.6 ± 5.6 mmHg/(ml/min) than in PtH (13.4 ± 6.1 mmHg/(ml/min)).

**Conclusion:** Different types of hydrocephalus may develop after TBI at different timepoints, each requiring bespoke management. External Hydrocephalus- requires CSF drainage. Ventricular dilatation after craniectomy- cranioplasty and if CSF dynamics remains disturbed- shunting. Late PtH- shunting when CSF dynamics is clearly disturbed.

**Study supported by**: Revert Project, Interreg, France (Channel Manche) England, funded by ERDF.

## Sleep stages variation in intracranial pressure and pulse amplitude

### Lucia Darie^1^, Matthew Bancroft^1^, Dolora Glorioso^2^, Anand Pandit^1^, Eleanor Moncur^1^, Lewis Thorne^1^, Jeremy Radcliffe^3^, Sofia Eriksson^2^, Laurence Watkins^1^ and Ahmed Toma^1^

#### ^1^Victor Horsley Department of Neurosurgery, National Hospital for Neurology and Neurosurgery, University College London Hospitals, London, UK; ^2^Department of Clinical and Experimental Epilepsy, National Hospital for Neurology and Neurosurgery, University College London Hospitals, London, UK; ^3^Department of Neuroanaesthesia and Intensive Care, National Hospital for Neurology and Neurosurgery, University College London Hospitals, London, UK.

##### **Correspondence:** Lucia Darie (lucia.darie@nhs.net), Ahmed Toma (ahmedtoma@nhs.net)

*Fluids and Barriers of the CNS* 2022, **19(1)**

**Keywords:** Sleep stages, Polysomnography, Intracranial pressure, Pulse amplitude

**Introduction:** It is thought that CSF pulsatility has an important role in brain physiology during sleep. Yet few studies have looked into changes of ICP and pulsatility during sleep. This study’s objective was to elucidate whether there is sleep stage related variability in intracranial pressure and pulse amplitude.

**Method:** A single centre prospective cohort study. Patients undergoing 24 h ICP monitoring for suspected CSF dynamic disorders underwent concomitant polysomnography. Clinical and radiological presentation were derived from the patient’s records. Aggregate mean ICP and pulse amplitude (mPA) values were recorded for each sleep stage. Sleep staging was performed in conformity with the AASM guidelines. Within subject values were compared using repeated-measures, one-way ANOVA. Post-hoc paired t-tests were used to compare sleep stages between groups corrected for multiple comparisons (Benjamini–Hochberg method).

**Results:** A total of 12 patients (10 females, 2 males; mean age 40.8 years, SD ± 12.9) with complete data were analysed. There were significant differences in mean ICP between sleep stages (F(3,33) = 10.7, p < 0.00001). Post-hoc paired t-tests revealed significant differences between rapid eye movement (REM; mean ICP 14.4 mmHg) and all other sleep stages (mean ICP/N1 = 12.0 mmHg, t = − 3.6, p = 0.004; ICP/N2 = 11.8 mmHg, t = − 4.3, p = 0.001; ICP/N3 = 12.3 mmHg, t = − 3.5, p = 0.004)**.** Significant differences were also found in mPA between sleep stages (F(3,33) = 5.7, p = 0.003) confirmed on post-hoc paired t-tests: REM (mean mPA 5.5 mmHg and all other sleep stages: mPA/N1 = 4.7 mmHg, t = -2.3, p = 0.04; mPA/N2 = 4.3 mmHg, t =− 2.7, p = 0.02; mPA/N3 = 4.5 mmHg, t = − 2.3, p = 0.04).

**Conclusion:** REM is characterized by higher ICP and mPA values, result confirmed in near-normal patients included in this cohort.

**Funding:** The authors received funding from The National Brain Appeal and Institute of Neurology, University College London

**Compliance with ethical standards:** Ethical approval for this study was obtained in conformity to the University College London standards.
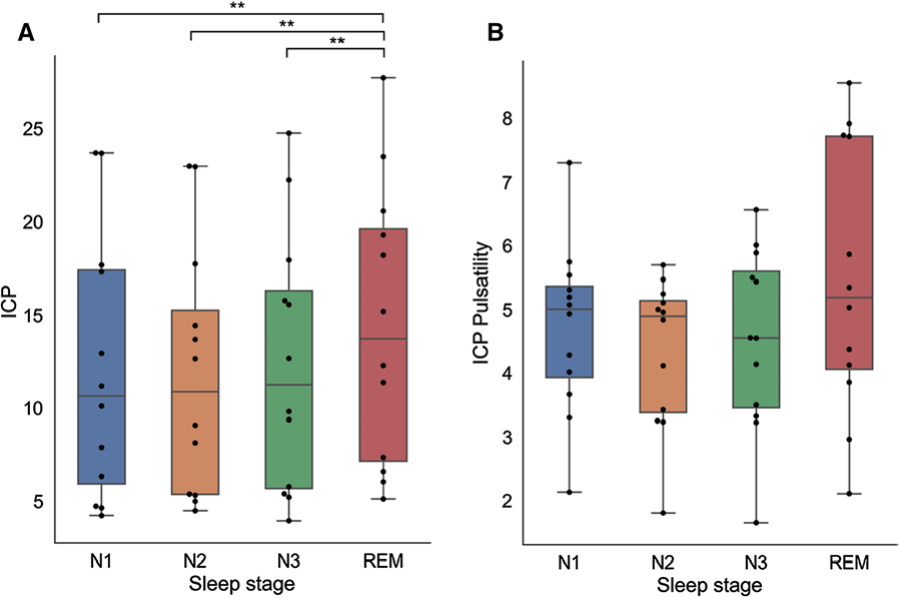


**Figure 1.** Comparison of within-subject mean ICP values **A** and mPA (ICP pulsatility) **B** across sleep stages. (* = p < 0.05, ** = p < 0.01, straight line = multiple comparison corrected).

## “Pseudo” intracranial compliances in hydrocephalus patients

### Olivier Balédent^1,2^, Kimi Owashi^1^, Vakaramoko Kone^1^, Serge Metanbou^1,3^, Roger Bouzerar^2^, Zofia Czosnyka^4^, Marek Czosnyka^4^, Peter Smielewski^4^, Cyrille Capel^1,4^

#### ^1^Chimère UR 7516, Jules Verne University, Amiens, France; ^2^Image processing, Jules Verne University hospital, Amiens, France; ^3^Radiology, Jules Verne University hospital, Amiens, France; ^4^Department of Clinical Neurosciences, University of Cambridge, Cambridge, UK; ^5^Neurosurgery, Jules Verne University hospital, Amiens, France

##### **Correspondence:** Olivier Balédent (olivier.baledent@chu-amiens.fr)

*Fluids and Barriers of the CNS* 2022, **19(1)**

**Introduction:** A **“**Pseudo” Intracranial compliance (**C_infus**) as compensatory mechanism can be simply calculated by combining the increase in intracranial pressure (ICP) with the large volume of liquid slowly injected during infusion test. As Contrary to Marmarou’s doctrine, during cardiac cycle, cerebral blood and cerebrospinal fluid (CSF) flows lead to small intracranial volume change that acts as rapid physiological infusion test, resulting in ICP increase related to physiological brain compliance (**C_physio**). PC-MRI can measure CSF and blood flows during cardiac cycle. Aim of this work, is to observe whether **C_physio** could be complementary to **C_infus**.

**Methods:** 15 patients (71 ± 9 years) with suspected normal pressure hydrocephalus underwent PC-MRI and infusion test within few days. **C_infus** was calculated by dividing the volume injected by the ICP change during infusion**.** Amplitude of ICP change during cardiac cycle (P_ICM+_) was calculated before the start of injection. Intracranial arteries and veins flows and cervical CSF oscillations were measured by PC-MRI to calculate intracranial liquid volume change during cardiac cycle (V_PCMRI_), origin of physiological ICP pulsatility. Then we calculated **C_physio** = V_PCMRI_ /P_ICM+_.

**Results: C_infus** (0.720 ± 0.39 mL/mmHg) was significantly higher than **C_physio** (0.115 ± 0.09 mL/mmHg) with poor correlation (R^2^ = 0.15, p > 0.05).

**Conclusions:** These preliminary results show that the two compliances are different, uncorrelated and complementary. **C_infus** reflects the brain capacity to respond to continuous volume injection over prolonged time, it could be associated mainly with resistance to CSF outflow and venous compliance. **C_physio** reflects instantaneous physiological compliance of intracranial volume directly related to brain biomechanics.

**Study supported by**: Revert Project, Interreg, France (Channel Manche) England, funded by European Regional Development Fund.

## Intracranial pressure dynamics during gait

### Matthew J Bancroft^1,2,3^, Eleanor M. Moncur^1,3^, Lewis Thorne^1,3^, Lawrence D. Watkins^1,3^, Ahmed K. Toma^1,3^

#### ^1^Department of Brain Repair and Rehabilitation, UCL Queen Square Institute of Neurology, London, WC1N 3BG, UK; ^2^Department of Clinical and Movement Neurosciences, UCL Queen Square Institute of Neurology, London, WC1N 3BG, UK; ^3^Victor Horsley Department of Neurosurgery, National Hospital for Neurology and Neurosurgery, Queen Square, London, WC1N 3BG, UK

##### **Correspondence:** Matthew J. Bancroft (matthew.bancroft.13@ucl.ac.uk)

*Fluids and Barriers of the CNS* 2022, **19(1)**

**Introduction:** We investigate intracranial pressure (ICP) dynamics during gait in a mixed-pathology ambulatory cohort.

**Methods:** Patients (n = 17) stood upright and still for 2 min, then walked back and forth along the hospital ward at a self-selected pace for 2 min, followed by another 2 min of standing. ICP was measured by intraparenchymal probe (Raumedic Neurovent-P). Gait phases were extracted from inertial measurement units (IMUs) attached to the head, chest, pelvis and bilateral feet, upper and lower legs. ICP and IMU data were both recorded at 100 Hz and synchronised precisely.

**Results:** The classically-shaped ICP waveform observed during standing was immediately abolished upon the initiation of gait and replaced with a more variable, pseudo-classic waveform characterised by greater mean ICP, and pulsations with larger amplitude and at higher frequency compared to standing (1.8–4.0 mmHg, 3.5–5.6 mmHg and 0.04–0.18 Hz increase, respectively [95% CI]; all p < 0.01). ICP also varied within the gait cycle and systematically decreased around heel strike. This modulation was not easily explainable by mechanical effects, nor head or torso motion. Rather, the timing of heel strike and diastolic ICP tended to be synchronised (phase = -18 [i.e. ~ 0] deg, r = 0.4, p = 0.07) potentially due to cardio-locomotor coupling. ICP immediately returned to the classically-shaped waveform upon termination of gait but ICP remained elevated, with pulsations at larger amplitude and higher frequency than the previous standing period for a variable time (19–59 s [95%CI]) before returning to normal.

**Conclusions:** We demonstrate that ICP is modulated by gait, both on average when compared to standing and on a step-by-step basis.

## Normal pressure hydrocephalus: frequent comorbidities, incidence of post operative seizures, abdominal pain and quality of life

### Lucia Darie^1^, Alaa Al-Mohammad^1^, Anse Arif^2^, Mahi Manoharan^2^, Monty Gwynne^2^, Lewis Thorne^1^, Laurence Watkins^1^ and Ahmed Toma^1^

#### ^1^Department of Neurosurgery, National Hospital for Neurology and Neurosurgery, University College London Hospitals, London, UK; ^2^Medical student at University College London Medical School, London, UK

##### **Correspondence:** Lucia Darie (lucia.darie@nhs.net), Ahmed Toma (ahmedtoma@nhs.net)

*Fluids and Barriers of the CNS* 2022, **19(1)**

**Keywords:** Normal pressure hydrocephalus, Post operative seizures, Abdominal pain, Comorbidities, Quality of life

**Introduction:** The objective of this study was to determine the frequency of post operative seizures and abdominal pain in idiopathic normal pressure hydrocephalus (iNPH) patients who underwent shunt insertion as well as their comorbidities and overall quality of life.

**Methods:** This is a single centre retrospective case series study that included patients treated with a lumboperitoneal (LPS) or ventriculoperitoneal shunt (VPS) for iNPH from 2009 to 2021. Exclusion criteria were patients lost to follow up, refused participation or deceased. Demographic, clinical and radiological data were derived from the records. Postoperative seizures, abdominal pain and overall quality of life were assessed via telephone questionnaires.

**Results:** Data from 135 patients (133 males, 32 females) with a mean age of 74 (SD ± 7.55) years was analysed. 134 patients received a VPS, with all proximal catheters placed parietally. Out of the 3 patients with an initial LPS, two underwent VPS insertion at a later point. None had experienced new onset of epileptic seizures. Post operative abdominal pain was reported in 29 cases (21.6%) and estimated at a mean of VAS 6/10 (SD ± 2). Cardiovascular diseases were commonly encountered in the past medical history. Patients reported an overall health of 60/100 (SD ± 24).

**Conclusion:** Epilepsy is not a common post operative complication following shunt insertion. This could be a relevant finding in particular to patients living in countries where temporary legal driving restrictions have a negative impact on their already impaired mobility.

**Funding and disclosures:** The authors did not receive any funding for the completion of this work. The authors report no conflicts of interest

## Longstanding overt ventriculomegaly in adults (LOVA): open versus closed aqueduct subgroup characteristics

### Lucia Darie^1^, Sogha Khawari^1^, Siegfried Sulim Adelhoefer^3^, Xinrui Ma^2^, Ishrat Hussain^2^, Mary Thomas^2^, Lewis Thorne^1^, Ahmed Toma^1^ and Laurence Watkins^1^

#### 1Victor Horsley Department of Neurosurgery, National Hospital for Neurology and Neurosurgery, University College London Hospitals, London, UK; 2Medical student at University College London Medical School, London, UK; 3Medical student at Universtitätsmedizin Berlin Charité, Germany

##### **Correspondence:** Lucia Darie (lucia.darie@nhs.net), Laurence Watkins (laurence.watkins1@nhs.net)

*Fluids and Barriers of the CNS* 2022, **19(1)**

**Keywords**: Hydrocephalus, Longstanding overt ventriculomegaly in adults, Aqueduct patency, MRI CISS, CSF flow studies, Intra cranial pressure monitoring

**Introduction:** Two subtypes of LOVA can be distinguished dependent on aqueduct patency, both scarcely described in the literature with regards to clinical and radiological presentation.

**Methods:** This is a single centre retrospective case series study. Exclusion criteria: aqueduct stenosis caused by tectum or tegmentum lesions. Demographic, clinical and radiological data were derived from the patient’s records. Where available, 24 h intracranial pressure monitoring (ICPm) results were considered.

**Results:** 82 patients between the age of 16 and 61 years (mean 44.5; SD ± 15.9) were included, of which 76 had complete images and were dichotomized according to the patency of the aqueduct in: open (37) versus closed (39). There was no significant gender distribution (43 women, 33 males). Mean age at presentation was 48.8 years (SD ± 15.3) in the open aqueduct group and 39.5 (SD ± 15.7) in the latter. Both groups have similar incidental presentation rate. No significant difference in the degree of ventriculomegaly (mean FOHR 0.5, SD ± 0.63 versus 0.49, SD ± 0.79) was found. The main radiological findings in patients with patent CSF pathways was a funnel-shaped form of the aqueduct, small cerebellum, enlargement of cisterna magna and panventriculomegaly. Despite being considered to be a communicating type of hydrocephalus, inferior depression of the third ventricular floor was noted in 36/37 (97.2%) of the cases. MRI CSF flow studies and CISS sequences showed no flow obstruction at the cranio-cervical junction. ICPm recordings showed an increase in pulse amplitude.

**Conclusion:** Distinct characteristics are found in these two LOVA subgroups.

**Funding and disclosures**: The authors did not receive any funding for the completion of this work. The authors report no conflicts of interest

## CSF dynamics in Pseudotumour Cerebri Syndrome: a single-centre observational study

### Afroditi Lalou^1^, Zofia Czosnyka^1^, Marek Czosnyka^1^

#### Division of Neurosurgery, Department of Clinical Neurosciences, University of Cambridge, United Kingdom

##### **Correspondence:** Afroditi Lalou (adl43@cam.ac.uk)

*Fluids and Barriers of the CNS* 2022, **19(1)**

**Introduction:** Overnight ICP monitoring, infusion tests and cerebral haemodynamics can reveal insights into the pathophysiology of IIH. In this study, we aimed to identify & describe in vivo studies that demonstrate significant variability in pressure of IIH patients.

**Methods:** We searched for the CSF infusion tests and overnight ICP monitorings performed on patients with suspected or confirmed IIH between January 2003-December 2020. We excluded all patients with a shunt in situ and selected recordings that represented unstable patterns of ICP changes in PTCS.

**Results**: During this timeframe, we had performed 463 of CSF infusion tests and 26 ICP monitorings of PTCS patients. We identified cases with unstable ICP due to various mechanisms, including significant activity of vasogenic wave, collapsing venous sinuses and even unstable ICP due to waves > 50 mmHg. Increased elasticity or depleted compensatory reserve were almost always present in adult patients with diagnosed IIH, regardless of the opening pressure..

**Conclusions:** ICP in IIH can be elevated in different ways. As we have shown from our recordings, ICP could segmentally appear misleadingly “normal” (even < 15 mmHg). Vasogenic waves and influences from the opening and collapsing of the venous sinuses can be amongst factors that cause significant ICP fluctuations. “Normal” versus pathological should be focused on revealing a pathological process that correlates with the clinical syndrome, rather than chasing an arbitrary, and unreliable, number. A comprehensive investigation of parameters and mechanisms involved in IIH is required in order to understand & treat the condition.

## A rare case report including hydatid cyst of the Aqueduct of Sylvius and a Chiari malformation

### Gallaoui Slim^1^, Ben Fredj Rihab^1^, Hattab Omar^1^, Elmir Asma^1^, Laafif Sinda^1^, Kharrat Mohamed Ali^1^, Chabaane Mohamed^1^, Saadaoui Khalil^1^, Ksira Iadh^1^, Maamri Kais^2^

#### ^1^Department of Neurosurgery, Sahloul university hospital, Sousse, Tunisia; ^2^Department of Neurosurgery, Fattouma Bourguiba University Hospital, Monastir, Tunisia

##### **Correspondence:** Gallaoui Slim (slimgallaoui@gmail.com)

*Fluids and Barriers of the CNS* 2022, **19(1)**

**Introduction:** Intracranial hydatid cysts would represent 2–3% of all Echinococcus granulosus infestations. They are most commonly seen in the supra-tentorial territory of the middle cerebral artery. The intraventricular site, especially the aqueduct of Sylvius is exceptional.

**Methods:** We present a very rare case of a primary isolated hydatid cyst of the aqueduct of Sylvius in a 3-year-old girl who was admitted for vomiting, progressive drowsiness and headaches.

**Results:** The preoperative diagnosis made by MRI revealed an acute triventricular hydrocephalus and a cerebellar ptosis mainly attributed to increased intracranial pressure as the child had recovered after a total cyst removal by a posterior approach and the post-operative MRI confirmed the decrease of the enlarged ventricles. However, we were surprised by her rehospitalization for the same symptoms after 4 months and then, the MRI demonstrated a quadriventricular hydrocephalus with no cyst inside but a Chiari I malformation. She underwent successfully a Ventriculoperitoneal shunt with over a 7-year follow up.

**Conclusions:** To our knowledge, this is the first reported case of hydatid cyst of the aqueduct of sylvius which has delayed the reveal of a Chiari malformation. Consent to publish had been obtained.

## New sleep apnea diagnosis following elective intracerebral pressure monitoring

### Aruna Rao^1^, Michael Meggyesy^1^, Gwendolyn Williams^1^, Enoch Kim^2^, Richard Um^1^, Dipankar Biswas^1^, Abhay Moghekar^1^, Mark G Luciano^1^

#### ^1^Department of Neurosurgery, Johns Hopkins University School of Medicine, Baltimore, MD, 21205, USA; ^2^Nova Southeastern University Dr. Kiran C. Patel College of Allopathic Medicine, Fort Lauderdale, FL, 33314, USA

##### **Correspondence:** Aruna Rao (arao10@jhmi.edu)

*Fluids and Barriers of the CNS* 2022, **19(1)**

**Introduction:** Sleep apnea (SA) has been shown to affect intracerebral pressures. Continuous overnight Intracerebral pressure (ICP) monitoring with oximetry is a useful method for objective evaluation in cerebrospinal fluid (CSF) related disorders where subjective symptoms are ambiguous and helps guide treatment.

**Methods:** Retrospective analysis of overnight continuous ICP data was collected from 274 patients admitted from June 2016 till June 2021. Analog CSF pressure transduced via Codman intraparenchymal probe was saved digitally. Oxygen saturation was measured using pulse oximeter. Respirations were measured with impedance plethysmography from ECG electrodes. CSF pressure and oxygen saturations were calculated during consecutive 60 s intervals. ICP above 20 mm-Hg for 15 min is considered CSF hypertension. SA is defined by observed apneic events and a 4% decrease in oxygen saturation for at least 10 s. Polysomnograms were reviewed when available.

**Results:** Of a total 274 patients, 47 (17.1%) were found to have SA. Of these, 30 (63.8%) patients had normal ICP, and 15 (31.9%) CSF hypertension. SA and CSF hypertension was statistically found to not be mutually independent using chi square test (alpha = 0.10), p = 0.067. Of 10 patients with polysomnogram confirmed SA, 7 (70%) had predominantly rapid eye movement sleep (REM) related apnea. Noninvasive ventilation demonstrated decrease in ICP in 7 patients.

**Conclusions:** ICP monitoring with oximetry was useful in accurately identifying SA patients with normal ICP from those with ICP related pathology, suggesting SA can mimic CSF related pathology in presentation. Treatment of SA decreased ICP. REM related apnea was predominant.

## Non-invasive acquisition of head dielectric properties during tilting

### Andrea Boraschi^1^, Andreas Spiegelberg^1^, Vartan Kurtcuoglu^1,2,3^

#### ^1^The Interface Group, Institute of Physiology, University of Zurich, Switzerland; ^2^Zurich Center for Integrative Human Physiology, University of Zurich, Switzerland; ^3^Neuroscience Center Zurich, University of Zurich, Switzerland

##### **Correspondence:** Andrea Boraschi (andrea.boraschi@uzh.ch)

*Fluids and Barriers of the CNS* 2022, **19(1)**

**Introduction:** The assessment of craniospinal compliance (CC) is relevant for the diagnosis of idiopathic normal pressure hydrocephalus. To that end, invasive volume loading methods are required. Recently, a novel apparatus measuring non-invasively variations of head dielectric properties during cardiac and respiratory cycles has been proposed as the derivation of CC surrogates. Here, we investigate the effect of tilting on the electrical signal (referred to as W in the following) acquired with this apparatus.

**Methods:** Nineteen young (age: 25 ± 2 years) healthy subjects participated in this study. To measure W, two isolated electrodes were placed on the subjects’ forehead, in areas corresponding to the F3, F7 and F4, F8 electrodes in a 10–20 electroencephalogram setup. The protocol consisted of 10 min in supine horizontal position followed by 5 min at the tilt angles + 75°, 0° (control) and -30°. The peak-to-valley amplitude of W related to cardiac modulation (AMP) was computed.

**Results:** In all volunteers, characteristic cardiac and respiratory oscillations were observed in W. AMP decreased during head-up tilting (0°: 100%; + 75°: 80 ± 17%, P = 0.002). After returning to its resting state value during the control period (0° control: 95 ± 11%, P = 0.12), AMP increased during head-down tilting (-30°: 148 ± 36%, P < 0.001).

**Conclusions:** Tilting alters the distribution of CC between cranial and spinal compartments. We consider AMP changes during tilting to reflect partially the different intracranial volume variations taking place at the tested angles. Therefore, our results warrant further studies for investigating the potential of this non-invasive method to derive CC surrogates.

## Trigeminal neuralgia and normal pressure hydrocephalus: a rare association. Case report and review of the literature

### Fernando Hakim^1,2^, Juan F. Ramon^1,2^, Diego F. Gómez^1,2^, Juan A. Mejia^1,2^, Ericka Ramirez^1^, Salvador M. Mattar^1,2^

#### ^1^Department of Neurosurgery, Fundación Santa Fe de Bogotá, Bogotá, Colombia; ^2^Normal Pressure Hydrocephalus Center of Excellence, Bogotá, Colombia; ^3^Universidad de los Andes, Bogotá, Colombia

##### **Correspondence:** Fernando Hakim (fhakimd@gmail.com)

**Introduction:** NPH is a well-known entity described as an insidious syndrome that majorly affects patients over 65 years of age and causes gait abnormalities, urinary incontinence and a cognitive decline. Nevertheless, there are no records of a causal association with trigeminal neuralgia (TN). There are some reports of association of TN and hydrocephalus but there is no history of TN with NPH. To the date, the increase in intracranial pressure and its effect on the trigeminal nerve is the most accepted pathophysiological theory. In this document we review the literature on this unusual association.

**Methods:** We made a case report and a review of the literature. A systematic search of the literature was carried out resulting in thirteen case reports documenting this association in the literature. Patients with obstructive hydrocephalus secondary to tumors were excluded.

**Results:** We report the case of a woman of 57 years old with diagnosis of TN who received pharmaceutical treatment which failed, NPH was suspected and a tap test was performed, with a likelihood of NPH diagnosis and a ventricular shunt was done with improvement of symptoms. Additionally, we did a systematic search of the literature and found thirteen case reports documenting this association.

**Conclusions:** Although, there is no clear or accepted theory regarding the pathophysiology of the association between hydrocephalus and TN, the clinical evidence reviewed and the case now presented suggest hydrocephalus should be considered among the possible etiologies of TN syndrome. Consent to publish had been obtained.

## Diagnosing and subgrouping normal pressure hydrocephalus using independent component analysis

### Emanuele Camerucci^1^, Petrice M. Cogswell^1^, David T. Jones^2^, Jeffrey L. Gunter^1^, Jonathan Graff-Radford^2^, Jeremy K. Cutsforth-Gregory^2^, Benjamin D. Elder^3,4^, Matthew C. Murphy^1^, David S. Knopman^2^, Prashanthi Vemuri^1^, Ronald C. Petersen^2^, John Huston III^1^, Clifford R. Jack Jr^1^, Hugo Botha^2^

#### ^1^Department of Radiology, Mayo Clinic, Rochester (MN), USA; ^2^Department of Neurology, Mayo Clinic, Rochester (MN), USA; ^3^Department of Neurologic Surgery, Mayo Clinic, Rochester, MN 55905, USA; ^4^Department of Physiology and Biomedical Engineering, Mayo Clinic, Rochester, MN 55905, USA

##### **Correspondence:** Emanuele Camerucci (Camerucci.emanuele@mayo.edu)

*Fluids and Barriers of the CNS* 2022, **19(1)**

**Introduction:** Normal pressure hydrocephalus (NPH) is a treatable cause of gait and cognitive decline that can be challenging to diagnose. We hypothesized that unsupervised machine learning can use patterns of cerebrospinal fluid (CSF) distribution to differentiate NPH patients from controls and to distinguish between NPH phenotypes.

**Methods:** 104 patients with NPH and pre-shunt MRI and 104 age- and sex-matched controls were included. 20 patients and controls were held out as a test set. MPRAGE sequences were segmented and normalized to the Mayo Clinic Adult Lifespan Template (MCALT). Independent component analysis (ICA) was performed on the CSF segmentation maps. Visual grading of MRIs was done for markers of disproportionately enlarged subarachnoid space hydrocephalus (DESH): high convexity-tight sulci (HCTS), enlarged sylvian fissures (ESF), and ventriculomegaly. Participants were divided into four phenotypes: congenital (ventriculomegaly and diffusely narrow sulci), DESH (HCTS + ESF), HCTS only, and no HCTS.

**Results:** We identified 7 patterns of CSF distribution based on optimal ICA decomposition. These patterns appeared to capture clinically relevant CSF features. High weights on patterns with abundant CSF in the Sylvian fissures and lower CSF at the high convexity were predictive of NPH, and in particular the DESH and HCTS phenotypes. The congenital subgroup had higher weights on a pattern capturing all CSF spaces. The accuracy of automated NPH diagnosis vs. clinical was 92.2%/87.5% for train/test.

**Conclusions:** Data-derived patterns of CSF distribution allowed for high accuracy in diagnosing NPH. Different phenotypes were associated with different weights across patterns.

## Intrauterine spontaneous subdural hematoma with hydrocephalus

### Mohammed Nooruldeen Jabbar

#### Canadian Specialist Hospital – Dubai, UAE.

##### **Correspondence:** Dr Mohammed Nooruldeen Jabbar (mohammed.nooraldeen@yahoo.com)

*Fluids and Barriers of the CNS* 2022, **19(1)**

**Introduction:** Subdural hematoma in a new born baby is associated with a history of maternal trauma, complicated vaginal delivery, instrumental delivery, foetal/maternal thrombocytopenia, coagulopathy, hepatic disease, infection or using drugs during the pregnancy; but in the absence of the above, intrauterine subdural hematoma is a rare event.

**Methods:** We present a new born delivered to a healthy mother at 38 weeks by elective caesarean section after he had been diagnosed with macrocephaly by routine obstetric growth scan ultrasound at 35 weeks of gestational age. The baby’s APGAR score was 4 at 1 min, he was pale, his fontanel was tense and his Occipitofrontal circumference was 46 cm, therefore, he was intubated immediately and transferred to Neonatal Intensive Care Unit. Brain Computed tomography revealed huge right sided subacute subdural hematoma that was almost occupying the entire right hemicranial space and severely compressing the underlying brain tissue. In addition, there was marked dilatation of the left lateral ventricle.

**Results:** The subdural hematoma was evacuated by two burr holes surgery, followed by repeated subdural tap. Intraventricular haemorrhage treated by repeated ventricular tap until Cerebrospinal Fluid became clear, then ventriculoperitoneal shunt surgery was done for him.

The baby was discharged from the hospital in stable condition and he is on continues follow up in outpatient clinics.

**Conclusions:** Spontaneous intrauterine subdural hematoma with hydrocephalus is a rare cause of macrocephaly that could be treated by good cooperation among obstetrician, neonatologist and neurosurgeon, but needs more study to find the cause of this condition so we can prevent its occurrence.

## Positional intracranial pressure monitoring in patients diagnosed with intracranial hypo- and hypertension

### Gwendolyn Williams^1^, Michael Meggyesy^1^, Enoch Kim^2^, Richard Um^1^, Dipankar Biswas^1^, Aruna Rao^1^, Mark G. Luciano^1^

#### ^1^Department of Neurosurgery, Johns Hopkins University School of Medicine, Baltimore, MD, 21205, USA; ^2^Nova Southeastern University Dr. Kiran C. Patel College of Allopathic Medicine, Fort Lauderdale, FL, 33314, USA

##### **Correspondence:** Gwendolyn Williams (gwilli85@jhmi.edu)

*Fluids and Barriers of the CNS* 2022, **19(1)**

**Introduction:** Positional symptoms are used to guide clinical decision making in cerebrospinal fluid (CSF) leak and hydrocephalus patients. A medical history of complex headaches in patients with potential CSF disorders warrants intracranial pressure monitoring (ICPm) at Johns Hopkins hospitals. To better understand the relationship between symptomatology, intracranial pressure (ICP), and body position, we investigated the slope of ICP between various body positions. In this study, the working hypothesis was that hypertensive or hypotensive intracranial pressure would have significantly different rates of ICP change at various body positions.

**Methods:** A single center retrospective chart review was performed for 268 patients who underwent ICP monitoring using a Codman Microsensor® ICP Transducer, grouped by previously known hydrocephalus related diagnosis. The data was acquired at the Johns Hopkins Cerebral Spinal Fluid Center within the departments of Neurosurgery and Neurology, between June 2016 and June 2021. Patient ICP was monitored at 10, 20-, 30-, 45-, and 60-degree head of bed. The slope of ICP between discrete head of bed tilt positions and linear regressions curves was analyzed.

**Results:** The slope of ICP from 45 to 60 degrees was significantly different (p < 0.05) between confirmed hypotensive and hypertensive patients.

**Conclusions:** The amount of change in ICP in body position may be a way to characterize brain compliance in different diseases.

## Clinical and genetic findings in normal pressure hydrocephalus (NPH) patients with possible mutations in the CWH43 gene

### Philip W. Tipton^1^, Merve Atik^2^, Alexandra I. Beasley^2^, Gregory S. Day^2^, Sanjeet S Grewal^3^, Kaisorn L. Chaichana^3^, Olga P. Fermo^2^, Zachary S. Quicksall^3^, Joseph S. Reddy^2^, Nilufer Ertekin-Taner^1,2^, Owen A. Ross^2^, Neill R. Graff-Radford^1^

#### ^1^Department of Neurology, Mayo Clinic Florida, Jacksonville, Florida, United States; ^2^Department of Neuroscience, Mayo Clinic Florida, Jacksonville, Florida, United States; ^3^Department of Neurosurgery, Mayo Clinic Florida, Jacksonville, Florida, United States

##### **Correspondence:** Neill R. Graff-Radford (graffradford.neill@mayo.edu)

*Fluids and Barriers of the CNS* 2022, **19(1)**

**Introduction:** Recently, loss of function (LOF) mutations in *CWH43* have been associated with NPH, and the *CWH43* knockout mouse model presents with hydrocephalus. In this study we replicated these findings, identified other possible *CWH43* mutations, and defined the clinical phenotype.

**Methods:** We performed whole genome sequencing (WGS) on 94 NPH patients and compared *CWH43* gene variants to WGS data of 982 Biobank volunteers. Single-nucleotide variants were identified via Golden Helix SNP & Variation Suite v8.8.3, validated and assessed with clinical features of carriers.

**Results:** Fifteen of 94 patients had *CWH43* variants that could affect the protein function. Ten patients had LOF mutations as describe previously and five carried missense point mutations. Two previously identified mutations, p.Leu533Terfs (n = 8) and p.Lys696Asnfs (n = 2) were twice as frequent in cases (carriers 11%) versus controls (6%). Combined with other rare variants p.Ile292Thr, p.Ala469Ser and p.Ala469Ser, mutations were significantly more frequent compared to the 982biobank cases. Twelve of 15 had head size measures. Of these 4/5 females had head size ≥ 57.5 cm and 6/7 males had head size ≥ 59 cm. Only 3/15 had some features of disproportionate enlarged subarachnoid hydrocephalus (DESH) and 3 with moderate vascular disease. 6/15 had shunt surgery and 5 improved.

**Conclusions:** *CWH43* mutations are frequent in NPH. Loss of function mutations are the most common, but missense mutations require further study. The clinical features include imaging of long-standing hydrocephalus without aqueductal stenosis and patients with large head size. When symptomatic they have typical NPH features and respond to surgery.

## What exactly happens to ICP and pulse amplitude when humans stand from sitting position?

### Eleanor M. Moncur^1,2^, Matthew M. J. Bancroft^2^, Linda D’Antona^1,2^, Sogha Khawari^1^, Simon Thompson^1^, Lewis Thorne^1^, Laurence D. Watkins^1^, Ahmed K .Toma^1,2^

#### ^1^Victor Horsley Department of Neurosurgery, National Hospital for Neurology and Neurosurgery, Queen Square, London, WC1N 3BG, UK; ^2^Department of brain repair and rehabilitation, University College London Queen Square Institute of Neurology, London, WC1N 3BG, UK.

##### **Correspondence:** Eleanor Moncur (e.moncur@nhs.net)

*Fluids and Barriers of the CNS* 2022, **19(1)**

**Introduction:** ICP changes with body position. The exact details of the change and the underlying mechanisms are not fully understood. Understanding the exact change could inform management of patients with CSF dynamics disturbances, as well as provide information needed to design a smart shunt.

**Methods:** Single-centre, prospective cohort study. Patients undergoing continuous intraparenchymal intracranial pressure (ICP) monitoring were fitted with continuous position sensors and underwent a set sequence of movements including sit-stand transitions for set periods of time. ICP and postural data were recorded at 100 Hz and synchronised. Data were analysed for ICP and pulse amplitude (PA) as well as wave form changes during actual movement. The effect of shunting and various pathologies was explored.

**Results:** 49 patients (male = 14; mean age 43 ± 14) were recruited. 15 patients had shunts. The average change in ICP from sitting to standing was 1.9 ± 8.9 mmHg and the average change in PA was 0.0 ± 4.7 mmHg. There was a transient pressure wave which occurs at the time of movement. The average magnitude of this wave during sitting to standing transitions was 4.9 ± 4.3 mmHg and during standing to sitting transitions this was 4.3 ± 3.8 mmHg. We present the effect of shunting and pathological conditions.

**Conclusions:** ICP increases slightly when we stand from sitting position. Transitioning between sitting and standing position creates a transient wave in ICP which then returns to a similar pre-movement ICP. This differs from other movement transitions.

## Combined endoscopic third ventriculostomy and lumboperitoneal shunt surgery in the elderly patient with complex hydrocephalus: a mixture of late-onset obstructive hydrocephalus and communicating hydrocephalus

### Ki-Su Park^1^, Sang-Youl Yoon^1^, Kyunghun Kang^2^, Myong Hun Hahm^3^, Eunhee Park^4^, Mi Ju Kim^5^

#### ^1^Department of Neurosurgery, School of Medicine, Kyungpook National University, Daegu, Republic of Korea; ^2^Department of Neurology, School of Medicine, Kyungpook National University, Daegu, Republic of Korea; ^3^Department of Radiology, School of Medicine, Kyungpook National University, Daegu, Republic of Korea; ^4^Department of Physical and Rehabilitation Medicine, Kyungpook National University Medical Center, Daegu, Republic of Korea; ^5^Department of Obstetrics and Gynecology, Kyungpook National University Hospital, Kyungpook National University School of Medicine, Daegu, Republic of Korea

##### **Correspondence:** Ki-Su Park (kiss798@gmail.com)

*Fluids and Barriers of the CNS* 2022, **19(1)**

**Introduction:** Late-onset obstructive hydrocephalus due to aqueductal stenosis (AS) in the elderly patient is very rare. Although the first treatment for obstructive hydrocephalus due to AS is endoscopic third ventriculostomy (ETV), there are cases in which ETV alone cannot be used.

**Methods:** We presented 3 cases of treatment using combined ETV and lumboperitoneal shunt surgery (LPS) in elderly patients over 75 years of age with this rare complex hydrocephalus with a mixture of late-onset obstructive and communicating hydrocephalus.

**Results:** Two patients were treated with ETV, firstly. However, symptoms worsened again. Therefore, additional LPS was performed. The other patient was treated with ETV and LPS at the same time. After the LPS, symptoms improved, and there have been no sequelae.

**Conclusions:** Combined ETV and LPS surgery may be a new alternative treatment for the combination of late-onset obstructive and communicating hydrocephalus in elderly patients.

## Determining the relationship between breathing, biofeedback, attention and intracranial pressure: an experimental pilot study

### Anand S. Pandit^1^, Danyal Z. Khan^1^, Eleanor Moncur^1^, Lewis Thorne^1^, Laurence Watkins^1^, Ahmed K. Toma^1^

#### ^1^Victor Horsley Department of Neurosurgery, National Hospital for Neurology and Neurosurgery, London, WC1N 3BG, UK

##### **Correspondence:** Anand S. Pandit (a.pandit@ucl.ac.uk)

*Fluids and Barriers of the CNS* 2022, **19(1)**

**Introduction:** While the influence of the cardiac cycle on intracranial pressure (ICP) is well documented, the relationship between ICP and other physiological parameters remains less certain. We explore the effect of three interventions on ICP: (i) deep breathing, (ii) mindful attention and (iii) visual biofeedback, all considered to alter respiratory and/or cognitive function.

**Methods:** A single-centre prospective, pilot study was performed in adult patients undergoing 24-h ICP monitoring for suspected CSF dynamic disorders. A baseline reference was established for each patient, in which the patient was asked to sit comfortably without a task (6 min). Following a practice period, the three interventions (3-min each) were performed with a 2-min rest interval in between, and the experiment was repeated. A linear, repeated-measures mixed-effects analysis was carried out with heart and respiratory rate as fixed confounders and each patient as a random-effects variable, with each intervention compared against the baseline.

**Results:** Four patients were analyzed (3 females, mean age = 39.3 years [SD ± 28.7]). At baseline, the group average ICP was -0.35 mmHg (SD ± 2.07). After accounting for confounders and patient-level differences, deep breathing was independently associated with an ICP decrease of 0.88 mmHg (p = 0.07), mindful attention: a reduction of 1.29 mmHg (p = 0.02), and visual biofeedback: a reduction of 1.27 mmHg (p = 0.01). Aggregate, univariate comparisons demonstrated matching relationships and significance levels.

**Conclusion:** This study represents the first to investigate the effects of simple, physiological interventions, which can independently reduce ICP. Further, large-sample work is warranted to explore the mechanisms through which these effects are mediated.

## Experimental investigation of the influence of pathological blood dynamics on the CSF system with regard to normal pressure hydrocephalus

### Anne Benninghaus^1^, Florian Huber^1^, Alexander Müller^1^, Klaus Radermacher^1^

#### ^1^Chair of Medical Engineering, RWTH Aachen University, Germany

##### **Correspondence:** Anne Benninghaus (benninghaus@hia.rwth-aachen.de)

*Fluids and Barriers of the CNS* 2022, **19(1)**

**Introduction:** It is known that often the supplying arteries stiffen with age. In addition, increased pressure in the venous system has been measured in patients with Normal Pressure Hydrocephalus (NPH). The impact of these changes on cerebrospinal fluid (CSF) dynamics has not been analysed. Therefore, the aim of this study was to experimentally investigate the influence of age-related and pathological changes in blood dynamics on CSF dynamics to examine whether they contribute to the pathogenesis of NPH.

**Methods:** For sensitivity analysis, a validated in vitro model was used to alter parameters of blood dynamics while recording intracranial pressure (ICP) and cervical CSF flow. The investigated parameter settings, based on literature data, are an increase in stroke volume (SV) by 20–30%, a decrease in total cerebral blood flow (tCBF) by 10–30% and a decrease in arteriovenous delay (AVD) between 40 and 60%.

**Results:** The ICP amplitude increases with an elevation in SV and decreases with a reduction in tCBF and AVD. The spinal CSF flow also increases with elevated SV and decreases with a reduction in tCBF and AVD.

**Conclusions:** Increased ICP amplitudes and decreased cervical CSF flow are typical characteristics of patients with NPH. However, none of the parameter settings could induce both characteristics simultaneously. Thus, the parameters may favour the development of NPH, not as a single factor, but rather as a combination of several factors. In addition to the referred blood parameters, these include cranial and spinal (dynamic) compliance, resorption and flow resistance.

## Postoperative valve pressure adjustment for a long-term success following shunt in idiopathic normal pressure hydrocephalus

### F. Torregrossa^1^, G. Grasso^1^

#### ^1^Neurosurgical Unit, Department of Biomedicine, Neurosciences and Advanced Diagnostics (BiND), University of Palermo, Italy

##### **Correspondence:** Fabio Torregrossa (fabiotorregrossa00@gmail.com)

*Fluids and Barriers of the CNS* 2022, **19(1)**

**Introduction:** The standard treatment for idiopathic normal pressure hydrocephalus (iNPH) is surgical cerebrospinal fluid (CSF) diversion, most commonly through implantation of a ventriculoperitoneal shunt (VPS), to alleviate the typical symptoms related to this condition. The development of programmable-pressure shunt valve devices has reduced the major complications associated with the CSF drainage volume and appears to have increased shunt effectiveness. However, optimal valve pressure is crucial since, over time, symptoms can occur, especially gait can worsen following an initial improvement.

**Methods:** We describe our experience based on the observation of a cohort of 74 patients operated by VPS in whom modification of the valve range has been performed in a strict follow-up performed at 3, 6, and 12 months postoperatively and yearly thereafter for at least 10 years.

**Results:** Among 84 patients treated, changes in valve pressure were performed in 42 patients (51.2%). Pressure changing has been performed in 7 cases (16.7%) at 3 months, in 22 (52,3%) at 6 months, 7 (16.6%) at 1 year, and in 6 cases (14.3) at 2 years follow-up. Revisions resulted in clinical improvement in 94% of cases, especially in gait disturbance compared to the other symptoms.

**Conclusions:** Surgical treatment for iNPH by VPS is a safe modality able to improve symptoms in most affected patients even in the long term. Symptoms recurrency can be managed by valve range modification. This management, in our experience, has shown to improve quality of life and better long-term independent living expectations.

## Dimethyl sulfoxide-dependent ventriculomegaly. A new pathway that explains one etiology of hydrocephalus

### Leandro Castaneyra-Ruiz^1^, Seunghyun Lee^1^, Michael Muhonen^2^

#### ^1^CHOC Children’s Research Institute, Orange, CA 92868, USA; ^2^Neurosurgery department at CHOC Children’s Hospital, Orange, CA 9868, USA

##### **Correspondence:** Leandro Castaneyra-Ruiz (Leandro.Castaneyra.Ruiz@choc.org)

*Fluids and Barriers of the CNS* 2022, **19(1)**

**Introduction:** Dimethyl sulfoxide (DMSO) is a commonly used pharmaceutical for skeletal, urological, and inflammatory problems, and is also considered a universal solvent. It can efficiently dissolve both polar and nonpolar compounds. However, its neurotoxicity has not been studied. In a serendipitous finding, we discovered that DMSO induces hydrocephalus when injected into the ventricles. We hypothesized that DMSO's high affinity to water induces an active diffusion of parenchymatic fluid into the ventricles through the water channel aquaporin 4 (AQP4).

**Methods:** In vitro and in vivo experiments were performed to quantify fluid extravasation through perivascular AQP4. For in vivo experiments, DMSO was injected intraventricularly into two days old C57BL mice. In vitro experiments used 24 transwell plates with confluent brain endothelial cells from mice.

**Results:** Our results showed dose-dependent ventriculomegaly associated with DMSO intraventricular injection. The ventriculomegaly increased proportionally when 1, 3, or 5 ul/g of DMSO was injected into the ventricles and was attributed to fluid extravasation from the parenchymatic vessels to the ventricular system.

In vitro experiments confirmed DMSO's -dependent permeability through brain endothelial cells.

**Conclusions:** When injected into the ventricles of C57BL mice in the perinatal period, DMSO induces hydrocephalus. This is a newly discovered etiologic agent for the development of hydrocephalus. This finding should be considered when administrating pharmaceuticals with DMSO in pediatric patients.

## CSF to blood clearance differs substantially across individuals and patients with CSF disorders

### Markus Herberg Hovd^1^, Espen Mariussen^2,7^, Hilde Uggerud^2^, Aslan Lashkarivand^3,4^, Hege Christensen^1^, Geir Ringstad^5,6^, Per Kristian Eide^3,4*^

#### ^1^Section for Pharmacology and Pharmaceutical Biosciences, Department of Pharmacy, University of Oslo, Oslo, Norway; ^2^Norwegian Institute for Air Research, Kjeller, Norway; ^3^Department of Neurosurgery, Oslo University Hospital – Rikshospitalet, Oslo, Norway; ^4^Institute of Clinical Medicine, Faculty of Medicine, University of Oslo, Oslo, Norway; ^5^Division of Radiology and Nuclear Medicine, Department of Radiology, Oslo University Hospital Rikshospitalet, Oslo, Norway; ^6^Department of Geriatrics and Internal medicine, Sorlandet Hospital, Arendal, Norway; ^7^Department of Air Quality and Noise, Norwegian Institute of Public Health, Oslo Norway

##### **Correspondence:** Per Kristian Eide (p.k.eide@medisin.uio.no)

*Fluids and Barriers of the CNS* 2022, **19(1)**

**Introduction:** Variability in cerebrospinal fluid (CSF) to blood clearance may possibly affect the underlying pathophysiology in neurological disease and may be a source of under- or over-dosage of intrathecally administered drugs. We here aimed at characterizing the CSF to blood clearance of the intrathecally administered magnetic resonance imaging contrast agent gadobutrol (Gadovist, Bayer Pharma AG, GE) in order to establish a population pharmacokinetic model.

**Methods:** Gadobutrol served as a CSF tracer, distributing outside the blood brain barrier, thereby serving as surrogate tracer for pathways taken by several brain metabolites and drugs in CSF. We estimated CSF to blood clearance in patients with different CSF disorders, including symptomatic pineal and arachnoid cysts, tentative spontaneous intracranial hypotension, idiopathic intracranial hypotension, and different types of hydrocephalus. Patients with no verified CSF disturbance were denoted references.

**Results:** Population pharmacokinetic modelling based on 1140 blood samples from 161 individuals revealed marked inter-individual variability in pharmacokinetic profiles, including differences in absorption half-life (time to 50% of tracer absorbed from CSF to blood), time to maximum concentration in blood and the maximum concentration in blood as well as area under the plasma concentration time curve from zero to infinity. Moreover, the different disease categories of CSF diseases demonstrated different profiles.

**Conclusions:** The present observations may suggest that defining CSF to blood clearance can become a useful diagnostic adjunct for work-up of CSF disorders, and possibly for assessing clearance capacity of endogenous brain metabolites from CSF, as well as measuring individual CSF to blood clearance of intrathecal drugs.

## Comparison between intracranial compliance noninvasive measurement parameters and tap test results in patients with normal pressure hydrocephalus

### Gabriel A. S. Mendes^1,2^, Manoel J. Teixeira^2^, Gustavo Frigieri^3^, Cintya Hayashi^2,3^, Lissa Kido^3^, Fernando C. G. Pinto^2^

#### ^1^Hospital of the State Public Servant of São Paulo, Physiotherapy Nucleous, São Paulo, SP, Brazil; ^2^Cerebral Hydrodynamics Group, Department of Neurosurgery, Hospital das Clínicas, University of SãoPaulo, SP, Brazil; ^3^Brain4Care, São Carlos, SP, Brazil

##### **Correspondence:** Gabriel André da Silva Mendes (gabriel_mendes@usp.br)

*Fluids and Barriers of the CNS* 2022, **19(1)**

**Introduction:** Normal Pressure Hydrocephalus is a disease directly related to the change in brain compliance and consequent repercussions in the brain parenchyma. Invasive monitoring of parameters such as compliance and intracranial pressure proves to be reliable, especially for the prognosis in neurocritical patients. The present study proposes to observe the parameters obtained in a non-invasive sensor for monitoring intracranial compliance from the company Brain4care® in patients with suspected NPH and compare with the tap test result.

**Methods:** Twenty-eight patients submitted to the Tap test were evaluated, consisting of medical, radiological, physiotherapeutic and neuropsychological evaluations before and after puncture of 50 ml of CSF, as well as evaluation by the Brain4care® non-invasive intracranial compliance measurement device in the following positions: lying, sitting and standing, both statically and for 05 min each before and after lumbar puncture. The tap test result was compared to the time to peak and P2/P1 ratio parameters obtained by the device.

**Results:** Our results show that in the group where the Tap test was positive, there was a median P2/P1 ratio greater than 1.0, indicating a change in brain compliance. In addition, there was also a significant difference between patients with positive, negative and inconclusive results, especially in the lying position.

**Conclusion:** The non-invasive intracranial compliance device when used in patients lying down and standing up obtained parameters that suggest correspondence with the tap test result.

## Dynamic ADC analysis during cardiac cycle in positive and negative CSF tap test groups in possible idiopathic normal pressure hydrocephalus

### Mitsuhito Mase^1^, Toshiaki Miyati^2^, Naoki Ohno^2^, Tomoyasu Yamanaka^1^, Motoki Tanikawa^1^, Yusuke Nishikawa^1^, Harumasa Kasai^3^

#### ^1^Department of Neurosurgery and Radiology, Nagoya City University Graduate School of Medical Sciences, Nagoya, Japan; ^2^Faculty of Health Sciences, Institute of Medical, Pharmaceutical and Health Sciences, Kanazawa University, Kanazawa, Japan; ^3^Department of Radiology, Nagoya City University Hospital, Nagoya, Japan

##### **Correspondence:** Mitsuhito Mase (mitmase@med.nagoya-cu.ac.jp)

*Fluids and Barriers of the CNS* 2022, **19(1)**

**Background:** We previously reported that the apparent diffusion coefficient (ADC) obtained from diffusion MRI in the cerebral white matter significantly changed during the cardiac cycle, and this change (deltaADC) aided in the diagnosis of iNPH. It is unclear how deltaADC is associated with the CSF tap test response in “possible iNPH.”

**Method and Materials:** This study included 22 patients with possible iNPH who either showed symptomatic improvements (positive group, n = 17) and those without improvement (negative group, n = 5) after the CSF tap test. On a 1.5-T MRI, ECG-triggered single-shot diffusion echo planar images were obtained. The deltaADC was calculated from the maximum-minus-minimum ADC value of all cardiac phase images on a pixel-by-pixel basis. Then, the deltaADC, the mean ADC during the cardiac cycle (ADCmean), and the rates of change before and after CSF tap test of the white matter were determined and compared between positive and negative groups in possible iNPH.

**Results:** Before the CSF tap test, the deltaADC in the positive group was significantly higher than that in the negative group (P < 0.05), but there was no significant difference in the deltaADC between positive and negative groups after the CSF tap test. No significant difference was observed in the ADCmean between positive and negative groups both before and after the CSF tap test. The deltaADC change rate before and after CSF tap test in the positive group was significantly higher than that in the negative group (P < 0.05), but there was no significant difference in the ADCmean change rate before and after CSF tap test between positive and negative groups.

**Conclusion:** The deltaADC follows the CSF tap test response in possible iNPH. ΔADC analysis makes it possible to predict the CSF tap test response in possible iNPH.

## Clinical observation on the safety and efficacy of methazolamide in the treatment of normal pressure hydrocephalus

### Yan Xing

#### Department of Neurology, Aviation General Hospital, China Medical University, Beijing 100012, China

##### **Correspondence:** Yan Xing (drxingyan@163.com)

*Fluids and Barriers of the CNS* 2022, **19(1)**

**Introduction:** With the increasing number of patients with normal pressure hydrocephalus (NPH) identified, there is an urgent need to explore the pharmacological treatment of NPH. Clinical experience and research all have evidence that acetazolamide (AZA) can reduce the production of cerebrospinal fluid (CSF), but it is less used because of its side effects. Methazolamide (MTZ), similar in structure to AZA, has the advantages of high lipid solubility, easy penetration through the blood- brain barrier, and potential neuroprotective effects. Therefore, MTZ may be superior to AZA and become one of the effective methods in treating NPH. First, this study aimed to investigate the efficacy and safety of oral MTZ in patients with NPH to provide an alternative treatment for some inoperable NPH patients. The objective is to study the efficacy and safety of MTZ for the treatment of NPH patients.

**Methods:** A randomized, double-blind, placebo-controlled, prospective clinical study was conducted in Aviation General Hospital. A total of 35 NPH patients including 29 idiopathic normal pressure hydrocephalus (iNPH) and 6 secondary normal pressure hydrocephalus (sNPH) received drug treatment in our hospital from September 2019 to March 2021.All patients were unsuitable for or refused surgical treatment for some reasons. The patients were divided into drug group (n = 18) and control group (n = 10), taking oral MTZ or placebo 25 mg twice daily, increasing to 50 mg twice daily after 1 week if there was no discomfort. The 10 m gait score, cognitive function score, brain MRI check were completed before and 1 month after oral administration. The assessment of idiopathic normal pressure hydrocephalus scale (iNPHGS) score was performed 1 month and 3 months after oral administration. The primary efficacy endpoint was iNPHGS score for 3 months treatment and the secondary efficacy endpoint was the assessment of above scales for 1 month treatment.

**Results:** As compared with baseline, the effect of 1 month treatment showed that MOCA scores [(16.2 ± 8.8) and (14.8 ± 8.7) scores, t = -– 2.68, P = 0.02], 10 m gait scores [(22.3 ± 11.2) and (25.6 ± 12.9), t = 2.76, P = 0.02], iNPHGS scores [(7.3 ± 3.2) and (8.1 ± 3.5), t = 4.08, P < 0.01] were improved. The effect of 3 month treatment showed that the iNPHGS score (6.1 ± 2.4) was improved compared with baseline (t = 5.07, P < 0.01) and 1 month (t = 4.11, P < 0.01). But the above scores of the control group were not significantly improved compared with the baseline (all P > 0.05). After 1 month treatment, the 10 m gait score and iNPHGS score in the drug group were improved compared with those in the control group (all P < 0.05). After 3 months treatment, the iNPHGS score was improved compared with the baseline level in the control group (t = − 4.41, P < 0.05). The above 35 patients had no serious adverse reactions such as hypokalemia and acidosis. There was no significant difference in adverse events between the two groups (χ2 = 0.01, P = 1.00).

**Conclusions:** The treatment of MTZ could effectively improve the clinical symptoms of NPH patients with good safety.

## Treating hydrocephalus by shunting to the venous intracranial sinus—first in Human SinuShunt Project no.849502 supported by the European Union's Horizon 2020 Programme

### Sune Munthe^1^, Christian Bonde Pedersen^1^, Frantz Rom Poulsen^1^, Svend Erik Børgesen^2,1^

#### ^1^Neurosurgical Department, Odense University Hospital, Denmark; ^2^CSF‐Dynamics, Lyngby, 2800, Denmark

##### **Correspondence:** Sune Munthe (sune.munthe@rsyd.dk)

*Fluids and Barriers of the CNS* 2022, **19(1)**

**Introduction:** An ideal shunt for hydrocephalus treatment must maintain intracranial pressure within normal limits, drain the cerebrospinal fluid (CSF) without creating symptoms of over drainage independent of body position/physical activity, and present a long‐lasting solution without reoperation. All these criteria can be met by shunting CSF to the venous intracranial sinus. However, previous studies in this field have seen the drain outlet in the sinus become occluded. This study investigates an outlet designed to drain without occlusion or thrombus formation.

**Methods:** Patients diagnosed with hydrocephalus are shunted with a standard ventricular drain, low pressure uni‐ directional valve and the investigational outlet at the jugular foramen/sigmoid sinus. The outlet collapses into a standard introducer, placed using standard intravascular techniques. The outlet is held in the middle of the vein by a nitinol distancer, preventing it from touching the wall of the vein. Shunts are tested for patency at 3 months and at 6 months by water column test.

**Results:** As of May 2022, 12 patients have been operated, with 6 past the 6‐month endpoint. All shunts were patent at follow‐up, and clinical effect satisfactory. No subdural effusion or haematomas were detected. No occlusions of the sigmoid sinus or jugular vein were observed. No signs of lung emboli.

**Conclusions:** Initial results are promising: the nitinol frame prevents outlet occlusion without provoking thrombosis. The shunt remains open, indicating better than average shunt survival. This study represents an important first step in proving that shunting to the cranial sinus is a safe, simple and long‐lasting solution.

## The utility and reversibility of MRI biomarkers in predicting raised intracranial pressure

### Musa China^1^, Anand S. Pandit^2^, Hasan Asif^2^, Linda D’Antona^2^, Shivani B. Joshi^1^, Raunak Jain^1^, Crystallynn Skye The^1^, Arif H.B. Jalal^1^, Zakee Abdi^1^, Ptolemy D. W. Banks^1^, Fleur C. Yildirim^1^, Martyna K. Stasiak^1^, Yousif Aldabbagh^1^, Mohammad Alradhawi^1^, Emily B.C. Tan^1^, Ahmed K. Toma^2^

#### ^1^University College London, UCL, Division of Medicine, London, United Kingdom; ^2^National Hospital for Neurology and Neurosurgery, Victor Horsley Department of Neurosurgery, London, United Kingdom

##### **Correspondence:** Musa China (musa.china.15@ucl.ac.uk)

*Fluids and Barriers of the CNS* 2022, **19(1)**

**Introduction:** Intracranial pressure (ICP) typically requires invasive monitoring for accurate measurement. Non-invasive MRI biomarkers, including morphology of the pituitary, optic nerves and globe, are a useful tool in the identification of patients with raised ICP. We aim to determine the utility of brain MRI biomarkers in determining pathological ICP levels and their reversibility following CSF diversion.

**Methods:** A single-centre, retrospective cohort study. 327 patients (227 Female) underwent ICP monitoring with recent MR-imaging. Diagnoses included IIH (25%) and Chiari (8%). 5 MRI biomarkers were assessed: T1-sagittal views for pituitary: sella volume (Yuh grade), optic nerve vertical tortuosity and T2-axial views for optic nerve sheath distension (ONSD), posterior globe flattening (PGF) and optic disc protrusion (ODP).

**Results:** Median ICP for normal and abnormal sella morphologies were 3.73 and 7.04 mmHg, respectively (p < 0.05); normal and abnormal VT were 4.52 and 7.04 mmHg (p < 0.05); abnormal PGF were 4.51 and 10.35 mmHg respectively (p < 0.05). A baseline logistic model using all radiological parameters with age and sex as confounders predicted abnormal ICP with an AUC = 0.6857, sensitivity of 0.90 and specificity was 0.35. Pituitary Yuh grade and PGF were independently associated (p < 0.05, following multiple comparison correction). Furthermore, we found that pituitary sella grading and optic nerve VT were associated with significant pairwise reversibility (p < 0.05), following CSF diversion.

**Conclusions:** Specific radiological features are promising non-invasive markers associated with intracranial hypertension and also demonstrate reversibility following CSF diversion. Non-invasive MRI imaging can play a more significant role in the diagnostic workup of patients with possible CSF dynamic abnormalities.

## DRAIN – Double-blind Randomized Acetazolamide trial in Idiopathic Normal pressure hydrocephalus

### Johan Virhammar^1^, Madelene Braun^1^, Maria Ekblom^1^, Oskar Fasth^1^, David Fällmar^2^, Katarina Laurell^1^, Nanna MacAulay^3^, Dag Nyholm^1^

#### ^1^Department of Medical Sciences, Neurology, Uppsala University, Uppsala, Sweden; ^2^Department of Surgical Sciences, Radiology, Uppsala University, Uppsala, Sweden; ^3^Department of Neuroscience, University of Copenhagen, Copenhagen, Denmark

##### **Correspondence:** Johan Virhammar (johan.virhammar@neuro.uu.se)

*Fluids and Barriers of the CNS* 2022, **19(1)**

**Introduction:** There is no pharmacological treatment alternative for patients with idiopathic normal pressure hydrocephalus (iNPH). The aims are to investigate if the carbonic anhydrase inhibitor acetazolamide given to patients with iNPH improves gait function and to study the pathophysiological mechanisms leading to reduced symptoms.

**Methods:** Double-blind, randomized, placebo-controlled trial at a single center with the intent to include 50 patients. Randomization to placebo or acetazolamide 250 mg twice daily with treatment duration from diagnosis (baseline) to admission for shunt surgery. Primary outcome will be change in gait between study visits, measured with a combined score of 10 m gait test, timed-up-and go test and 3 m backwards walk. Biomarkers of neurodegeneration and brain injury will be measured in plasma and intraventricular CSF. MRI of the brain including Synthetic MR and perfusion sequences will be performed before and after treatment in a subgroup of 24–26 patients.

**Results:** Inclusion of patients started in February 2022.

**Conclusions:** The results of this study may form the basis for further, more extensive trials on pharmaceutical treatments for iNPH.

## The degenerative state of paraspinal muscle may affects gait improvement after shunt surgery in normal pressure hydrocephalus

### Ki-Su Park^1^, Sang-Youl Yoon^1^, Kyunghun Kang^2^, Myong Hun Hahm^3^, Eunhee Park^4^, Chaejin Lee^1^, Mi Ju Kim^5^

#### ^1^Department of Neurosurgery, School of Medicine, Kyungpook National University, Daegu, Republic of Korea; ^2^Department of Neurology, School of Medicine, Kyungpook National University, Daegu, Republic of Korea; ^3^Department of Radiology, School of Medicine, Kyungpook National University, Daegu, Republic of Korea; ^4^Department of Physical and Rehabilitation Medicine, Kyungpook National University Medical Center, Daegu, Republic of Korea; ^5^Department of Obstetrics and Gynecology, Kyungpook National University Hospital, Kyungpook National University School of Medicine, Daegu, Republic of Korea

##### **Correspondence:** Ki-Su Park (kiss798@gmail.com)

*Fluids and Barriers of the CNS* 2022, **19(1)**

**Introduction:** Paraspinal muscles play an important role in gait. This study aimed investigate whether the degenerative state of lumbar paravertebral muscles assessed by magnetic resonance imaging (MRI) in terms of the volume status and fatty degeneration could be related to the gait improvement after shunt surgery in NPH.

**Methods:** This is a retrospective analysis of 30 patients with NPH who underwent lumboperitoneal shunt surgery. The volume status and fatty degeneration of lumbar elector spinae muscles, multifidus muscles and psoas major muscles were measured by T2 weighted axial MRI at L2-3-4. The Timed Up and Go (TUG) test was performed to measure gait changes. The correlation between the status of paraspinal muscles and the gait improvement after shunt surgery in NPH was analyzed.

**Results:** Correlation analysis showed the significantly negative correlation between the psoas muscle index and the gait improvement after shunt surgery (*r* = − 0.280, *p* = 0.036). The fat infiltration rate of multifidus muscles in the poor improvement group was significantly higher than that in the better improvement group (*p* < 0.05).

**Conclusions:** Qualitative evaluation of the paraspinal muscles has the potential to reflect the gait prognosis after shunt surgery in normal pressure hydrocephalus.

## Efficacy, safety and cost analysis of the Sphera Pro Valve in the treatment of NPH patients

### Rodolfo C. Reis, Renata H. G. Yamashita, Davi J. F. Solla, Lais F. Ramin, Manoel J. Teixeira, Fernando C. G. Pinto

#### Cerebral Hydrodynamics Group, Department of Neurosurgery, Hospital das Clínicas, University of SãoPaulo, SP, Brazil

##### **Correspondence:** Fernando Campos Gomes Pinto (neurofernando@gmail.com)

*Fluids and Barriers of the CNS* 2022, **19(1)**

**Introduction:** Programmable valves provide an equal or superior neurological outcome when compared to fixed pressure ones, with fewer complications, in treating iNPH. Long-term costs of these treatments have not been properly compared in literature. The authors sought to compare efficacy, safety and costs of one-year treatment of iNPH patients with a novel programmable valve (Sphera Pro ®) with gravitational unit and a fixed pressure valve.

**Methods:** A prospective cohort of iNPH patients treated with programmable valve (G1) was compared to a historical cohort of iNPH patients treated with fixed pressure (G2). Our primary outcome was the mean cost of treating iNPH up to one year. Cost variables assessed included number of surgeries, length of ICU and hospital stay, and number of imaging tests. Efficacy in treating iNPH, measured by mean NPH Japanese Scale, and safety, measured by complications rates, were assessed as secondary outcomes.

**Results:** A total of 19 patients were analyzed in each group. Comorbidities and clinical presentation were similar between groups. Both G1 and G2 patients had neurological improvement over time (p < 0.001), but no difference was seen between groups. G2 had more complications than G1 (52.6% vs 10.5%, p = 0.013). Annual treatment cost per patient was US$ 3820 ± 2231 in G2 and US$ 3108 ± 553 in G1. Mean difference was US$712 (95% CI, 393–1805) in favor of G1.

**Conclusion:** The Sphera Pro® valve with gravitational unit had one-year treatment cost not higher than that of fixed-pressure valve, and resulted in similar efficacy and fewer complications.

## Outcomes following endoscopic third ventriculostomy in adults

### Suhaib Abualsaud^2^, Sebastian Yim^1^, Joseph Phoenix^1^, Marian Byrne^2^, Aimee Goel^2^, Luke Galloway^2^, Yasir Chowdhury^2^, Georgios Tsermoulas^2,3^

#### ^1^University of Birmingham Medical School, Birmingham, B15 2TT, United Kingdom; ^2^Department of Neurosurgery, Queen Elizabeth Hospital Birmingham, Birmingham, B15 2GW, United Kingdom; ^3^Institute of Metabolism and Systems Research, University of Birmingham, B15 2TT, United Kingdom

##### **Correspondence:** Suhaib Abualsaud (Suhaib.abualsaud@nhs.net)

*Fluids and Barriers of the CNS* 2022, **19(1)**

**Introduction:** ETV success score is frequently used to predict outcomes following ETV in adult patients, however this was a model developed for use in paediatric patients who often present with different causes of hydrocephalus compared to adults. We present a 9-year series of adult patients at a single tertiary Neurosurgical centre undergoing ETV for hydrocephalus to identify predictive factors in the adult population.

**Methods:** A retrospective study design was used to analyse 136 patients who underwent ETV between 2012 and 2020. Observed ETV success was compared to pre-operative predicted ETV success scores. A multivariable Bayesian logistic regression analysis was used to determine the factors that best predicted ETV success in our cohort of patients.

**Results:** Overall ETV success rate was 77%. Observed ETV success corresponded well with predicted ETV success using the ETV success score for the higher scores 80 and 90, but less well for lower scores. Aqueductal stenosis and a planned versus unplanned procedure were predictive of ETV success, whereas age, aetiology of tumour and location of CSF pathway obstruction other than aqueductal did not influence ETV success.

**Conclusions:** ETV was successful in approximately three quarters of adult patients with hydrocephalus. Obstruction at the level of the aqueduct of any aetiology is a predictive factor for ETV success. Age and other locations of obstruction have no bearing on ETV success. ETV success score is less reliable in the lower score categories, and there is a need for the development of ETV predictive tools more specific to adults.

## Psychiatric signs and symptoms in idiopathic normal pressure hydrocephalus (iNPH): a systematic review and meta-analysis

### Clara Belessiotis-Richards^1^, Esha Abrol^1^, Ahmed Toma^2^, Eileen Joyce^2^, Gill Livingston^1^

#### ^1^University College London, London, UK; ^2^National Hospital for Neurology and Neurosurgery (NHNN), Queen Square, London, UK

##### **Correspondence:** Clara Belessiotis-Richards (c.belessiotis@ucl.ac.uk)

*Fluids and Barriers of the CNS* 2022, **19(1)**

**Introduction:** The aim was to conduct a systematic review and meta-analysis of the psychiatric signs and symptoms in people with probable or possible idiopathic normal pressure hydrocephalus (iNPH), and estimate their prevalence.

**Methods:** We searched PUBMED/MEDLINE, Embase, Cochrane, PsycINFO and CINAHL from inception to search date, without language or study type restriction, including only peer-reviewed publications. We extracted key descriptive data including author, year, country, setting, participant number, age, sex, study design, diagnostic tool, classification of iNPH, timing of assessment, follow-up time, comparison group, number of cases, response rate. Outcome data included proportion of patients with symptoms or signs. We used I^2^ to assess heterogeneity and random effects meta-analysis to calculated pooled estimates.

**Results:** Our preliminary results found a pooled prevalence of 70% for apathy, 19% for anxiety, 19% for delusions, 6% for hallucinations, 21% for disinhibition, and 35% for agitation in iNPH. Our results for depression could not be meta-analysed due to heterogeneity; prevalence of depressive symptoms ranged from 8 to 56% across studies.

**Conclusions:** iNPH is associated with high rates of neuropsychiatric symptoms and signs. More work needs to be done to describe these and their response to treatment.

## Utility of neuropsychological tests in assessing response to cerebrospinal fluid drainage in idiopathic normal pressure hydrocephalus

### Elizabeth Cray^1^, Holly Roy^1,2^, Sharon Lau^3^, Aishah Hannan^3^, Abigail Beard^3^, Rupert Noad^3^, Samiul Muquit^1^, Samuel Jeffery^1^

#### ^1^Department of Neurosurgery, Southwest Neurosurgery Centre, University Hospital Plymouth, Plymouth, PL6 8DH, UK; ^2^Faculty of Health, University of Plymouth, PL4 8AA, UK; ^3^Department of Neuropsychology, University Hospital Plymouth, Plymouth, PL6 8DH, UK

##### **Correspondence:** Elizabeth Cray (elizabethcray@nhs.net)

*Fluids and Barriers of the CNS* 2022, **19(1)**

**Introduction:** Cognitive impairment is a key characteristic of idiopathic normal pressure hydrocephalus (iNPH) and may improve following cerebrospinal fluid (CSF) diversion. Neuropsychological tests can be used to aid diagnosis and assessment. The objective of this study was to assess the utility of these tests in patients undergoing CSF drainage.

**Methods:** A retrospective review was conducted of consecutive patients undergoing CSF drainage for suspected iNPH through a dedicated multidisciplinary (MDT) clinic. All patients underwent either high volume lumbar puncture (LP) or extended lumbar drain test (ELD) and had a repeatable battery of neuropsychological testing in memory, attention, motor speed, language, and executive function, along with a 10-m gait assessment. Following MDT evaluation, patients considered to be ‘responsive’ were offered a ventriculoperitoneal shunt.

**Results:** A total of 26 (15 male, 11 female, mean age 74 years) patients underwent CSF drainage with 20 (77%) demonstrating a positive response (13 following ELD and 7 following LP). Of the cognitive tests undertaken pre and post drainage, there was significant improvement in RBANS Immediate Story Recall (pre = 10.68, post = 13.21, p < 0.05), and Delayed Story Recall (pre = 3, post = 4.1, p < 0.05) but no significant change in WMS-III Mental Control, Trail Making Task, 9-Hole Pegboard or RBANS Semantic Fluency tests. Mean 10 m walk time improved from 26.51 s to 15.25 s (p < 0.05).

**Conclusions:** Tests of memory demonstrated significant change following CSF drainage whereas tests of other cognitive domains did not. Further investigation of the clinical utility of neuropsychological tests is planned.

## Normal pressure hydrocephalus: only subjective improvement after spinal tap test – is that enough for shunt indication?

### Maximilian Greiner-Perth, Martin Mersch, Uwe Kehler

#### Department of Neurosurgery, Asklepios Klinik Altona, Hamburg, Germany

##### **Correspondence:** Maximilian Greiner-Perth (m.greinerperth@gmail.com)

*Fluids and Barriers of the CNS* 2022, **19(1)**

**Objective:** The sensitivity of the spinal tap test (STT) for the diagnosis of a normal pressure hydrocephalus (NPH) is low. This may be due to insensitive tests, which are applied to prove the diagnosis. Often patients notice an improvement despite no change in the tests. Aim of this study is to show if subjective improvement only is sufficient for the diagnosis of NPH and consequently for the indication of a hydrocephalus shunt.

**Methods:** Patients after the STT were evaluated with objective gait tests and a questionnaire, where they had to log in their subjective gait changes regularly for a whole week. All patients who had an objective or only a subjective improvement got a ventriculo-peritoneal shunt. A follow-up was done between 1 and 3 years via a telephone interview.

**Results:** 86 patients who were evaluated with a STT. 52 showed an objective, 34 only a subjective improvement. All patients were shunted. Medium follow-up was 22 months. 77% (40 of 52) of the patients with objective improvement after STT showed a permanent improvement, whereas 82% (28 of 34) of patients improved with only subjective improvement after STT.

**Conclusion:** These results show, that subjective improvement alone after STT is as good as objective improvement for the diagnosis of NPH and for shunting. Therefore, using subjective improvements alone after STT may increase the sensitivity of STT for the diagnosis of NPH and for the indication for shunting.

## Ultra-Fast neuropsychological testing before and after tap-test in suspected iNPH patients: preliminary report

### Sara Fabbro^1,2^, Daniele Piccolo^1,2^, Ilaria Guarracino^3^, Barbara Tomasino^3^, Miran Skrap^2^, Francesco Tuniz^2^

#### ^1^Department of Neuroscience, University of Padua, Padua, Italy; ^2^Department of Neurosurgery, ASUFC Santa Maria della Misericordia, Udine, Italy; ^3^Scientific Institute IRCCS Eugenio Medea, San Vito al Tagliamento, Pordenone, Italy

##### **Correspondence:** Sara Fabbro (sarafabbro@libero.it)

*Fluids and Barriers of the CNS* 2022, **19(1)**

**Introduction:** iNPH is a primitive, progressive and partially reversible form of dementia with cognitive deficits in different domains (attention/orientation, memory, fluency, language and visuospatial abilities). Early treatment improves the outcome and suggests the importance for reliable diagnostic markers that accompany neuroradiological parameters and CSF dynamics invasive evaluations. The purpose of this exploratory study is to quantify the cognitive status of patients both before and after infusion and tap test.

**Methods:** From January 2020, neuroradiological, neuropsychological, CSF dynamic and tap test results of forty-one subjects were prospectively collected. Basic demographic data on age and sex were recorded at the point of referral. Evans’ index, callosal angle and DESH features were calculated on MRI sequences. R_out_, P_plateau_ and P_opening_, and neurological and neuropsychological response to deliquoration were collected. To quantify cognitive modifications after the test, we used Addenbrooke’s Cognitive Examination Revised (ACE-R) battery.

**Results:** The mean age of the population was 77.3 ± 5.5 years. Fourteen females and twenty-seven males composed the cohort. All patients demonstrated a statistically-significant impairment in all ACE-R domains before the test. Among the sub-test of the ACE-R, we founded a statistically-significant amelioration after the tap test for orientation (p = 0.019), indirect recall (p = 0.039) and naming (p = 0.018). We documented a significant correlation between P_plateau_ and post-test orientation (p = 0.017) and post-test naming (p = 0.009). P_opening_ value positively correlated with naming performance after the test (p = 0.037).

**Conclusions:** In our iNPH cohort, the ultra-fast battery demonstrated rapid neuropsychological changes, characterized by a statistically-significant improvement in orientation, naming and indirect recall after CSF subtraction.

## Assessment of cognition and psychological wellbeing in adults with idiopathic normal pressure hydrocephalus (iNPH)

### Lisa Healy^1^, Priya Varma^1^, Jeffrey Tooze^2^, Benjamin Dias Dougan^1^, Indu Lawes^1^, Rocío Fernández-Méndez^1,2^, Marek Czosnyka^2^, Peter Smielewski^2^, Alexis J. Joannides^1,2^

#### ^1^Division of Neuroscience, Addenbrooke’s Hospital, Cambridge, UK; ^2^Department of Clinical Neurosciences, University of Cambridge, UK

##### **Correspondence:** Lisa Healy (lisa.healy@addenbrookes.nhs.uk)

*Fluids and Barriers of the CNS* 2022, **19(1)**

**Introduction:** Prominent cognitive deficits in iNPH include processing speed, psychomotor speed, attention, and executive functioning. To facilitate cognitive assessment in routine practice, we sought to develop a brief cognitive screen and monitoring tool and assess the feasibility of its implementation.

**Methods:** Based on a systematic literature review, tests encompassing key deficits observed in iNPH were selected. These include validated tests completed by the patient (ACE-III, WAIS-IV Symbol Search, TMT-A, TMT-B, GAD-7, PHQ-9, WHO-QoL BREF and AES) and an informant (CBI-R). A convenience sample of patients and their clinicians rated acceptability and feasibility dimensions using visual analogue scales (0–10).

**Results:** Twelve patients attending a multidisciplinary iNPH clinic prior to treatment completed the battery. Administration took approximately 25 min. Most patients found the assessment enjoyable (92%) and of appropriate duration (83%), and were satisfied to be re-assessed in the future (100%). All clinicians (n = 3) found the battery clear, easy to administer, and time-efficient. Patients displayed deficits in attention, executive functioning, psychomotor and processing speed. Six patients scored below the ACE-III cut off for dementia. Patients reported low quality-of-life in at least three domains. There was considerable inter-patient variability in self-reports of anxiety, depression, and apathy.

**Conclusions:** Evidence-based, cost-effective assessment of cognition and psychological wellbeing in iNPH can inform diagnosis and monitor treatment outcomes. Preliminary data indicates the battery is sensitive to cognitive impairment, captures the specific cognitive changes observed in iNPH, and helps identify inter-patient variability enabling bespoke support. The battery was acceptable to patients and clinicians in a routine practice setting.

**Study supported by**: Revert Project, Interreg, France (Channel Manche) England, funded by ERDF.

## A repeated gait assessment protocol in the cerebrospinal fluid tap test for accurate prediction of effect of a shunt surgery on gait disturbances in idiopathic normal pressure hydrocephalus

### Takashi Suehiro^1^, Hideki Kanemoto^1^, Fuyuki Koizumi^1^, Shigeki Katakami^1^, Kayo Takeda^1^, Daiki Taomoto^1^, Yuto Satake^1^, Shunsuke Sato^1^, Tamiki Wada^1^, Maki Suzuki^2^, Kenji Yoshiyama^1^, Koichi Hosomi^3^, Haruhiko Kishima^3^, Hiroaki Kazui^4^, Etsuro Mori^2^, Manabu Ikeda^1^

#### ^1^Department of Psychiatry, Osaka University Graduate School of Medicine, Suita, Osaka, Japan; ^2^Department of Behavioral Neurology and Neuropsychiatry, Osaka University United Graduate School of Child Development, Suita, Osaka, Japan; ^3^Department of Neurosurgery, Osaka University Graduate School of Medicine, Suita, Japan; ^4^Department of Neuropsychiatry, Kochi Medical School, Kochi University, Kochi, Japan

##### **Correspondence:** Takashi Suehiro (suehiro-takashi@psy.med.osaka-u.ac.jp)

*Fluids and Barriers of the CNS* 2022, **19(1)**

**Introduction:** We examined the optimal gait measurement protocol for the CSF tap test.

**Methods** Subjects were definite iNPH patients who visited the Osaka University Hospital from April 2009 to October 2021. In our protocol, Timed Up and Go tests (TUG) were performed 4 times per day for 3 consecutive days before and after a CSF removal; total 24 times of TUG were conducted. We examined the beta coefficients of the following parameters with the change ratios (CR) of TUG scores between the baseline and 3 months after shunt surgery (CR-Shunt) by using univariate regression analyses; 1) CR of the best scores of TUG between before and after CSF removal (CR-Tap-Best); 2) CR of TUG scores in the initial trial between before and after CSF removal (CR-Tap-Initial); and 3) CR of the means of TUG scores between before and after CSF removal (CR-Tap-Mean).

**Results:** We enrolled 54 definite iNPH patients (mean (SD) age = 75.7 (5.6), female/male = 19/35). The initial, best and mean scores of TUG before CSF removal were 21.6 (13.6), 14.6 (4.7) and 19.0 (10.5) seconds, while those after CSF removal were 17.6 (14.4), 12.8 (4.0) and 15.0 (5.6) seconds. CR-Tap-Best, CR-Tap-Initial, CR-Tap-Mean and CR-Shunt were 10.3(10.6)%, 8.0(8.3)%, 21.3(20.5)% and 23.7(18.8)%. Univariate analyses revealed CR-Shunt was significantly correlated with CR-Tap-Best and CR-Tap-Mean (beta = 0.358/p = 0.011 and beta = 0.476/p < 0.001).

**Conclusions:** These findings indicate repeated assessments in the CSF tap test can well predict the improvement of gait after a shunt surgery.

## Thyroid hormones and health related quality of life in normal pressure hydrocephalus patients

### Mindaugas Urbonas^1,2^, Nijole Raskauskiene^2^, Vytenis Pranas Deltuva^1,2^, Adomas Bunevicius^2^

#### ^1^Department of Neurosurgery, The Hospital of Lithuanian University of Health Sciences (LSMU) Kauno klinikos, Eiveniu str. 2, LT-50009 Kaunas, Lithuania; ^2^Neuroscience Institute, Lithuanian University of Health Sciences

##### **Correspondence:** Mindaugas Urbonas (mindaugas.urbonas@lsmuni.lt)

*Fluids and Barriers of the CNS* 2022, **19(1)**

**Introduction:** This study evaluated the changes of thyroid hormones in iNPH patients before and after the ventriculoperitoneal shunting. We also sought to ascertain whether shunt implantation would result in the improvement of thyroid function and depression and anxiety.

**Methods:** Serum levels of FT3, FT4 and TSH were analysed among iNPH patients preoperatively, postoperatively and 3-months after the ventriculoperitoneal shunting. Preoperative FT3/FT4 ratio and its effect on outcome measured by PHQ-9 and GAD-7 preoperatively and 3-months after the shunting were analysed.

**Results**: 25 patients were included (52% women, mean age 63.5(SD9.5) years. Median Evan’s index was 0.42 (range 0.34–0.56). Preoperative Evan’s index was related to FT3 (r = 0.504, p = 0.017). The median (range) of preoperative FT3 and FT4 were 3.68 (3.24–4.47) and 15.48 (14.47–17.97) pmol/L, respectively. The median of TSH was 1.09 (0.24–4.79) mIU/L. FT3 levels were below normal value (< 3.34 pmol/L) in 24%, 64.5%, and 8% of the patients preoperatively, postoperatively and 3-months after the shunting, respectively (p < 0.001). When comparing preoperative and postoperative thyroid hormone profiles, significant decrease occurred in FT3 and TSH, while FT4 increased significantly (all p < 0.001). 3-months after the shunting thyroid hormones restored to the normal range. Preoperative higher FT3/FT4 ratio related to a poor 3-months outcome in depression (PHQ-9) and anxiety (GAD-7). The reported 3-months positive outcome rate in terms of depression and anxiety was 75%.

**Conclusion**: Our study demonstrated that some deficiencies of hypothalamic-pituitary-thyroid axis may improve at 3-months after the ventriculoperitoneal shunting among patients with iNPH. Increased preoperative FT3/FT4 ratio was associated with reduced quality of life.

## Caregiver burden development in patients with normal pressure hydrocephalus before and after shunt placement: paradox of improvement

### Fernando Hakim^1,2^, Juan F. Ramon^1,2^, Diego F. Gómez^1,2^, Juan A. Mejia^1,2^, Andrés D. Ramírez^1,3^, Salvador M. Mattar^1,2^

#### ^1^Department of Neurosurgery, Fundación Santa Fe de Bogotá, Bogotá, Colombia; ^2^Normal Pressure Hydrocephalus Center of Excellence, Bogotá, Colombia; ^3^Universidad de los Andes, Bogotá, Colombia

##### **Correspondence:** Fernando Hakim (fhakimd@gmail.com)

*Fluids and Barriers of the CNS* 2022, **19(1)**

**Introduction:** NPH is a progressively incapacitating illness that when treated can have an important impact on patients´ quality of life and independence. This improvement is bound to NPH´s reversible nature. In general, along with clinical improvement comes a diminishing of caregiver burden, nevertheless, in our experience, caregivers may be faced with new and more demanding challenges as a consequence of clinical improvement which brings a paradoxical increase in their burden.

**Methods:** We made a case series study. Patients with NPH diagnosis who underwent shunt placement surgery during 2017–2022, had clinical overall improvement of NPH symptoms and had a notable increase in their Zarit Burden Interview were included (n = 7). Each case was analyzed individually in order to identify the devolpment of symptoms after surgery as well as the caregiver´s burden.

**Results:** The patients analyzed had an improvement of at least 2 of the symptoms of the NPH triad and their caregivers experienced an increase of at least 5 points in Zarit Burden Interview. On average caregivers had a 10.7 points of increase. Caregivers report that the clinical improvement of the patients increased the risk of falling, and added.

**Conclusions:** Contrary to what is expected when positively impacting an incapacitating illness such as NPH, the caregiver burden may increase in caregivers of patients with NPH after shunt placement. The improvement of gait and cognitive impairment might worsen and/or develop new needs for patients and, as a consequence, add to the caregivers´ responsibilities.

## Relationships between cerebrospinal fluid biomarkers and the cerebrospinal fluid tap test in idiopathic normal pressure hydrocephalus

### Chunyan Liu^1^, Hongliang Li^1^, Qiong Yang^1^, Xing Liu^1^, Yan Xing^1^

#### ^1^Department of Neurology, Aviation General Hospital, Beijing, China

##### **Correspondence:** Yan Xing (drxingyan1@163.com)

*Fluids and Barriers of the CNS* 2022, **19(1)**

**Introduction:** Idiopathic normal pressure hydrocephalus (iNPH) is a neurodegenerative disease characterized by gait, cognition and urinary dysfunction. Since the pathophysiology of iNPH is related to aberrant CSF dynamics, the biomarkers associated with these changes may predict CSF-TT responsiveness. However, little is known regarding the correlation between lumbar CSF biomarkers and CSF-TT responsiveness in iNPH patients.

**Method:** A total of 146 iNPH patients were prospectively enrolled and subjected to CSF-TT. The levels of Aβ_42_, phosphorylated tau (P-tau_181_) and total tau (T-tau) in the CSF were analyzed by enzyme-linked immunosorbent assay (ELISA). Multiple groups were compared using ANOVA test.

**Results:** 71 patients were classified as CSF-TT responders and 55 as CSF-TT non-responders. The CSF P-tau_181_ levels were significantly lower in the CSF-TT responders compared to the non-responders (*P* = 0.018). The CSF-TT responders were further divided into 3 subgroups based on their gait and cognition (gait, gait + cognition and cognition). The P-tau_181_ level was significantly different across the non-responder and three responder subgroups (F=3.534, *P* = 0.016). The patients in the gait + cognition and cognition responder subgroups had lower levels of P-tau_181_ in their CSF compared to the non-responders (*P* = 0.009 and *P* = 0.015 respectively).

**Conclusion:** The P-tau_181_ level in the CSF of iNPH patients is related to their response to the CSF-TT. Lower levels of CSF P-tau_181_ correlated with a positive response to CSF-TT in the gait + cognition and cognition subgroups.

## Ventricular CSF biomarkers are associated with improvement after shunt surgery in iNPH

### Rebecca Grønning^1^, Anna Jeppsson^1^, Per Hellström^1^, Katarina Laurell^2^, Dan Farahmand^1^, Henrik Zetterberg^3,4^, Kaj Blennow^4^, Carsten Wikkelsø^1^, Mats Tullberg^1^

#### ^1^Hydrocephalus research unit, Department of Clinical Neuroscience, Institute of Neuroscience and Physiology, Sahlgrenska Academy, University of Gothenburg, Sweden; ^2^Department of Medical Sciences, Neurology, Uppsala University, Sweden; ^3^Clinical Neurochemistry Laboratory, Sahlgrenska University Hospital, Mölndal, Sweden, Department of Psychiatry and Neurochemistry, Institute of Neuroscience and Physiology, Sahlgrenska Academy, University of Gothenburg, Sweden; ^4^Department of Neurodegenerative Disease, UCL Institute of Neurology, Queen Square, London, UK and UK Dementia Research Institute at UCL, London, UK

##### **Correspondence:** Rebecca Grønning (rebecca.gronning@neuro.gu.se)

*Fluids and Barriers of the CNS* 2022, **19(1)**

**Introduction:** We aimed to explore the predictive value of a panel of biomarkers reflecting a range of pathophysiological processes in perioperative ventricular CSF (VCSF).

**Methods:** The Hellström iNPH scale was used pre- and 5 months postoperatively to quantify outcome in 119 consecutive patients diagnosed with iNPH. A postoperative increase of ≥ 5 iNPH scale points defined improvement. VCSF was collected during surgical intervention and analyzed for neurofilament light (NfL), glial fibrillary acidic protein (GFAP), amyloid beta 38 (Aß38), Aß40, Aß42, amyloid beta 42 to 40 ratio (Aß42/Aß40), soluble amyloid precursor protein alfa (sAPPα), sAPPß, total tau (T-tau), phosphorylated tau (P-tau), YKL40, monocyte chemoattractant protein-1 (MCP-1), growth-associated protein 43 (GAP43) and neurogranin. Group comparisons were performed using Mann Whitney U test. Linear regression models with log10 transformed biomarker concentrations as predictors and postoperative change in iNPH scale score as the dependent variable were controlled for age, sex and vascular risk factors.

**Results:** Neurogranin was higher in patients improved after surgery compared to patients not improved (median 365 (IQR 186–544) vs 330 (205–456); *p* = *0.046*). Aß42/Aß40 was bimodally distributed across all samples, as well as in the subgroups (cutoff 0.8). A regression model including neurogranin, T-tau and GFAP, resulted in adjusted R^2^ = 0.06, p = 0.047. Aß42/Aß40 did not contribute to outcome prediction.

**Conclusions:** A higher VCSF level of neurogranin, a marker of synaptic plasticity and regeneration suggested to have neuroprotective qualities, may signal favourable post-operative outcome. Concentrations of a panel of VCSF biomarkers explained 6% of the variability in outcome. Evidence of amyloid pathology did not affect outcome.

## Comparison between cognitive tests in the assessment of idiopathic normal pressure hydrocephalus

### Katarina Laurell^1,2^, Christian Andersson^2^, Johan Virhammar^1,2^

#### ^1^Department of Medical Sciences, Uppsala University, Uppsala, Sweden; ^2^Uppsala University hospital, Uppsala, Sweden

##### **Correspondence:** Katarina Laurell (Katarina.Laurell@neuro.uu.se)

*Fluids and Barriers of the CNS* 2022, **19(1)**

**Introduction:** Mini mental state examination (MMSE) or Montreal cognitive assessment (MoCA) tests are commonly used for screening of cognitive function. A battery of neuropshycological tests, designated for iNPH, can be used for more extensive evaluation (Hellström scale). In this study we aimed to compare MoCA and MMSE, and their agreement with the Hellström scale for assessment of cognitive function in patients with iNPH.

**Methods:** Patients under evaluation at the iNPH clinic in Uppsala during autumn 2020 were invited to participate. They performed the different cognitive tests during the same day. The occupational therapist performed Hellström and MMSE-tests, whereas the MoCA tests were conducted by two physicians. The interrater agreement was calculated.

**Results:** Fifty-four patients completed all tests. The total median score was lower on MoCA (23, IQR 6.25) than MMSE (26.5, IQR 6)(p < 0.001). Further, the patients achieved lower scores on MoCAs short term memory test and figure copy test, compared to the corresponding tests in MMSE (p < 0.001). A higher correlation to the neuropsychology part of the Hellström scale was found for the results of MoCA compared to MMSE (r_s_ = 0.833 vs r_s_ = 0.685). The intraclass correlation coefficient between raters was 0.995 (95% CI 0.976–0.999) for MMSE and 0.953 (95% CI 0.532–0.997) for MoCA.

**Conclusions:** The results of the neuropsychology domain of the Hellström iNPH scale correlated stronger to MoCA than to MMSE. In everyday clinical practice, an uncomplicated and timesaving test like MoCA could be an alternative in the assessment of cognitive impairment in iNPH.

## Incidence of idiopathic normal pressure hydrocephalus: a prospective population-based study

### Johanna Andersson^1^, Otto Lilja-Lund^1^, Anna Lindam^2^, Karin Kockum^1^, Katarina Laurell^3^

#### ^1^Department of Clinical Science, Neurosciences, Umeå university, Östersund, Sweden; ^2^Department of Public Health and Clinical Medicine, Family Medicine, Umeå University; ^3^Department of Neuroscience, Neurology, Uppsala University Hospital, Uppsala, Sweden

##### **Correspondence:** Johanna Andersson (jojjsi@hotmail.com)

*Fluids and Barriers of the CNS* 2022, **19(1)**

**Introduction:** The incidence of iNPH has mainly been estimated from clinical materials. One previous study from Japan calculated the annual incidence of iNPH among 70-years-olds to 1.2 /1000 persons (Iseki et al. 2014). In this prospective cohort study, we aimed to estimate the annual incidence of iNPH among Swedish inhabitants aged 65 years and older.

**Methods:** This study is part of a population-based prevalence study carried out 2014–2015, previously described by our group. A subgroup of 168 participants aged over 65 years, who had completed both imaging and neurological examination were re-invited for a two-year follow-up. Out of them, 122 underwent repeated examinations. Symptom assessment was made according to the NPH scale by Hellström (Hellstrom et al. 2012) and radiological evaluation according to iNPH Radscale (Kockum et al. 2018). The study participants were categorized according to the Japanese diagnostic guidelines 2nd edition.

**Results:** At baseline 130 participants were diagnosed as unlikely, 28 as possible, and 10 as probable iNPH. At follow-up ten participants had changed their diagnosis from unlikely to possible iNPH, corresponding to a cumulative incidence of 38.5/1000 persons over 1 year. In addition, one with possible iNPH changed to probable iNPH at follow-up. Those with probable iNPH remained in the same diagnostic category.

**Conclusions:** In this prospective study of a sample of individuals over 65 years old, the annual incidence of (possible) iNPH was 38.5/1000 person-years which is higher than previously described but in line with reports of a rapidly increasing prevalence with age.

## The impact of telesensors on neurosurgical service demand: a cohort cost-effectiveness analysis from institutional and patient perspectives

### Ptolemy D. W. Banks^1^, Anand S. Pandit^1^, Mohammad Alradhawi^2^, Yousif Aldabbagh^2^, Faith M. Y. Lee^2^, Eleanor M. Moncur^1^, Simon Thompson^1^, Lewis W. Thorne^1^, Laurence D. Watkins^1^, Ahmed K. Toma^1^

#### ^1^Department of Neurosurgery, National Hospital for Neurology & Neurosurgery, London, UK; ^2^UCL Medical School, University College London, London, UK

##### **Correspondence:** Ptolemy Banks (ptolemy.banks@nhs.net)

*Fluids and Barriers of the CNS* 2022, **19(1)**

**Introduction:** Implantable telemetric intracranial pressure sensors (telesensors) enable routine, non-invasive ICP feedback which can assist with clinical decision-making and attribution of pressure-related symptoms in patients with CSF shunt systems. Here, we aim to characterise telesensor cost-effectiveness and impact on service demand.

**Methods:** A single-centre, retrospective, cohort study and cost-effectiveness analysis of 80 patients (78% Female; 30% IIH, 22% Chiari malformation, 48% other) with MScio® (Christoph Miethke) telemetric ICP monitors. Service demand in the two years before and after implantation were retrieved from the centre’s electronic patient record system. Intentionally, data did not overlap with the COVID-19 pandemic period. The frequencies of hydrocephalus-related neurosurgical admissions, outpatient clinics, and scans were recorded along with A&E, neurology, and ophthalmology encounters. Tariffs were used to compare expenditure before and after implantation.

**Results:** Significant reductions were seen in the frequencies of neurosurgical admissions (1.9/year to 0.6; p < 0.001), ICP monitoring (0.4 to 0.01; p < 0.001), and CT scans (0.5 to 0.3; p = 0.013) following implantation. There were also significant reductions in the proportion of patients requiring admissions (91% to 45%; p < 0.001) and ICP monitoring (30% to 3%; p < 0.001). There were non-significant reductions in other invasive procedures, neurology encounters, and A&E admissions. Overall, there was a £341 (SD = 1069) per patient per year saving (22% reduction in included costs).

**Conclusions:** From an institutional perspective, the implantation of telesensors contributes to a reduction in service demand and a net financial saving. From a patient perspective, fewer appointments, invasive procedures, and radiation exposures suggest an improvement in patient experience and safety.

## Standardizing normal pressure hydrocephalus´ diagnosis and its impact on clinical outcomes: a single center experience

### Fernando Hakim^1,2^, Enrique Jimenez^1,2^, Juan F. Ramon^1,2^, Diego F. Gómez^1,2^, Juan A. Mejia^1,2^, Andrés D. Ramírez^1,3^, Salvador M. Mattar^1,2^

#### ^1^Department of Neurosurgery, Fundación Santa Fe de Bogotá, Bogotá, Colombia; ^2^Normal Pressure Hydrocephalus Center of Excellence, Bogotá, Colombia; ^3^Universidad de los Andes, Bogotá, Colombia

##### **Correspondence:** Fernando Hakim (fhakimd@gmail.com)

*Fluids and Barriers of the CNS* 2022, **19(1)**

**Introduction:** NPH is a challenging diagnosis since after 70 + years of its first scientific description there is still controversy regarding the standard indications for surgery. In our center we believe that a timely and justified diagnosis brings about positive outcomes. In order to improve the odds of the patient we offer a standardized, interdisciplinary evaluation prior and after lumbar puncture, as well as a yearlong follow-up after surgery. This paper aims to show the clinical outcomes of patients that undergo the protocol of the Center for Clinical care of NPH at FSFB.

**Methods:** We made a retrospective cohort-study. We included patients who underwent shunt placement surgery from 2018 to 2022 and attended to their 1 month follow-up (n = 36). We measured each symptom of the clinical triad individually and determined a positive overall outcome when patients improved in at least 2 of the 3 symptoms, or 1 of the 2 symptoms when they did not experience urinary incontinence.

**Results:** We found that 75% of patients improve their gait, 78.7% of patients improve their urinary incontinence, 80.5% improve in at least 1 and 66% improve in both of our cognitive tests. 86.1% of patients had a positive overall outcome and 58.33% of patients improve in all of the symptoms of NPH.

**Conclusions:** We consider that our positive results ratify the importance of a global approach to the diagnostic process of NPH. When properly evaluating the performance of patients we improve our chances of positively and notably impacting our patients’ quality of life.

## VIEshunt: towards a smart shunt system for hydrocephalus patients

### Fabian Flürenbrock^1^, Leonie Korn^1^, Dominik Schulte^2^, Anthony Podgoršak^2^, Nikolaos Tachatos^2^, Joris Chomarat^2^, Janina Hug^2^, Tiago Hungerland^2^, Caroline Holzer^2^, David Iselin^2^, Luca Krebs^2^, Rosina Weiss^2^, Markus Florian Oertel^3^, Lennart Stieglitz^3^, Mirko Meboldt^2^, Melanie Zeilinger^1^, Marianne Schmid Daners^2^

#### ^1^Institute for Dynamic Systems and Control, ETH Zurich, Switzerland; ^2^Product Development Group Zurich, ETH Zurich, Switzerland; ^3^Department of Neurosurgery, University Hospital Zurich, Switzerland

##### **Correspondence:** Fabian Flürenbrock (ffluerenb@ethz.ch)

*Fluids and Barriers of the CNS* 2022, **19(1)**

**Introduction:** Shunt systems for hydrocephalus patients are still based on passive mechanical pressure valves, which are driven by the gradient between intracranial pressure (ICP) and intra-abdominal pressure. However, this pressure gradient is influenced by a plethora of factors such as posture. Moreover, in case of normal pressure hydrocephalus, ICP is generally not increased. As a result, over- and underdrainage are common problems in shunt therapy.

**Methods:** We designed a ventricular intelligent and electromechanical shunt (VIEshunt) that consists of a micropump, a flow meter, a pressure sensor, an inertial measurement unit and an embedded control system. A posture dependent ICP reference is tracked by actively adjusting the drainage of cerebrospinal fluid (CSF) through the shunt. The shunt system has been evaluated on a hardware-in-the-loop (HIL) test-bench running real-time patient simulations.

**Results:** During HIL testing, ICP in the supine position was regulated from 15 to 12 mmHg. Subsequent postural changes from supine to upright and upright to supine position were robustly identified by the shunt system and the ICP reference was adjusted accordingly to -3 mmHg (upright) and 12 mmHg (supine). Posture-specific ICP references were tracked without offset, thus preventing adverse over- and underdrainage.

**Conclusions:** A smart shunt system has been developed that is capable of controlling CSF drainage and regulating ICP. In contrast to current shunts, our design allows for optimal physiological therapy by adapting to measured parameters of the patient’s pathophysiology. Long-term patient monitoring will improve medical care of the individual patient and enhance data-driven research into CSF dynamics.

## Development of cognitive symptoms in idiopathic normal pressure hydrocephalus: a longitudinal population-based study

### Otto Lilja-Lund^1^, Martin Maripuu^2^, Karin Kockum^1^, Johanna Andersson^1^, Lars Nyberg^3,4,5,6^, Katarina Laurell^7^

#### ^1^Department of Clinical Sciences, Neuroscience, Umeå University, Umeå, 901 87, Sweden; ^2^Department of Clinical Sciences, Psychiatry, Umeå University, Umeå, Sweden; ^3^Department of Radiation Sciences, Radiology, Umeå University, Umeå, Sweden; ^4^Department of Integrative Medical Biology, Umeå University, Umeå, Sweden; ^5^Umeå Center for Functional Brain Imaging, Umeå University, Umeå, Sweden; ^6^Center for Lifespan Changes in Brain and Cognition, University of Oslo, Norway; ^7^Department of Medical Sciences, Neurology, Uppsala University, Uppsala, Sweden

##### **Correspondence:** Otto Lilja-Lund (otto.lilja-lund@umu.se)

*Fluids and Barriers of the CNS* 2022, **19(1)**

**Introduction:** Idiopathic normal pressure hydrocephalus (iNPH) is a relatively common, progressive disorder most often debuting after 60 years. Little is known of how the cognitive symptoms develop in a population-based material.

**Methods:** Participants from the general population (*n* = 104, median [IQR] 75 years [72–80]) was followed for two years, of which 20 had possible iNPH (80 years [75–83) and 84 with unlikely iNPH (74 years [72–78]) at follow-up. Cognitive symptoms were assessed with Mini-mental State examination (MMSE) and a battery of neuropsychological tests (Hellström et al., 2012). Diagnosis was based on the Japanese guidelines (2^nd^ edition). Non-parametric tests with alpha-level *p* < 0.05 was conducted.

**Results:** The composite neuropsychological score was significantly worse in those with possible iNPH (75 [59–87]) compared to unlikely iNPH (83 [76–93], *p* = 0.013) at baseline and follow-up (possible 76 [58–67]; unlikely 85 [73–93], *p* = 0.044). MMSE were similar between the two groups at baseline (possible 29 [27–29]; unlikely 28 [27–30]) and follow-up (possible 27 [26–28]; unlikely 27 [26–28]).

**Conclusions:** Those with possible iNPH at follow-up had more cognitive symptoms already at baseline, and increased symptoms and reduced MMSE over time. Those with unlikely iNPH did not increase overall iNPH symptoms but had lower a lower MMSE score at follow-up. The MMSE could not detect baseline differences in cognition, whereas more sensitive neuropsychological tests could. Our results highlight the importance of careful supervision of older adults signaling reduced cognitive function.

## Chronic intracranial pressure monitoring for hydrocephalus using the Kitea system; first animal results

### Simon Malpas, Sarah-Jane Guild, Abdel-Hamed Dabbour, Natalia Lopez, Bryon Wright, Robert Gallichan, Dixon Leung, Daniel McCormick, Masahiro Kondo

#### University of Auckland, New Zealand

##### **Correspondence:** Simon Malpas (s.malpas@auckland.ac.nz)

*Fluids and Barriers of the CNS* 2022, **19(1)**

**Introduction:** It has long been proposed that a means to monitor intracranial pressure would provide a valuable way to detect when a shunt is failing in patients with hydrocephalus. Previous attempts at developing such technology have been unsuccessful due either to the size of the implant, its accuracy or difficultly in making measurements. We present our novel technology for obtaining ICP and our first animal validation experiments.

**Methods:** The Kitea system comprises a discrete microimplant measuring 2 × 3×20 mm which has an outer biocompatible glass housing and an array of internal micro-electronics including microprocessor for receiving power wirelessly, measuring pressure and transferring pressure waveforms at 50 Hz to an external wand. Sensors were inserted into the cortex of adult sheep using the same burr hole and surgical procedure used to place a shunt. Sheep were left for 6 months before being euthanized and tissue around the implants histologically analyzed for abnormal changes. ICP was measured in a subset of animals and compared to a catheter-based ICP measurement.

**Results:** After 6 months, we observed no signs of adverse histological responses and no sign of migration of the implant. Importantly, it was possible to wirelessly record ICP values in the conscious sheep that were within 1 mmHg of pressure obtained from catheter-based measurements.

**Conclusions:** The first results obtained from the Kitea system appear extremely promising.

## First experience with post-operative transcranial ultrasound through sonolucent burr hole covers in adult hydrocephalus patient

### Ryan P. Lee^1^, Jheesoo Ahn^1^, Michael Meggyesy^1^, Christina Ritter^1^, Ian Suk^1^, A. Judit Machnitz^2^, Judy Huang,^1^, Chad Gordon^3^, Henry Brem^1^, Mark G. Luciano^1^

#### ^1^Department of Neurosurgery, Johns Hopkins University School of Medicine, Baltimore, MD, 21205, USA; ^2^Department of Radiology, Johns Hopkins University School of Medicine, Baltimore, MD, 21205, USA; ^3^Department of Neuroplastic and Reconstructive Surgery, Johns Hopkins University School of Medicine, Baltimore, MD, 21205, USA

##### **Correspondence:** Ryan P. Lee (ryan.p.lee@jhmi.edu)

*Fluids and Barriers of the CNS* 2022, **19(1)**

**Introduction:** Managing patients with hydrocephalus requires repeated cross-sectional imaging. In adults, this is typically CT or less commonly MRI. However, CT poses cumulative radiation risks and MRI is costly. Ultrasound is a radiation-free, relatively inexpensive, and optionally point-of-care alternative, but is prohibited by limited windows through an intact skull. We describe our initial experience with transcutaneous transcranial ultrasound through sonolucent burr hole covers in post-operative CSF disorder patients.

**Methods:** Using cohort study design, infection and revision rates were compared between patients who underwent sonolucent burr hole cover placement during new ventriculoperitoneal (VP) shunt placement and endoscopic third ventriculostomy (ETV) over a one-year study time period and controls from the period one-year prior. Post-operatively, trans-burr hole ultrasound was performed in the clinic, at bedside inpatient, and in the radiology suite to assess ventricular anatomy.

**Results:** Satisfactory coronal ultrasound images of the ventricles were collected for all patients. Thirty-seven sonolucent burr hole cover patients were compared to 57 historical control patients. There was no statistically significant difference in infection rates between the sonolucent burr hole cover group (1/37, 2.7%) and the control group (0/57, p = 0.394). Revision rates were 13.5% versus 15.8% (p = 1.000), but no revisions were related to the burr hole or cranial hardware.

**Conclusions:** Trans-burr hole ultrasound is feasible for gross evaluation of ventricular caliber post- operatively in patients with sonolucent burr hole covers. There was no increase in infection or revision rate. This imaging technique may serve as an alternative to CT and MRI in the management of select hydrocephalus patients.

## Quantification of cardiac-related neural tissue motion in type 1 chiari malformation: a case control study pre- and post-spinal decompression surgery

### Gwendolyn Williams^1^, Dipankar Biswas^1^, Michael Meggyesy^1^, Audrey Fu^2^, Ari Blitz^3^, John Tew^4^, John Oshinski^5^, Francis Loth^6^, Nathan Schiele^7^, Bryn Martin^8^, Mark Luciano^1^

#### ^1^Department of Neurosurgery, Johns Hopkins University School of Medicine, Baltimore, MD, 21287, USA; ^2^Department of Statistical Science, University of Idaho, Moscow, ID, 83844, USA; ^3^Department of Radiology, School of Medicine, Case Western Reserve University, Cleveland, OH, 44106, USA; ^4^Department of Neurosurgery, University of Cincinnati Neuroscience Institute and University of Cincinnati College of Medicine, and Mayfield Clinic, OH, 45209, USA; ^5^Department of Radiology & Imaging Science and Biomedical Engineering, Emory University, Atlanta, GA, 30322, USA; ^6^Department of Mechanical and Industrial Engineering and Department of Bioengineering, Northeastern University, Boston, MA, 02115, USA; ^7^Department of Chemical & Biological Engineering, University of Idaho, Moscow, ID, 83844, USA; ^8^Alycone Therapeutics, Inc., Lowell, MA, 01852, USA

##### **Correspondence:** Mark Luciano (markluciano@jhu.edu)

*Fluids and Barriers of the CNS* 2022, **19(1)**

**Introduction:** Pathophysiology of Type I Chiari malformation (CMI) is not well understood but known to be a cerebrospinal fluid (CSF) related disorder. As CSF circulates with the cardiac cycle, pressure gradients between the CSF and cerebral blood flow (CBF) induce central nervous system (CNS) tissue motion which can result in tissue stretching and compression. We hypothesized that CNS tissue motion measurements would be abnormal in CMI patients and normalize after posterior fossa decompressive surgery.

**Methods:** Tissue motion in the rostral-caudal direction in three regions of interest were quantified with two-dimensional phase-contrast magnetic resonance imaging (2D PC-MRI) in nine CMI patients, before and after surgery, and compared with those in 10 healthy volunteers. Peak-to-peak differential displacement was quantified as the displacement of either the cerebellar tonsil or the pontomedullary junction relative to the upper spinal cord. A linear mixed effects model determined significance at the 0.05 level.

**Results:** We found significant differences (p < 0.05) between Chiari patients and controls in displacement of the spinal cord (Chiari: 0.51 ± 0.2 mm, Control: 0.72 ± 0.29 mm) and tonsil (Chiari: 0.25 ± 0.15 mm, Control: 0.14 ± 0.05 mm), but not within the pons (Chiari: 0.17 ± 0.07 mm, Control: 0.19 ± 0.03 mm). We did not see significant differences in displacement between Chiari patients pre- and post-operatively.

**Conclusions:** These results show cardiac-induced neural tissue motion in the rostral-caudal direction is altered in the CMI disease state compared to healthy controls however, this tissue motion was not normalized after surgery.

## 3D printed miniaturized ICP control valve for the treatment of hydrocephalus

### Seunghyun Lee^1^, Leandro Castaneyra-Ruiz^1^, Michael Muhonen^2^

#### ^1^CHOC Children’s Research Institute, Orange, CA 92868, USA; ^2^Neurosurgery department at CHOC Children’s Hospital, Orange, CA 92868, USA

##### **Correspondence:** Seunghyun Lee (Seunghyun.Lee@choc.org)

*Fluids and Barriers of the CNS* 2022, **19(1)**

**Introduction:** Most hydrocephalus is the communicating type, with the common etiology thought to be obstruction of CSF flow at the level of the arachnoid granulations (AGs). When treated with a shunting device, treatment failure mechanisms are often catheter related. The proposed project aims to address the failure mechanisms by developing a catheter-free completely passive miniaturized valve. The valve restores normal CSF drainage by bypassing defective AGs. By implanting the valve in the venous sinus/dura tissue, the need for persistent intracranial penetration via catheters is fully eliminated.

**Methods:** This self-seal valve is composed of two membranes fabricated by a 3D printer (Form 3 + , Formlabs). In its closed state the top membrane seals the perforations on the bottom membrane. The top membrane deflects upward to expose a conduit for fluid flow in the open state when ICP is higher than valve opening pressure. The valves are measured in a bench-top model to show its ideal hydraulic property for the treatment of hydrocephalus.

**Results:** The basic flow response of the valve was measured in our CSF fluidic circulatory setup. The valve showed a highly directional hydrostatic response with little reverse flow leakage. The reverse flow leakage was negligible, at approximately 1.0 µl/min on average. The valve opening pressure, P_T_, was approximately 50 mmH_2_O.

**Conclusions:** The proposed valve is fabricated by 3D printing technology and demonstrated the target hydrostatic characteristics for CSF drainage mechanisms, based on measurements on the bench-top model. This valve has three favorable design specifications: non-zero valve-opening pressure, negligible clogging and reverse flow leakage, and simplicity in design and fabrication process.

## What are the barriers to delivering timely CSF diversion in patients with NPH? Results of a single-centre service evaluation

### Shuler Men Xu^1^, Jonathan P. Funnell^2,3^, Lewis Thorne^2^

#### ^1^UCL Medical School, University College London, UK; ^2^National Hospital for Neurology and Neurosurgery, London, UK; ^3^Wellcome / EPSRC Centre for Interventional and Surgical Sciences, University College London, UK

##### **Correspondence:** Shuler Men Xu (shuler.xu1@nhs.net)

*Fluids and Barriers of the CNS* 2022, **19(1)**

**introduction:** Outcome of CSF diversion in Normal Pressure Hydrocephalus (NPH) patients is time-dependent; with earlier treatment associated with better outcomes. In this ongoing study, we aim to analyse the time period of various stages of NPH assessment and investigation from first referral to neurosurgery services, to shunt placement.

**Methods:** Retrospective review of electronic health records was performed for consecutive patients undergoing ventriculoperitoneal (VP) shunting for NPH over two consecutive months (February–April 2022). Demographic and clinical information was collected alongside dates of: referral sent to neurosurgery, referral received by neurosurgery, first outpatient review, and dates of interventions. Time interval between each consecutive step of our service provision was calculated to identify key time-limiting steps.

**Results:** Fifteen patients (mean age 75.6 (± 3.8) years) were identified, all undergoing insertion of a VP shunt. Mean lead time between sending of referral and VP shunting was 321(± 104) days. Of this, 17(± 16) days elapsed between referral sending and receipt, followed by 62(± 22) days until clinic review, and a further 229(± 75) days until shunt surgery. Patients undergoing extended lumbar drainage (LD) protocol waited 249 days from referral to shunting versus 188 days for those who proceeded directly to shunt (p = 0.62).

**Conclusions:** In this ongoing service evaluation, we established the mean waiting time from referral to shunt placement, identifying the longest lead time in the patient journey as between outpatient clinic and shunt placement. Since clinical improvement is time-dependent, we hope this study will help guide workflow optimisation in neurosurgical units to improve patient outcome following shunting.

## ‘Watkins’: a walking app for gait assessment in normal pressure hydrocephalus and decompensated long-standing overt ventriculomegaly patients

### Kanza Tariq^1^, Ahmed Toma^1^, Lewis Thorne^1^, Laurence Watkins^1^

#### ^1^National Hospital for Neurology and Neurosurgery, Queen Square, London, UK

##### **Correspondence:** Kanza Tariq (kanza.tariq@nhs.net)

*Fluids and Barriers of the CNS* 2022, **19(1)**

**Introduction:** The timed 10 m walking test is a frequently used assessment in normal pressure hydrocephalus (NPH) and decompensated long-standing overt ventriculomegaly (LOVA). We aimed to make a smart-phone app which performs the timed walking test and records the results for individual patients, thus making it possible for patients with a suitable smart phone to perform repeat assessments.

**Methods:** The iPhone built-in accelerometer was used to generate events for the app through CMMotionManager class. The non-purchase app is set at default 10 m, but the walking distance can be manually changed. When distance is set, the app gives verbal countdown notice of 3 s before it verbally instructs to ‘start walking’. When the pre-set distance is covered in a straight line, the app verbally informs the patient to ‘stop’, and displays the result in measure of distance covered, time taken to cover set distance and number of steps taken. This result can be saved and compared with future results.

**Results:** The app was validated in 50 subjects with timed slow-pace and fast-pace 10 m walking test. Compared to a clinical observer using a stopwatch, the app showed 100% accuracy in the measure of time taken to cover distance, 95% accuracy in the number of steps taken with an error ± 1–3 steps, and 97% accuracy in the measure of total distance covered with error of ± 0.25–0.50 m.

**Conclusion:** This is an efficient app for objective performance of timed walking test in NPH and LOVA patients.

## Impact of laparoscopic insertion of distal catheter of ventriculoperitoneal shunts on revision rates

### Sebastian Yim^1^, Joseph Phoenix^1^, Marian Byrne^2^, Ridwaan Sohawon^2^, Mahmoud Samara^2^, Andrew R. Stevens^2,3,4^, Simon C. Williams^5,6^, Georgios Tsermoulas^2,7^

#### ^1^University of Birmingham Medical School, Birmingham, B15 2TT, United Kingdom; ^2^Department of Neurosurgery, Queen Elizabeth Hospital Birmingham, Birmingham, B15 2GW, United Kingdom; ^3^NIHR SRMRC, University Hospitals Birmingham, Birmingham, B15 2GW, United Kingdom; ^4^Neuroscience, Trauma & Ophthalmology, Institute of Inflammation and Ageing, University of Birmingham, Birmingham, B15 2TT, United Kingdom; ^5^National Hospital for Neurology and Neurosurgery, London, WC1N 3BG United Kingdom; ^6^Wellcome/EPSRC Centre for Interventional and Surgical Sciences (WEISS), London, W1W 7TY, United Kingdom; ^7^ Institute of Metabolism and Systems Research, University of Birmingham, B15 2TT, United Kingdom

##### **Correspondence:** Sebastian Yim (sebastian.yim3@nhs.net)

*Fluids and Barriers of the CNS* 2022, **19(1)**

**Introduction:** Laparoscopic insertion of the distal tube of ventriculoperitoneal shunts is associated with lower incidence of catheter migration and malposition, compared to mini laparotomy^1^. We present a single-centre study on peritoneal catheter insertion following implementation of guidelines for VP shunt insertion, which among others promoted laparoscopic insertion, especially in the presence of obesity.

**Methods:** A retrospective analysis of shunt operations between May 2020 and August 2021 was performed to assess the impact of the surgical technique of the peritoneal catheter insertion on revision rates. Collected data included: primary or revision surgery, indication for shunting, surgical approach, previous and subsequent revisions, and patient demographics including body mass index (BMI). The revision rate of shunts inserted laparoscopically was compared to those inserted via a mini laparotomy.

**Results:** One hundred twenty nine patients underwent shunt insertion during the study period, out of which 16.3% of cases were performed laparoscopically. Laparoscopic insertion was associated with significantly lower revision rates compared to insertion via a mini-laparotomy (4.7 vs. 19.4%, *p* < 0.001). Mechanical failure was the primary indication for overall shunt revision. Patients with catheter migration and malposition had a higher BMI (37.08 vs. 30.67, *p* < 0.001).

**Conclusions:** The use of laparoscopy in ventriculoperitoneal shunt insertion was low in our series, but it was clearly associated with lower shunt revision rates. Patients with high BMI particularly benefit from laparoscopic insertion. Streamlining the surgical pathway for laparoscopic insertion of shunts is expected to lead to better surgical outcomes.

**References**

1. He, M., Ouyang, L., Wang, S., Zheng, M., & Liu, A. (2016). Laparoscopy versus mini-laparotomy peritoneal catheter insertion of ventriculoperitoneal shunts: a systematic review and meta-analysis. *Neurosurgical Focus*, *41*(3), E7.

## Link between cerebral blood flow and cerebrospinal compensatory parameters in NPH

### Marek Czosnyka, Zofia Czosnyka, Alexis J. Joannides, Peter Smielewski

#### Division of Neurosurgery, Department of Clinical Neurosciences University of Cambridge, Cambridge CB2 0QQ, UK

##### **Correspondence:** Marek Czosnyka (mc141@medschl.cam.ac.uk)

*Fluids and Barriers of the CNS* 2022, **19(1)**

**Introduction:** Hydrocephalus often involves failure of cerebrospinal fluid (CSF) circulation which can be accompanied by a cerebral blood flow (CBF) deficit. The nature of coupling between CSF circulation and CBF is poorly described, given that investigations require invasive monitoring of intracranial pressure and CBF. We sought to explore which components of CSF pressure–volume compensation correlate with global CBF.

**Method:** 36 adults diagnosed with hydrocephalus underwent an infusion test to assess CSF compensation, including measurement of baseline CSF pressure, pulse amplitude of CSF pressure (AMP), resistance to CSF outflow (Rout), cerebrospinal elasticity, the magnitude of slow vasogenic CSFp waves. Global CBF was estimated using a formula including Transcranial Doppler Ultrasonography flow velocity measurements, the slope of the regression line between AMP and CSF pressure from the period of CSF infusion and elasticity.

**Results:** Univariate analysis indicated a significant negative correlation between Rout and CBF (R = − 0.37; p = 0.035). Higher Rout (> 15 mmHg/(ml/min)) was observed when CBF was low (< 400 ml/min). There was also a significant correlation between CBF and the magnitude of slow vasogenic waves of CSFp at baseline (R = − 0.43; p = 0.013).

**Conclusion:** It is not known if the disturbance in CSF circulation causes a decrease in CBF or vice versa. Also, a decrease in the magnitude of slow vasogenic waves is associated with lower CBF. In traumatic brain injury, a low magnitude of slow ICP vasogenic waves is associated with poor outcome. In hydrocephalus, we do not see such an obvious link.

**Study supported by**: Revert Project, Interreg, France (Channel Manche) England, funded by ERDF.

## Cerebrospinal fluid closing pressure-guided tap test for the diagnosis of idiopathic normal pressure hydrocephalus

### Fernando Hakim^1,2^, Enrique Jimenez^1,2^, Juan F. Ramon^1,2^, Diego F. Gómez^1,2^, Juan A. Mejia^1,2^, Salvador M. Mattar^1,2^, Andrés D. Ramírez^1,3^

#### ^1^Department of Neurosurgery, Fundación Santa Fe de Bogotá, Bogotá, Colombia; ^2^Normal Pressure Hydrocephalus Center of Excellence; ^3^Universidad de los Andes, Bogotá, Colombia

##### **Correspondence:** Fernando Hakim-Daccach (fhakimd@gmail.com)

*Fluids and Barriers of the CNS* 2022, **19(1)**

**Introduction:** Tap test improves symptoms of idiopathic normal pressure hydrocephalus (iNPH); hence, it is widely used as a diagnostic procedure. However, it has a low sensitivity and there is no consensus on the parameters that should be used nor the volume to be extracted. We propose draining cerebrospinal fluid (CSF) during tap test until a closing pressure of 0 cm H2O is reached as a standard practice. We use this method with all our patients at our clinic.

**Methods:** This is a descriptive cross-sectional study where all patients with presumptive diagnosis of iNPH from January 2014 to December 2019 were included in the study. We used a univariate descriptive analysis and stratified analysis to compare the opening pressure and the volume of CSF extracted during the lumbar puncture, between patients in whom a diagnosis of iNPH was confirmed and those in which it was discarded.

**Results:** A total of 92 patients were included in the study. The mean age at the time of presentation was 79.4 years and 63 patients were male. The diagnosis of iNPH was confirmed in 73.9% patients. The mean opening pressure was 14.4 cm H2O mean volume of CSF extracted was 43.4 mL.

**Conclusions:** CSF extraction guided by a closing pressure of 0 cm H2O instead of tap test with a fixed volume of CSF alone may be an effective method of optimizing iNPH symptomatic improvement and diagnosis.

## Management of a shunt dysfunction case using non-invasive ICP waveform monitoring

### Tamires Guimarães Cavalcante Carlos de Carvalho^5^, Raphael Bertani^1^, Caio Perret^2^, Stefan Koester^3^, Paulo Santa Maria^2^, Savio Batista^4^, Nicolas Rabelo^1^

#### ^1^Department of Neurosurgery, University of São Paulo, São Paulo, SP, 01-246-000, Brazil; ^2^Department of Neurosurgery, Hospital Municipal Miguel Couto, Rio de Janeiro, RJ, 22430-160, Brazil; ^3^School of Medicine, Vanderbilt University, Nashville, TN, 37240, USA; ^4^Medical school, Universidade Federal do Rio de Janeiro (UFRJ), RJ, 20210-030, Brazil; ^5^Medical school, Universidade Nove de Julho (UNINOVE), São Paulo, SP, 01525-000, Brazil

##### **Correspondence:** Raphael Bertani, contato@rbertani.com

*Fluids and Barriers of the CNS* 2022, **19(1)**

**Introduction:** A 61-year-old patient was admitted to the Emergency Department with gait disturbance, behavioral changes, and urinary incontinence for 48 h which led to a mild TBI due to a fall from his own height. The family reported a 3-year history of diagnosis of intermittent pressure hydrocephalus, which was treated with a ventriculoperitoneal shunt using a programmable valve, also 3 years ago. A CT scan of the head was performed, which showed ventricles of normal dimensions, and a CT scan of the abdomen, which was inconclusive as to the integrity of the catheter. Abdominal ultrasound showed a small amount of free fluid.

**Methods:** We report a case of a patient with a programmable valve to treat intermittent pressure hydrocephalus whose diagnosis of valve dysfunction was aided by the brain4care® intracranial pressure waveform analysis device, allowing calculation of the P2/P1 ratio, without numeric values for ICP.

**Results:** The ICP waveform analysis through the brain4care® device showed a P2/P1 ratio of 1.6. Distal obstruction of the system was suspected. The patient underwent shunt revision. At 48 h postoperatively, the patient was asymptomatic, with a P2/P1 ratio of 1.0, The patient returned to clinic for follow-up, asymptomatic and an outpatient analysis showed a P2/P1 ratio of 0.6.

**Conclusions:** Seemingly simple cases of hydrocephalus may prove to be diagnostic challenges in the event of valve dysfunction, either by the differential diagnosis with infections or by nonspecific symptoms. Intracranial pressure waveform analysis can be effective as a diagnostic aid method, as well as a follow-up parameter. Consent to publish had been obtained.

## Evaluation of MRI-resistance of the Codman Certas Plus shunt valve in vivo

### Rafael T. Holmgren^1^, Martin Nilsson^1^, Peter Zsigmond^1^

#### ^1^Department of Neurosurgery and Department of Biomedical and Clinical Sciences, Linköping University, Linköping, Sweden

##### **Correspondence:** Rafael T. Holmgren (rafael.turczynski.holmgren@regionostergotland.se)

*Fluids and Barriers of the CNS* 2022, **19(1)**

**Introduction:** The Codman Certas Plus shunt valve was introduced in 2015. It has 8 different settings and was designed to resist the magnetic forces of 3 T MRI to avoid unintentional setting changes. In-vitro testing by the company has shown this to be the case. However, post-MRI check of the shunt setting is still recommended by the manufacturer. No study has yet shown the MRI-resistance of Certas Plus valves in a clinical setting.

**Methods:** 35 iNPH-patients (18 m/17f), median age 78(68–85) years, were operated with a Codman Certas Plus valve in this single-centre study. Valve setting was preoperatively randomized to 4 or 8 and blinded to the surgeon, patient and valve setting examiner. A 3 T MRI was performed 24–48 h postoperatively and valve setting was checked afterwards. Patients with setting 8 were adjusted to 4. Another 3 T MRI was done after 3 and 12 months with check of valve setting.

**Results:** A total of 68 3 T MRI are done of 35 Codman Certas Plus valves so far. All (100%) checked valve settings are accordance with the intended setting. There has been no unintentional setting change.

**Conclusions:** The MRI-resistance to unintentional valve setting changes of the Codman Certas Plus shunt valve is reliable in a clinical setting. This is in accordance with the experimental study done by the manufacturer. This has led to our institution abandoning the post-MRI check of the shunt setting making 3T MRI of these patients possible in regional hospitals without shunt adjustment tool kits and knowledge of the procedure.

## Vascular and morphological choroidal features in patients with idiopathic normal pressure hydrocephalus

### Nicola Valsecchi^1^, Matilde Roda^1^, Diana Wrona^1^, Giulia Marega^1^, Giorgio Palandri^2^, Giulia Giannini^3^, David Milletti^4^, Costantino Schiavi^1^, Luigi Fontana^1^

#### ^1^Department of Ophthalmology, IRCCS Alma Mater Studiorum University of Bologna, Bologna, Italy, 40138, ITA; ^2^Unit of Neurosurgery, IRCCS Istituto delle Scienze Neurologiche di Bologna, Bologna, Italy, 40138, ITA; ^3^Department of Biomedical and NeuroMotor Sciences (DIBINEM), IRCCS Istituto delle Scienze Neurologiche di Bologna, Bologna, Italy, 40138, ITA; ^4^Unit of Rehabilitation Medicine, IRCCS Istituto delle Scienze Neurologiche di Bologna, Bologna, Italy, 40138, ITA

##### **Correspondence:** Nicola Valsecchi (nicola.valsecchi2@studio.unibo.it)

*Fluids and Barriers of the CNS* 2022, **19(1)**

**Introduction:** The aim of the present study was to investigate vascular and morphological choroidal features in patients with idiopathic Normal Pressure Hydrocephalus (iNPH), before and after shunt surgery, compared to a control group.

**Methods:** The Bologna PRO-Hydro multidisciplinary team prospectively recruited 12 consecutive patients diagnosed with iNPH between November 2021 and March 2022 with indication for ventriculoperitoneal (VP) shunt surgery. An ophthalmic evaluation was conducted at the IRCCS Department of Ophthalmology at Hospital Sant’Orsola before and after a mean of 47.6 days (SD = 6.97) from shunting. Spectral-domain optical coherence tomography (SD-OCT) with enhanced depth imaging (EDI) was conducted. Images were binarized using the ImageJ software. Parameters studied were total choroidal area (TCA), luminal choroidal area (LCA), choroidal stromal area (SCA), sub-foveal choroidal thickness (SFT) and retinal nerve fiber layer (RNFL) thickness. Choroidal vascular index (CVI) was calculated as the ratio between LCA and TCA. Results were compared with 12 healthy, age-matched controls.

**Results:** SCA and SFT were significantly increased in iNPH patients after surgery compared to control group (1.2846 vs 0.975 mm^2^, 305 vs 204.5 mm, p = 0.031 and p = 0.019 respectively). CVI in iNPH patients was reduced compared to control group (62.73 vs 66.49%, respectively), though the difference was not statistically significant (p = 0.092).

**Conclusions:** iNPH patients treated with VP shunt showed an increased SCA and an increased SFT compared to control group. These results support the hypothesis that VP shunt surgery may determine hemodynamic and morphological alterations of the choroid in patients with iNPH.

## Successful early treatment for hydrocephalus due to aqueduct stenosis in a young patient

### Michiko Yokosawa^1^, Naoto Kimura^1^, Tetsuya Hayashi^1^, Hiroki Uchida^1^, Takayuki Sugawara^1^

#### ^1^Department of Neurosurgery Iwate Prefectural Central Hospital, Morioka-City, Iwate, 0200066, Japan

##### **Correspondence:** Michiko Yokosawa (hellomitty2004@yahoo.co.jp)

*Fluids and Barriers of the CNS* 2022, **19(1)**

**Introduction:** Aqueduct stenosis is considered congenital and acquired, but not well understood because it is a rare disease. Stenosis is often well tolerated for several years, but is not constant and is aggravated by meningitis, hemorrhage, or trauma and the like.

**Case presentation:** A 17-year-old male presented with severe headache. The headache was so severe that it interfered with his daily life. Magnetic resonance imaging (MRI) showed mild ventricular enlargement above the aqueduct. In addition, stenosis of the aqueduct was observed due to a thin septum. With no other cause of headache and the fact that the ventricles were enlarged for his age, we performed Endoscopic third ventriculostomy(ETV) and aqueductoplasty. Operation was performed under the general anesthesia. At first, The aqueduct stenosis was made up of several thin septal walls and performed aqueduct septostomy with fogarty balloon. Next, ETV was performed. The ventricles size was quickly decreased, and the headache improved after the surgery.

**Discussion:** When ventricular enlargement is mild as in this case, endoscopic manipulation is difficult due to the lack of structural dilatation; septostomy should be performed first because ETV first will further shrink the ventricles and make manipulation more difficult. Aqueduct stenosis is rare and is often detected in chronic hydrocephalus and cognitive decline. The present case was extremely rare case of early detection by headache. The optimal timing of treatment is uncertain when the ventricular enlargement is mild or asymptomatic cases. Consent to publish had been obtained.

## How I do it: insertion of ventriculo-thecal shunt

### Meriem Amarouche^1^, Lucia Darie^1^, Siegfried Sulim Adelhoefer^2^, Ahmed Toma^1^, Laurence Watkins^1^

#### ^1^Victor Horsley Department of Neurosurgery, National Hospital for Neurology and Neurosurgery, University College London Hospitals, London, UK; ^2^Universtitätsmedizin Berlin Charité

##### **Correspondence:** Lucia Darie (lucia.darie@nhs.net)

Meriem Amarouche and Lucia Darie contributed equally.

*Fluids and Barriers of the CNS* 2022, **19(1)**

**Keywords:** Chiari Malformation, Syringomyelia, Shunt, Ventriculo-thecal shunt

**Background:** The optimal treatment for Chiari Malformation I (CMI) patients remains controversial. Foramen magnum decompression is the most common accepted surgical procedure, however it carries a risk of interrupting the CSF flow across the cranio-cervical junction resulting in syringomyelia and subsequent neurologic deterioration. The syrinx cavity can be drained via syringo-pleural shunts, nevertheless with high failure rates. Following our internal protocol for CMI, raised intracranial pressure was excluded, leaving the alternative to equilibrate the pressure between cranial and spinal CSF compartments by inserting a ventriculo-thecal shunt.

**Method:** We describe the surgical procedure by which a ventriculo-thecal shunt is performed in a stepwise fashion.

**Conclusion:** Ventriculo-thecal shunts represent a feasible option in selected cases where a bypass is required to overcome an obstruction between cranial and spinal CSF compartments.

**Funding:** The authors have not received any funding for this article.

**Compliance with ethical standards:** Written consent was obtained from the patient for the publication of this case report and intra operative photography.

## Grading of ventricular reflux of intrathecal tracer – clinical experience utilizing a novel imaging biomarker of altered CSF flow in iNPH

### Per Kristian Eide^1,2^*,* Geir Ringstad^3^

#### ^1^Deptartment of Neurosurgery, Oslo University Hospital-Rikshospitalet, Oslo, Norway; ^2^Institute of Clinical Medicine, Faculty of Medicine, University of Oslo, Oslo, Norway; ^3^Department. of Radiology, Oslo University Hospital- Rikshospitalet, Oslo, Norway

##### **Correspondence:** Per Kristian Eide (p.k.eide@medisin.uio.no)

*Fluids and Barriers of the CNS* 2022, **19(1)**

**Introduction:** Today, magnetic resonance imaging (MRI) work-up of idiopathic normal pressure hydrocephalus (iNPH) is largely focused on anatomical derangements of cerebrospinal fluid (CSF) spaces. We introduced a MRI biomarker of CSF disturbance in iNPH, based on grading of ventricular reflux of an intrathecally administered MRI contrast agent (gadobutrol; 0.25 or 0.5 ml, 1 mmol/ml), serving as a CSF tracer. In ventricular reflux grades 3 and 4, CSF tracer remains in ventricles at 24 h after injection.

**Methods:** In iNPH patients, the functional imaging biomarker Ventricular Reflux Grade was compared with anatomical MRI biomarkers of CSF space anatomy (Evans’ index, callosal angle and disproportional enlargement of subarachnoid spaces hydrocephalus), imaging biomarkers of neurodegeneration (Schelten’s medial temporal atrophy scores, Fazeka’s scores and entorhinal cortex thickness), intracranial pressure (ICP) scores indicative of intracranial compliance, as well as clinical response to shunt surgery.

**Results:** Ventricular reflux grades 3–4 were related to findings of abnormal pulsatile ICP, indicative of impaired intracranial compliance, and also were frequent in shunt-responsive iNPH.

**Conclusions:** Grading of ventricular reflux of CSF tracer may add information to traditional imaging scores of CSF space anatomy by providing a functional measure of CSF disturbance in iNPH.

## Multi-objective framework for ventricular shunt catheter design

### Quentin Aten^1^

#### ^1^Integra LifeSciences, Mansfield, MA, 02048, USA

##### **Correspondence:** Quentin Aten (quentin.aten@integralife.com)

*Fluids and Barriers of the CNS* 2022, **19(1)**

**Introduction:** Ventricular catheters stimulate reactive neurological cells and tissues responsible for many shunt obstructions through mechanical, fluidic, and material stimuli. A multi-objective design framework is proposed for evaluating ventricular catheters.

**Methods:** Literature on ventricular catheter obstruction, ventricular anatomy, circadian rhythms of cerebrospinal fluid production, neuroglial cells’ fluid shear response, catheter placement forces, and optimization of catheter fluid dynamics were reviewed to establish design objectives. A standard ventricular catheter, a flow-balanced catheter, and an example novel design with elongated fenestrations were evaluated against these objectives. Three-dimensional computational fluid dynamics simulations quantified volumetric flow and fluid shear distributions.

**Results:** The design framework objectives for an ideal ventricular catheter are: (1) minimize parenchymal inflammatory response by minimizing catheter diameter; (2) maximize the distance between fenestrations and surrounding tissue; (3) maintain fluid shear below the critical shear for astrocyte IL-6 upregulation across CSF production rates; (4) maximize the distance cells must bridge when occluding fenestrations; (5) select a bio-inert catheter material; (6) withstand catheter insertion forces; and (7) be manufacturable on a commercial scale. As with many multi-objective problems, the conflicts inherent in this framework constrain the design space for improved ventricular catheters. The design with elongated fenestrations improved criteria 1,2,3,4 compared to the standard design, and criteria 3,4,7 compared to the flow-balanced design. For criteria 3, the elongated fenestrations reduced both maximum fluid shear and surface area experiencing critical shear compared to both standard and flow-balanced designs.

**Conclusions:** Future applications of this multi-objective catheter design framework may help reduce ventricular catheter obstruction.

## Shunt therapy in idiopathic normal pressure hydrocephalus: results from a long-term observation

### G. Grasso^1^, F. Torregrossa^1^

#### ^1^Neurosurgical Unit, Department of Biomedicine, Neurosciences and Advanced Diagnostics (BiND), University of Palermo, Italy

##### **Correspondence:** Giovanni Grasso (giovanni.grasso@unipa.it)

*Fluids and Barriers of the CNS* 2022, **19(1)**

**Introduction**: Idiopathic normal pressure hydrocephalus (iNPH) can be considered a treatable dementia since shunt therapy is effiient in improving symptoms. The long-term results of the cerebral spinal fluid (CSF) shunt have shown variable results without offering firm conclusions. Here we present the results of a retrospective study investigating the long-term results of iNPH patients treated with ventriculoperitoneal shunt (VPS) using programmable valves.

**Methods**: The symptoms before treatment were recorded. Post-VPS clinical symptoms and outcomes were assessed based on changes in gait, urinary incontinence, and cognitive dysfunction in a yearly follow-up spanning at least 10 years.

**Results**: Among a total of 65 patients treated. The median age was 71 years, and the mean follow-up time of the surviving patients was 140.5 ± 1.6 days. Overall, there was a significant and persistent improvement among all symptoms compared to the baseline (p < 0.05). Gait was the symptom with better and sustained improvement when compared with the baseline (p < 0.05). Mental impairment and urinary incontinence improved in the early follow-up (p < 0.05) followed by a reduction in the later follow-up. Twelve patients (18.4%) required surgical revisions for shunt malfunction. Change in valve pressure was performed in 35 patients (54%). Overall, 95% of revisions resulted in clinical improvement.

**Conclusions**: This study expands our previous results showing that surgical treatment for iNPH by VPS is still a safe modality that improves symptoms in most affected patients, even in the long term.

## ‘Watkins’ Point and trajectory’: description of a new method to guide freehand ventricular catheter insertion

### Kanza Tariq^1^, Meriem Amarouche^1^, Laurence Watkins^1^

#### ^1^National Hospital for Neurology and Neurosurgery, Queen Square, London, UK

##### **Correspondence:** Kanza Tariq (kanza.tariq@nhs.net)

*Fluids and Barriers of the CNS* 2022, **19(1)**

**Introduction:** Ventriculoperitoneal shunting is one of the commonest procedures performed in neurosurgery but there is no consensus on the best approach for ventricular cannulation. We describe ‘Watkins’ Point and trajectory’, a new method to guide parietal ventricular catheter insertion which relies on the patient’s anatomy rather than fixed measurements.

**Methods:** To assess the accuracy of Watkins’ Point in successfully achieving ventricular cannulation, we performed a preliminary study on 17 patients with an Evans’ Index (EI) of 0.35 or less and no pathology distorting their ventricular anatomy. We analysed CT Stealth scans obtained for clinical purposes using the free version of the OsiriX Dicom Viewer. On the 3D MPR view, we first ensured that the sagittal and coronal planes are midline and overlying the septum pellucidum. We obtained an axial image on the plane going from the lambdoid suture to a point above the orbital ridge. We identified the middle cranial point between the frontal and occipital inner tables in the mid-sagittal plane. We calculated a 45-degree angle away from the midline posteriorly and centred on the mid-cranial point. We then traced the ventricular catheter trajectory linking Watkins’ Point and the mid-sagittal point and confirmed it traverses the lateral ventricle.

**Results:** The patients had an EI between 0.2 and 0.35. A trajectory traversing the right lateral ventricle was successfully achieved in all patients using Watkins’ Point and the above described trajectory.

**Conclusions:** Watkins’ Point is a reliable entry site to achieve freehand ventricular cannulation in patients with EI above 0.2.

## Incidence of complications due to overdrainage in Normal Pressure Hydrocephalus and a new method to adjust the valve based on intracranial pressure

### Fernando Hakim^1,2^, Juan F. Ramon^1,2^, Diego F. Gómez^1,2^, Juan A. Mejia^1,2^, Salvador M. Mattar^1,2^, Daniel Jaramillo^1,2^, Martina Gonzalez^1,2^, Andrés D. Ramírez^1,3^

#### ^1^Department of Neurosurgery, Fundación Santa Fe de Bogotá, Bogotá, Colombia; ^2^Normal Pressure Hydrocephalus Center of Excellence, Bogotá, Colombia; ^3^Universidad de los Andes, Bogotá, Colombia

##### **Correspondence:** Fernando Hakim (fhakimd@gmail.com)

*Fluids and Barriers of the CNS* 2022, **19(1)**

**Introduction:** Overdrainage is a complication of CSF shunt in NPH. Includes subdural hematoma, subdural hygroma and postural headache; and has a frequency of up to 53%. We propose that the adjustment of the valve opening pressure (VOP) according to the Intracranial pressure (ICP) and adjusting the delta (ΔICP-VOP) between 0–10 mmH_2_0 could reduce the incidence of overdrainage.

**Methods:** Patients with NPH that underwent VAS between 2016 and 2020 were included. Two groups are described, first had an adjusted delta between 0–10 mmH_2_0 (DA), the second one had a different intraventricular delta (nDA) based on the criteria of the treating neurosurgeon. We describe demographic and clinical characteristics, the incidence of overdrainage and the effect of adjusting (DA) or not (NDA) the ΔICP-VOP for overdrainage development.

**Results:** A total of 51 patients were included. The mean age was 78.5 years, 68.63% was male. Complications of overdrainage was diagnosed in 14 patients (27.45%), subdural hygroma was the most frequent complication (15.69%), followed by nonsurgical subdural hematoma (65.88%), postural headache (3.92%), and surgical subdural hematoma (1.96%). Stratifying by delta, gross values suggest a lower incidence rate of overdrainage in the DA group; nevertheless, the difference was not statistically significant (p = 0.49).

**Conclusions:** We describe a VOP adjustment technique based on ICP, aiming for an ΔICP-VOP between 0–10 mmH2O. Our results suggest that this method reduces the incidence of overdrainage after VAS. We recommend that further prospective studies be conducted to evaluate this association.

## Preauricular tunneling in ventriculo-peritoneal (VP) shunt: is it a useful alternative?

### Patricia Barrio Fernández, Julio Gutiérrez Morales, Jesús Antonio Rodrígues Vera, Jose Ricardo González Alarcón, Cristian Leonardo Ortíz Alonso

#### Department of Neurosurgery, Central University Hospital of Asturias, Oviedo, Spain, 33011, Europe

##### **Correspondence:** Patricia Barrio Fernández (patribf@hotmail.com)

*Fluids and Barriers of the CNS* 2022, **19(1)**

**Introduction:** Hydrocephalus is a common neurological disorder, caused by a progressive acumulation of cerebrospinal fluid (CSF) within the intracranial space.

VP shunt systems are the mainstay therapy for this condition. Considered to be an easy surgery, it is one of the least time – consuming procedures but has significant potential for complications. Usually the tunneling is performed in the retroauricular región, being the hardest part of the surgery because the strong muscles in the posterior occipital región. The presented surgical technique is different by the proposal of a preauricular tunneling.

**Methods**: We have retrospectively reviewed the hospital records of 25 patients who received a VP shunt with this preauricular approach.

The surgical technique changes only when tunneling from the abdomen to cranial region. In the supraclavicular region we change the direction to the preauricular region, positioning the valve in the temporal area.

**RESULTS:** The only complication that we have found was an iatriogenic parotid fistula with spontaneous resolution.

**Conclusions:** Although VP shunt is one of the most common procedures performed by neurosurgeons worldwide, the are no references in literature reviewed about this subject. In contrast to the common procedure, this new one seems to be a quicker and effortless alternative. The anterior position of the valve can help the palpation of the flushing / pump reservoirs to obtain CSF or test the system when suspicion of shunt malfunction, or to change the opening pressure in adjustable devices.

## The NNI paediatric VPS prognostication model for 30-day non-technical shunt failure

### Jia Xu Lim^1^, Hui Ping Han^1^, Yi Wen Foo^1^, Wan Tew Seow^1,2,3^, Sharon Y. Y. Low^1,2,3^

#### ^1^Neurosurgical Service, KK Women’s and Children’s Hospital, Singapore; ^2^Department of Neurosurgery, National Neuroscience Institute, Singapore; ^3^SingHealth Duke-NUS Neuroscience Academic Clinical Program, Singapore

##### **Correspondence:** Jia Xu Lim (jiaxu.lim@mohh.com.sg)

*Fluids and Barriers of the CNS* 2022, **19(1)**

**Introduction:** Ventriculoperitoneal shunt (VPS) is the commonest surgery performed by paediatric neurosurgeons. Despite the progress made, shunt failures remain a significant issue. We aim to develop a NNI paediatric VPS (NNI–pVPS) prognostication model to predict shunt failures in hydrocephalus.

**Methods:** A retrospective review of 214 paediatric VPS was performed. Patients with complex hydrocephalus (multiloculated or > 1 ventricular catheter required) were excluded. Patient characteristics, hydrocephalus and shunt details, and outcomes were documented. Non-technical failure included infection, occlusion or other causes not related to malposition or incorrect assembly.

**Results:** Mean age at VPS insertion was 6 months with a mean follow up duration of 44 months. The commonest type of hydrocephalus was obstructive (n = 142, 66.4%) and the commonest etiology was tumour related (n = 66, 30.8%). Most shunts were primary insertions (n = 131, 61.2%) and valves used were mostly non-programmable (n = 182, 85.0%). The 30-day non-technical shunt failure rate was 9.3%; 9 infections (4.2%), 7 occlusions (3.3%), and 4 others (1.9%). The NNI–pVPS model derived included age ranges, hydrocephalus type and etiology, ventricular instrumentation, and other risk factors. This model has an AUC of 0.75 (0.64 – 0.86), with a sensitivity of 70.0% and specificity of 75.3%, and a positive and negative predictive value of 22.6% and 96.1%.

**Conclusions:** The NNI–pVPS model was able to effectively predict 30-day non-technical shunt failures and can be used to guide counselling and follow up strategies. External validation should be performed to confirm this finding.

## A pilot study: pediatric hydrocephalus knowledge assessment among females at a small rural hospital in Sudan

### Nidaa Ahmed^1^, Mai Mohamoud^2^, Safa Hamid^3^

#### ^1,2^5th Year medical student at University of Khartoum, Sudan; ^3^Senior Specialist Neurosurgeon, Assistant Professor, Department of Surgery, University of Khartoum, Sudan

##### **Correspondence:** Nidaa Ahmed (nidaamunir98@gmail.com)

*Fluids and Barriers of the CNS* 2022, **19(1)**

**Introduction:** Hydrocephalus is a worldwide disorder characterized by abnormal flow or rarely excessive production of cerebrospinal fluid, leading to the widening of the cerebral ventricular system. Previous research stated that limited knowledge affects the attitudes toward the patients and their outcomes. Hydrocephalus is a common neurosurgical disorder in Sudan. Most of the patients come from rural areas and present late with large heads and poor neurological status making their management difficult.

**Aim:** To investigate the females’ knowledge about pediatric hydrocephalus at a small rural hospital in Al Dambo village in Sudan.

**Methodology:** A Small Study aiming to assess the knowledge of the females et al. Dambo rural hospital was conducted through an interview questionnaire structured from the thorough literature review. data were collected and analyzed using online google forms.

**Results:** of the 30 females attending Al Dambo Rural hospital, 8 females were selected randomly to assess their knowledge regarding hydrocephalus in pediatric patients. 75% of the females knew the symptoms of hydrocephalus, and only 25% of them were aware of hydrocephalus causes. Also, half of the females didn’t know about hydrocephalus treatment or its preventative measures.

**Conclusion:** This small study demonstrated Poor knowledge regarding pediatric hydrocephalus among females at a rural hospital. hence, further wider studies are recommended to be conducted along with educative interventions to raise awareness and positively affect attitudes toward pediatric hydrocephalus patients and their management.

## Rescue treatment of biloculated and isolated fourth ventricle

### Borja Sanz Peña^1^, Irene Iglesias Lozano^1^, Jorge Tirado Caballero^1^

#### ^1^Department of neurosurgery, Hospital Universitario Puerta del Mar, Cádiz, 11008, Spain

##### **Correspondence:** Borja Sanz Peña (bsanzpena@gmail.com)

*Fluids and Barriers of the CNS* 2022, **19(1)**

**Introduction:** Isolated fourth ventricle is a rare complication in hydrocephalus patients. Different treatment strategies have been proposed for its management, like endoscopic aqueductoplasty or IV ventricle-shunting. However, the loculation of the IV ventricle can make the control of this entity difficult.

**Methods:** We present a case report of a biloculated and isolated fourth ventricle patient. We present the treatment strategies we performed to control this arduous pathology.

**Results:** A seven-year-old female patient affected by posthemorrhagic hydrocephalus and an isolated fourth ventricle that went through a failed endoscopic aqueductoplasty with a stent. Because of repeated isolation events and neurological deterioration with an increased size of the isolated IV-ventricle, she required IV-ventricle shunting. During the follow-up, the IV ventricle developed intraventricular septae, which loculated this cavity, causing progressive neurological impairment. Our first strategy was to perform open surgery to reconnect the two loculations of this isolated IV ventricle. Even though these septae were successfully opened on two occasions, the patient continued to develop new septae a few weeks after these open procedures. Because of this recurrent loculation, we decided to open both cavities and connect them to a single IV-ventricle shunt using a Y connector. After this final surgery, we managed to control this rare entity and loculations did not increase their size again.

**Conclusions:** Loculated and isolated fourth ventricle is a rare complication with very difficult management. We present a case that only improved after multiple surgeries and a final debridation and connection of both loculations to the same device. Consent to publish had been obtained.

## Spontaneous cutaneous rupture secondary to severe congenital hydrocephalus

### Van Euldem Battad^1^, Oliver Ryan Malilay^1^, Kevin Paul Ferraris^1^, Kenny Seng^1^

#### ^1^Section of Neurosurgery, Department of Surgery, Jose R. Reyes Memorial Medical Center, Manila, Philippines

##### **Correspondence:** Van Euldem Battad (vaneuldembattad@gmail.com), Oliver Ryan Malilay (olivermalilay@gmail.com), Kevin Paul Ferraris (kpferraris@gmail.com), Kenny Seng (kennyseng55@gmail.com)

*Fluids and Barriers of the CNS* 2022, **19(1)**

**Introduction**: Congenital hydrocephalus is one of the most common causes of neurosurgical consults globally. Management delays may cause unique complications such as spontaneous cutaneous rupture and cerebrospinal fluid leakage. This may occur frequently in low- to-middle income countries, where prenatal and neurosurgical healthcare services are less accessible; however, no such cases have been recorded in the Philippines.

**Methods:** We present two cases of severe hydrocephalus with spontaneous cutaneous rupture previously seen at our institution.

**Results:** The first case is a 7-month-old female with leaking CSF from a 1-cm spontaneous cutaneous rupture at the right anterior aspect of her anterior fontanelle. On admission, her head circumference was 85 cm. Her imaging showed markedly dilated lateral ventricles with pneumoventricle, and a widened anterior fontanelle. Her defect was sutured and she was treated with appropriate antibiotics. She was discharged once infection resolved but eventually expired at home. The second is a 3-year-old male who presented with leaking CSF from a cutaneous rupture at the anterior border of the posterior fontanelle. His head circumference was 92 cm, with a 4-cm scalp defect exposing the underlying ventricle. The defect was sutured at the emergency room. His family refused further diagnostics and treatment, and opted to bring him home, and was lost to follow-up.

**Conclusions:** Only a few case reports on spontaneous cutaneous rupture are available to guide its treatment, with none coming from the Philippines. Our cases establish the local incidence of this underreported complication and demonstrate that simple suturing and antibiotic treatment do not lead to desirable outcomes. Consent to publish had been obtained.

## Transition of hydrocephalus patients from paediatric to adult neurosurgery: a single institution experience

### Lucia Darie^1^, Simon Thompson^1^, Daisy Abbiss-Biro^2^, Jessica Mendall^2^, Shehzore Adil^2^, Lewis Thorne^1^, Ahmed Toma^1^ and Laurence Watkins^1^

#### ^1^Department of Neurosurgery, National Hospital for Neurology and Neurosurgery, University College London Hospitals, London, UK; ^2^Medical student at University College London Medical School, London, UK

##### **Correspondence:** Lucia Darie (lucia.darie@nhs.net), Simon Thompson (simon.thompson3@nhs.net), Laurence Watkins (laurence.watkins1@nhs.net)

*Fluids and Barriers of the CNS* 2022, **19(1)**

**Keywords**: Hydrocephalus, Transition, Paediatric neurosurgery, Adult neurosurgery, Shunt

**Introduction:** The transition process from paediatric to adult neurosurgery is an essential part of patient’s care. This study aims to report our institution’s experience and serve as ground for future improvement.

**Methods:** This is a single centre retrospective case series study. Data regarding current age, hydrocephalus etiology, number of shunt revisions in childhood and after transition, type of presentation (acute vs routine follow up), type of valve, length of follow up was collected.

**Results:** A total of 227 patients were included (mean 32 years, min 16- max 68 years; SD ± 11.9). 122 were transitioned from the nearest paediatric center, Great Ormond Street Hospital; 105 from other national and international hospitals. Common hydrocephalus etiologies were: post haemorrhagic 19.8%, Chiari II 12.7%, Aqueduct stenosis 12.7%, congenital 8.81%, post infectious 7% and tumor 5.2%. Hydrocephalus following hemisperectomy, associated with syndromic craniosynostosis or idiopathic intracranial hypertension was rare. In 16.7% (38 cases) no cause could be identified, despite review of available cross-sectional imaging, due to insufficient documentation. 171 (75.3%) patients were seen electively, 37 (16.2%) presented with suspicion of acute shunt dysfunction and 19 (8.3%) were referred by other specialists after the patients were lost to follow up. Patients presenting acutely were more likely to come from other centers 28/105 (26.7%) compared to 9/122 (7.37%) from Great Ormond Street Hospital (p < 0.005; Wilcoxon Signed Rank). 66/227 (29%) patients underwent at least a CSF diversion intervention in adulthood.

**Conclusion:** Hydrocephalus is a condition that requires lifelong neurosurgical follow up. A more structural approach towards transition is needed.

**Funding and disclosures**: The authors did not receive any funding for the completion of this work. The authors report no conflicts of interest

## Investigate of cerebrospinal fluid volumes to evaluate probable idiopathic normal pressure hydrocephalus

### Hongliang Li^1^, Chunyan Liu^1^, Hong Tai^2^, Youping Wei^3^, Taizhong Shen^3^, Qiong Yang^1^, Xing Liu^1^, Yan Xing^1^

#### ^1^Department of Neurology, Aviation General Hospital, Beijing, China; ^2^Department of medical imaging, Aviation General Hospital, Beijing, China; ^3^Department of Rehabilitation, Aviation General Hospital, Beijing, China

##### **Correspondence:** Yan Xing (drxingyan@163.com)

*Fluids and Barriers of the CNS* 2022, **19(1)**

**Introduction:** Idiopathic normal pressure hydrocephalus (INPH) is a potentially reversible syndrome characterized by complex symptoms, difficulty in diagnosis and lack of detailed clinical description. And it is difficult to distinguish from Alzheimer disease. This study aimed to design a method to measure the actual incidence of hydrocephalus in patients with INPH and to evaluate normal pressure hydrocephalus.

**Methods:** 67 patients with idiopathic normal pressure hydrocephalus (INPH), 32 Alzheimer's disease (AD) patients and 30 healthy control (HC) subjects were included. All subjects underwent 3D T1-weighted MRI. Statistical parametric mapping 12 was used for preprocessing images, statistical analysis, and voxel-based morphometric gray matter (GM) volume, white matter (WM) volume, and cerebrospinal fluid (CSF) volume analysis. Pearson’s correlation analysis and Bonferroni's statistic corrected one-way ANOVA were used to determine the relationship among demographic variables.

**Results:** The INPH patients had higher Evans index (EI) and lower callosal angle (CA) compared to the AD patients and HCs. Compared with the HCs, the AD and INPH patients had significantly less GM and WM. The INPH patients accumulated larger amounts of CSF volume in their brains compared to the other groups. The CSF volume ratio was positively correlated with EI and negatively with CA in all 3 groups.

**Conclusion:** Intracranial CSF ratio was strongly correlated with traditional image biomarker of EI and CA, Idiopathic normal pressure hydrocephalus can be accurately assessed by measuring intracranial CSF, and provide a method to distinguish INPH from AD.

## Boosting phase contrast MRI performance in iNPH diagnostics by means of machine learning

### Aleš Vlasák^1,2^, Václav Gerla^3^, Petr Skalický^1,2^, Arnošt Mládek^2,3^, Vojtěch Sedlák^4^, Jiří Vrána^4^, Helen Whitley^2^, Lenka Lhotská^3,5^, Vladimír Beneš Sr^2^, Vladimír Beneš Jr^1^, Adéla Bubeníková^1,2^, Ondřej Bradáč^1,2^

#### ^1^Department of Neurosurgery, Charles University, Motol University Hospital, Prague, Czech Republic; ^2^Department of Neurosurgery and Neurooncology, Charles University, Military University Hospital, Prague, Czech Republic; ^3^Department of Cognitive Systems and Neurosciences, Czech Technical University, Czech Institute of Informatics, Robotics and Cybernetics, Prague, Czech Republic; ^4^Department of Radiology, Charles University, Military University Hospital, Prague, Czech Republic; ^5^Department of Natural Sciences, Czech Technical University, Faculty of Biomedical Engineering, Prague, Czech Republic

##### **Correspondence:** Ondřej Bradáč (ondrej.bradac@uvn.cz)

*Fluids and Barriers of the CNS* 2022, **19(1)**

**Introduction:** Phase contrast MRI allows detailed measurement of various CSF motion parameters. Machine learning (ML) approaches have been successfully utilized in medical research, but none was yet applied to enhance the results of CSF flowmetry. We aimed to evaluate the possible contribution of ML algorithms in enhancing utilization and results of MRI flowmetry in NPH diagnostics.

**Methods:** The study cohort consists of 30 iNPH patients and 15 healthy controls examined on one MRI machine. All major phase contrast parameters were inspected: peak positive and negative velocity, peak amplitude, average velocity, positive, negative and average flow and aqueductal area. We applied ML algorithms on 85 complex features calculated from phase contrast study.

**Results:** The most distinctive parameters with p˂0.005 were peak negative velocity, peak amplitude and negative flow. From the machine learning algorithms, the Adaptive Boosting classifier showed the highest specificity and best discrimination potential overall: 80.4 ± 2.9% accuracy, 72.0 ± 5.6% sensitivity, 84.7 ± 3.8% specificity (0.812 ± 0.047 AUC). The highest sensitivity was 85.7 ± 5.6% by Gaussian Naive Bayes model, the best AUC was 0.854 ± 0.028 by Extra Trees classifier.

**Conclusions:** Developed feature extraction algorithms combined with machine learning approaches simplify the utilization of phase contrast MRI. The highest performing ML algorithm was Adaptive Boosting, which showed a good calibration and discrimination on the testing data, with 80.4% accuracy, 72.0% sensitivity, 84.7% specificity (0.812 AUC). Phase contrast MRI boosted by machine learning approach can help to determine shunt-responsive iNPH patients.

## Use of polyvinylpyrrolidone-coated shunt tubing in the reduction of long-term tubing calcification

### Michael G. Muhonen^1^, Michael Vinzani^3^, Leandro Castañeyra-Ruiz^2^, Alvin Y. Chan^1^ and Seunghyun Lee^2^

#### ^1^Neurosurgery Department at CHOC Children’s Hospital, 505 S Main St. Orange, CA 92868, United States; ^2^CHOC Children’s Research Institute 1201W. LaVeta Avenue Orange, CA 92868, United States; ^3^Medical University of South Carolina, 171 Ashley Avenue, Charleston, SC 29425, United States

##### **Correspondence:** Leandro Castañeyra-Ruiz (Leandro.Castaneyra.Ruiz@Choc.org)

*Fluids and Barriers of the CNS* 2022, **19(1)**

**Keywords**: Polyvinylpyrrolidone, Catheter, Flow/pressure performance, Astrocytes, Choroid plexus, Hydrocephalus

**Introduction:** Approximately 85% of ventriculoperitoneal shunts (VPS) fail within 15 years, with most late failures attributed to calcification of the tubing. It was suggested that application of the hydrophobic molecule, polyvinylpyrrolidone (PVP) to the surface of shunt tubing would prevent infection. While this was never proven, we serendipitously noticed that explanted distal catheters with PVP coating had minimal visible calcification. We hypothesized that PVP coating may decrease the degree of tubing calcification. We retrospectively compared the calcium concentration on PVP-coated VPS catheters versus standard VPS catheters.

**Methods:** 29 shunts that were in place for 13–137 months were explanted. The calcium concentration of PVP-coated (BioGlide) catheters was compared to that of other VPS catheters using inductively coupled plasma atomic emission spectroscopy (IED-AES). To account for varying catheter lengths and implantation times, calcium concentration per cm per month was analyzed.

**Results:** PVP-coated catheters were implanted for 41 months on average while standard catheters were implanted for 67.57 months. Mean calcium concentration was 19.23 for PVP-coated and 209.07 mg/L for standard catheters. Calcium content per catheter length and implantation time was 0.141 and 1.098 ug/cm/month for PVP-coated and standard catheters respectively.

**Conclusion:** In the United States, there are more pediatric hydrocephalus patients admitted each year for VPS malfunctions than for initial shunt placements. Calcification of catheter tubing is a major contributor to late shunt failure, leading to complications such as fractures, disconnections, and tethering. PVP-coated catheters significantly decrease the degree of calcification and may decrease the rate of late shunt malfunction.

## Establishing ranked priorities for future hydrocephalus research

### Mark Hamilton^8^, Noriana E. Jakopin^1,2^, Elliot Myong^1,3^, Trish Bogucki^1^, Diana Gray^1^, Paul Gross^1,4^, J. Gordon McComb^3^, Chevis Shannon^5^, Mandeep S. Tamber^6^, Maiko Toyama^7^, Tessa van der Willigen^1^, Amir Yazdani^1^, Jenna E. Koschnitzky^1^

#### ^1^Hydrocephalus Association, Bethesda, MD, 20814, USA; ^2^University of Maryland, College Park, MD, 20740, USA; ^3^University of Southern California, Los Angeles, CA, 90007, USA; ^4^Cerebral Palsy Research Network, Greenville, SC, 29604, USA; ^5^Department of Neurosurgery, University of Alabama at Birmingham, Birmingham, AL, 35294, USA; ^6^University of British Columbia, Vancouver, BC V6T 1Z4, Canada; ^7^Edwards Lifesciences, Irvine, CA, 92614, USA; ^8^Department of Clinical Neurosciences, University of Calgary, Calgary, AB, Canada, T2N 2T9

##### **Correspondence:** Mark Hamilton (mghamilton.hydro@gmail.com)

*Fluids and Barriers of the CNS* 2022, **19(1)**

**Introduction:** The aim of this initiative was to develop a ranked list of hydrocephalus research priorities as determined by the hydrocephalus patient community in conjunction with the healthcare and scientific communities.

**Methods:** Using the validated methodology published by the James Lind Alliance (JLA), the Hydrocephalus Association (HA) administered two surveys and hosted a final prioritization workshop. Survey_One solicited open-ended responses from the community. From these responses, a “long list” of priority statements was developed. This list was then consolidated into a “shortlist” of research priority statements, which were verified as being research uncertainties through a literature review. Survey_Two asked the community members to select their top 10 priorities from the “shortlist.” The final prioritization took place at a virtual workshop led by a team of trained facilitators.

**Results:** From Survey_One, 3703 responses from 890 respondents were collected, leading to a “long list” of 146 priority statements. The consolidated “shortlist” contained 49 research priority statements, all of which were verified as uncertainties in hydrocephalus research. From an analysis of Survey_Two responses, the top 21 research priority statements were determined. A consensus on these statements was reached at the virtual workshop, leading to a final ranked Top 20 list of hydrocephalus research priorities. Within this list several themes are apparent. These include the need to develop non-invasive and/or one-time therapies, to reduce the burden of current treatments, improve the screening and diagnosis of hydrocephalus, improve quality of life, and improve access to care.

**Conclusions**: By gathering extensive input from the hydrocephalus community and using an iterative process of consensus building, a ranked list of the Top 20 hydrocephalus research priorities was developed. The Hydrocephalus Association will use this ranked list to guide future research programs and encourages the medical and scientific communities to do the same.

## Diagnostic value of cerebrospinal fluid production rate in normal pressure hydrocephalus

### Kanza Tariq^1^, Ahmed Toma^1^, Mohamed A. Elborady^1^, Lewis Thorne^1^, Laurence Watkins^1^

#### ^1^National Hospital for Neurology and Neurosurgery, Queen Square, London, UK

##### **Correspondence:** Kanza Tariq (kanza.tariq@nhs.net)

*Fluids and Barriers of the CNS* 2022, **19(1)**

**Introduction:** Predicting shunt responsiveness in the context of normal-pressure hydrocephalus(NPH) can be challenging. We aimed to study the correlation between cerebrospinal fluid production rate (PRcsf) and lumbar drainage responsiveness of suspected NPH patients.

**Methods:** We performed a prospective observational study in all suspected NPH patients in our hospital who required extended lumbar drainage (ELD). Following baseline cognitive assessment and walking test, ELD was undertaken. The lumbar drain was connected to LiquoGuard7(Möller-Medical, Germany). The LiquoGuard7 was used to calculate PRcsf in the patients using the internal software and flow-rate data of the pump. Cognitive assessment and walking test were performed post-ELD. Patients were followed up for 6 months post hospital discharge. Electronic patient records and clinic letters were studied to identify ELD responsive and non-responsive patients.

Statistical analysis used SPSS (version 25.0, IBM) by independent t-test, comparing measured PRcsf to the ELD responsiveness of the patient.

**Results:** 20 suspected NPH patients were evaluated. 16 NPH patients demonstrated a mean PRcsf of 79 ml/hour ± 20(SD). These patients showed objective improvement in post-ELD cognitive assessment and walking test (p < 0.0001). At a 6 month follow up, all 16 patients showed positive response to shunting(p < 0.0001). Four suspected NPH patients had an average PRcsf of 22-32 ml/hour. These patients did not show any objective improvement in post-ELD cognitive assessment and walking test.

**Conclusion:** PRcsf of > twice the normal may hold value in predicting shunt responsiveness in the context of NPH. Along with ELD, this has the potential of being an additional diagnostic tool for NPH.

## Cerebral spinal fluid biomarkers for diagnosis, surgical outcome prediction and stratification of idiopathic normal pressure hydrocephalus affected patients: a systematic review

### G. Grasso^1^, F. Torregrossa^1^

#### ^1^Neurosurgical Unit, Department of Biomedicine, Neurosciences and Advanced Diagnostics (BiND), University of Palermo, Italy

##### **Correspondence:** Giovanni Grasso (giovanni.grasso@unipa.it)

*Fluids and Barriers of the CNS* 2022, **19(1)**

**Introduction:** Idiopathic normal pressure hydrocephalus (iNPH) is a possible differential diagnosis of dementia in elderly patients. Its treatment is based on cerebral spinal fluid (CSF) shunt with 60%-80% patients’ improvement. The prevalence of iNPH was estimated at 0.2% in the age group of 70 to 79 years and 5.9% for age 80 years and older. However, only a few cases are operated on every year. Diagnosis of iNPH requires the exclusion of other neurological pathologies such as Alzheimer’s Disease or other neurodegenerative disorders presenting with ventricular enlargement.

**Methods:** The authors conducted a systematic review following PRISMA guidelines on the PubMed database. 982 articles were identified for screening. Studies analyzing CSF biomarkers associated with diagnosis, surgical outcome prediction, and stratification of iNPH patients were included. Studies were assessed for methodological quality using the ROBIS tool.

**Results:** Eighteen studies were eligible for our systematic review. Although many biomarkers have been investigated for differential diagnosis of iNPH, amyloid-b 42 (Ab42), total-tau (t-tau), and phosphorylated tau (p-tau) are the most efficient candidate markers to discriminate iNPH from AD patients. Additional biomarkers categorized as demyelination, neuroinflammation, neuropeptides, and cerebral metabolites hold promise to better clarify innovative diagnostic biomarkers for iNPH.

**Conclusions:** Measurements of different biomarkers in CSF may reflect the underlying neuropathological brain changes and could play an important role in revealing the possible etiological mechanisms. Furthermore, its detection may facilitate the timeliness and accuracy of iNPH diagnosis and thus potentially useful for therapeutic selection and treatment response monitoring.

## Radiological evaluation versus infusion test in hydrocephalus patients

### Serge Metanbou^1,2^, Cyrille Capel^1,3^, Kimi Owashi^1^, Vakaramoko Kone^1^, Zofia Czosnyka^4^, Marek Czosnyka^4^, Peter Smielewski^4^, Olivier Balédent^1,5^

#### ^1^Chimère UR 7516, Jules Verne University, Amiens, France; ^2^Radiology, Jules Verne University hospital, Amiens, France; ^3^Neurosurgery, Jules Verne University hospital, Amiens, France; ^4^Department of Clinical Neurosciences, University of Cambridge, Cambridge, UK; ^5^Image processing, Jules Verne University hospital, Amiens, France

##### **Correspondence:** Olivier Balédent (olivier.baledent@chu-amiens.fr)

*Fluids and Barriers of the CNS* 2022, **19(1)**

**Introduction:** Disproportionately Enlarged Subarachnoid-space hydrocephalus (DESH) is one of image-based score proposed to the neuroradiologist to easily evaluate normal pressure hydrocephalus (NPH) patients. Resistance to CSF outflow (R_**out**_) a pressure-related parameter obtained with infusion test is actually one of the most sensitive and specific parameters to select NPH patients for shunt surgery with a chance to improve_**.**_ The aim of this work was to compare radiological investigation with infusion test parameters.

**Methods:** 28 patients (74 ± 9 years) with suspected NPH on clinical symptoms underwent first a 3 T MRI with standard morphological sequences used to evaluate brain tissues and CSF compartments. Radiologist calculated DESH scores (from 1 to 10) based on the following 5 evaluations (scored from 0 to 2): ventriculomegaly, dilated sylvian fissures, tight high convexity, acute callosal angle, and focal sulcal dilation. In a second step independently of the DESH score, patients had a continuous infusion test and the neurosurgeon calculated Rout, elastance and mean ICP using ICM + software.

**Results:** DESH score showed a slight correlation with Rout (R2 = 0.33, p > 0.005 (p = 0.01)) a poor one with elastance (R2 = 0.13, p > 0.05 (p = 0.39)) and none with mean ICP (R2 = 0.02, p > 0.5 (p = 0.73)).

**Conclusions:** A reproducible method must be applied to calculate DESH score, especially the callosal angle, to avoid false evaluation. A history of the symptoms should be taken into account. Suspected NPH Patients that present with DESH negative results should not be excluded from potentially becoming shunt responders based on this test alone.

**Study supported by**: Revert Project, Interreg, France (Channel Manche) England, funded by European Regional Development Fund.

## Involvement of Aquaporin-1 in brain water homeostasis

### Dennis B. Jensen^1^, Dagne Barbuskaite^1^ and Nanna MacAulay^1^

#### ^1^Department of Neuroscience, University of Copenhagen, Denmark

##### **Correspondence:** Nanna MacAulay (macaulay@sund.ku.dk)

*Fluids and Barriers of the CNS* 2022, **19(1)**

**Introduction:** The majority of cerebrospinal fluid (CSF) is produced by the choroid plexus, a highly specialized tissue consisting of an epithelial monolayer surrounding fenestrated capillaries. The choroid plexus epithelial cells express a multitude of membrane transporters and channels, and their transepithelial ion movement has been proposed to drive CSF secretion by osmotically obliged water movement through aquaporin-1 (AQP1) localized to the luminal membrane. However, CSF production occurs in the absence of a transchoroidal osmotic gradient, and can even take place against an experimentally-induced unfavorable osmotic gradient, questioning the exact role of AQP1 in CSF production. This ongoing project aims to determine the contribution of AQP1 in CSF secretion and brain water homeostasis by using mouse models deficient in AQP1.

**Methods:** MRI were performed on wild type (WT) and global AQP1^−/−^ mice to study the size of the lateral ventricles. Additionally, the total brain water content was determined by dehydrating the brains.

**Results:** The initial study with the AQP1^−/−^ mouse model revealed brain water content and lateral ventricle size similar to those of WT mice.

**Conclusion:** These results indicate that AQP1 may play a minor role in brain water homeostasis. Future studies will determine the intracranial pressure and rate of CSF production in the two strains, which are aimed to extend into differentiation between a direct effect of AQP1 deficiency in choroid plexus versus the indirect effects on brain water homeostasis anticipated to arise following the altered systemic water homeostasis and lower central venous blood pressure in the global AQP1^−/−^ mouse.

## Recovery from the impact of MRI protocol changes on machine learning methods for MRI-based DESH detection

### Jeffrey L. Gunter^1^, Petrice M. Cogswell^1^, Matthew L. Senjem^1^, Kejal Kantarci^1^, David S. Knopman^1^, Ronald C. Petersen^1^, Benjamin D. Elder^1^, Neill R. Graff-Radford^2^, Jonathan Graff-Radford^1^, Clifford R. Jack Jr^1^

#### ^1^Mayo Clinic, Rochester, MN, 55905, USA; ^2^Mayo Clinic, Jacksonville, FL, 32224, USA

##### **Correspondence:** Jeffrey Gunter (gunter.jeffrey@mayo.edu)

*Fluids and Barriers of the CNS* 2022, **19(1)**

**Introduction:** DESH-like spatial patterns in CSF spaces maybe detected using a machine learning method (denoted as CDESH) in roughly 6% of elderly individuals in the Mayo Clinic Study of Aging (MCSA). We have shown stable algorithm performance over long longitudinal series acquired with consistent MRI protocols. In 2017 the MCSA MRI protocol was modernized, introducing a discontinuity that impaired CDESH performance.

**Methods:** T1-weighted structural MRI imaging in MCSA was performed from 2004 through 2017 at 1.2. × 1.05 × 1.05 mm spatial resolution. Since 2017 scanning has been done at 0.8 mm isotropic spatial resolution. The CDESH method includes SPM image segmentation, parcellation of CSF spaces into atlas regions, and the use of a support vector machine to combine regional CSF volumes trained to detect the DESH imaging features. The support vector machine was trained on the older images. 113 people were imaged near-in-time on both protocols, providing a direct crossover data set. Relative to CDESH scores from the older protocol scans we compared CDESH scores from new protocol scans at 0.8 mm isotropic resolution and new protocol scans resampled to match old protocol spatial resolution.

**Results:** Correlation between CDESH scores on images at 0.8 mm resolution and scores from older scans was poor (r < 0). Agreement between scores on resampled new scans and older scans was very strong (r =  + 0.83).

**Conclusion:** The CDESH method is sensitive to MRI spatial resolution. Resampling images to match the spatial resolution of images used to train the algorithm ameliorates this sensitivity, allowing the seamless use of MCSA images spanning over 18 + years.

## Diagnostic accuracy of thermal transcutaneous flow compared with radionuclide shunt patency study to detect shunt obstruction in adults with normal pressure hydrocephalus-a cross sectional analytic study

### Naomi Abel^1^

#### ^1^Department of Neurosurgery, University of South Florida, Tampa, Florida, 33707, USA

##### **Correspondence:** Naomi A Abel (nabel@usf.edu)

*Fluids and Barriers of the CNS* 2022, **19(1)**

**Introduction:** Increased recognition of communicating hydrocephalus in adults in an aging population and technological improvements in ventriculoperitoneal shunts (VPS) specifically programmable valves has resulted in an increase in diagnosis and treatment. Symptoms suggestive of shunt malfunction are common and may be subtle. Evaluation for shunt patency often involves invasive procedures including radionuclide shunt patency study (SPS) or contrast studies. Thermal transcutaneous flow (TTF) is a noninvasive tool to evaluate cerebrospinal fluid (CSF) flow in VPS. The diagnostic accuracy of TTF to detect shunt patency was assessed.

**Methods:** All consecutive patients with communicating hydrocephalus and VPS who initially demonstrated improved gait velocity followed by decline at a single center were eligible for inclusion and underwent TTF tests in the sitting position before and after SPS (reference standard).

**Results:** As of April 2022, 8 patients met eligibility and demonstrated patency by SPS. TTF confirmed flow in 6 of the 8 patients resulting in a specificity of 75% and negative predictive value of 100%**.**

**Conclusions:** Preliminary results show that TTF may be a helpful noninvasive tool to screen patients prior to consideration for invasive studies to evaluate VPS patency. The final result after complete accrual is awaited.

## 3-Dimensional volumetric segmentation of the optic nerve sheath and hypophysis in patients with raised intracranial pressure

### Musa China^1^, Anand S. Pandit^2^, Linda D’Antona^2^, Ahmed K. Toma^2^

#### ^1^University College London, UCL, Division of Medicine, London, United Kingdom; ^2^National Hospital for Neurology and Neurosurgery, Victor Horsley Department of Neurosurgery, London, United Kingdom

##### **Correspondence:** Musa China (musa.china.15@ucl.ac.uk)

*Fluids and Barriers of the CNS* 2022, **19(1)**

**Introduction:** Intracranial pressure (ICP) typically requires invasive monitoring for accurate measurement. The utility of non-invasive MRI biomarkers, including enlarged optic nerve sheath (ONS) diameter and hypophysis compression are unclear, and likely to be inaccurate when viewed in 2-dimensions. We aimed to evaluate 3-dimensional MRI-based segmentations of the ONS and hypophysis as objective markers, assessing their relationship with 24-h ICP readings.

**Methods:** A single-centre retrospective study included patients who underwent high-resolution isotropic T2w MRI within three months of 24-h ICP monitoring. Semi-automated segmentations of the ONS and hypophysis were performed with inter-subject intracranial volume normalisation. Volumetric measurements were correlated with CSF pressures.

**Results:** 25 patients (mean age: 44-years, females: 14) were included. 20 patients had raised ICP in the sample, of these 14 underwent CSF diversion procedures. Left ONS and hypophysis volume were both significantly correlated with 24-h ICP readings (Pearson correlation coefficients: 0.45 and 0.60, p = 0.049 and 0.02). Mean values of left ONS volume (752 ± 222 mm^3^) were significantly higher in patients with raised ICP versus normal (523 ± 177 mm^3^), (p = 0.04) and higher left ONS volume was significantly associated with patients undergoing CSF diversion (p = 0.02). No significant difference was found between right ONS volume and raised or normal ICP patients, however, right ONS volume was significantly associated with left ONS volume and future CSF diversion (p = 0.05).

**Conclusions:** MR-based volumetric segmentations, including unilateral ONS enlargement, are significantly associated with raised ICP and the need for intervention. Further work seeks to expand on this sample and compare against other non-invasive imaging biomarkers.

## The development and testing of an efficient benchtop testbed for the quantitative analysis of gross obstruction in ventricular catheters

### Carolyn A. Harris^1^, Pranav Gopalakrishnan^2^, Ahmad Faryami^3^

#### ^1^Department of Chemical Engineering and Materials Science, Wayne State University, Detroit, MI, 48202, USA; ^2^Department of Medical Education, Wayne State University School of Medicine, Detroit, MI,48202, USA; ^3^Department of Biomedical Engineering, Wayne State University, Detroit, MI, 48202, USA

##### **Correspondence:** Carolyn A. Harris (caharris@wayne.edu)

*Fluids and Barriers of the CNS* 2022, **19(1)**

**Introduction:** Despite the prevalence of shunt obstruction and the high rate of shunt revisions, there are a limited number of methods that improve our understanding of the overall degree of obstruction without exhaustive, high-end histological analysis.

**Methods:** The current study develops an inexpensive, easy to use gravity-driven model that measures flow through naïve and explanted ventricular catheters from patients. The purpose of this model is to provide a quick, simple analysis of the resistance to CSF flow through the ventricular catheter caused by tissue obstruction. Catheters of several manufacturers were tested. Flow and pressure data were measured using appropriate sensors; resistance to outflow of different catheter designs were evaluated. A subset of experiments measured changes in relative resistance when catheter hole interfaces were progressively and systematically blocked. Relative resistance of explanted catheters from our clinical shunt biobank was also measured.

**Results:** The model was built and tested that provides data on overall obstruction of ventricular catheters, of which can be sent wirelessly to researchers worldwide when appropriate. Experimental results from our gross analysis of resistance showed that there are significant differences between the relative resistances of different catheter models without obstruction at all. In samples explanted from patients, we see varying degrees of resistance to CSF flow through the system, indicating the potential for our testbed to quantitatively differentiate bulk tissue obstruction with high throughput.

**Conclusions:** The current study is intended both to validate the proposed model and to examine data on differences in relative resistance between catheter models.

## A high-throughput assay for screening material-mediated cellular adhesion for neurological implants

### Tanzil Islam^1^, Heli Modi^1^, Amulya Veldanda^1^, Victoria Carr^1^

#### ^1^Integra LifeSciences, Princeton, NJ, 08540, USA

##### **Correspondence:** Tanzil Islam (tanzil.islam@integralife.com)

*Fluids and Barriers of the CNS* 2022, **19(1)**

**Introduction:** Current in-vitro models for cellular adhesions in shunts have limited scalability due to model complexities. An assay capable of large concurrent sample sizes is proposed for assessing material-mediated neuroglial cellular adhesion to shunt catheter materials.

**Methods:** Astrocyte adhesion to three Shore 50A platinum cured silicone tubes (A, B and C) were evaluated. The assay consists of 5 mm long tubes with transverse fenestrations in 1 mL of media in conical vials. Each sample is seeded with 250,000 astrocytes/ml and incubated on an orbital shaker with continuous agitation to provide fluidic shear to stimulate astrocyte adhesion. The assay can accommodate up to 30 samples concurrently and run for multiple weeks. For comparison, astrocyte adhesion to the same materials were also evaluated via bioreactors with recirculating media at a more physiologically relevant flowrate of 20 ml/hr. All samples were cut in half longitudinally, fixed, stained, and imaged via fluorescent confocal microscopy to quantify percent surface coverage.

**Results:** Initial evaluations of silicone materials A, B and C exhibited differences in percent astrocyte surface coverage even though the materials were nominally equivalent. These results agree with surface coverage trends observed in the more complex bioreactor studies.

**Conclusions:** The assay offers an efficient, yet simple, method to assess material-mediated cellular adhesions with statistically significant samples sizes. Despite not utilizing physiologically relevant CSF dynamics, the mechanical agitation is able to stimulate neuroglial cell adhesion. This assay could serve as a screener for assessing material-mediated cellular adhesions in shunt and other neurological applications.

## Finite state machine for position dependent hydrocephalus shunt therapy

### David Iselin^1^, Joris Chomarat^1^, Caroline Holzer^1^, Janina Hug^1^, Tiago Hungerland^1^, Luca Krebs^1^, Rosina Weiss^1^, Fabian Flürenbrock^2^, Leonie Korn^2^, Anthony Podgoršak^1^, Dominik Schulte^1^, Nikolaos Tachatos^1^, Markus Florian Oertel^3^, Lennart Stieglitz^3^, Mirko Meboldt^1^, Melanie Zeilinger^2^ and Marianne Schmid Daners^1^

#### ^1^Product Development Group Zurich, ETH Zurich, Switzerland; ^2^Institute for Dynamic Systems and Control, ETH Zurich, Switzerland; ^3^Department of Neurosurgery, University Hospital Zurich, Switzerland

##### **Correspondence:** David Iselin (iselind@ethz.ch)

*Fluids and Barriers of the CNS* 2022, **19(1)**

**introduction:** Intracranial pressure (ICP) being strongly position-dependent complicates pressure-based shunt control as a reliable therapy approach for hydrocephalus patients. An algorithm using state detection and position-specific ICP reference selection implemented on a novel mechatronic shunt platform enables regulation towards a physiological ICP in any patient’s posture.

**Methods:** A finite state machine (FSM) was developed. Two states represent pressure-control for positions of known reference pressures, namely supine and upright. A third state represents flow-control for positions with unknown reference pressures and positional changes. The FSM switches cases by tracking the torso inclination of the patient every second, using an increasing counter when leaving a certain case-dependent angular interval and changing case when reaching a predefined counter threshold.

**Results:** During hardware-in-the-loop tests, position-dependent pressure references of physiological ICP could be tracked without offset during sequences of postural changes. The case-dependent thresholds improved robustness against sensor noise and patient motion. Increasing threshold values for changes from flow to pressure-based cases and decreasing values vice versa improved the controller stability. Flow-based regulation using averaged empirical flows as reference allowed for physiological cerebrospinal fluid drainage even during intervals of severe ICP fluctuations and disturbances.

**Conclusions:** FSM posture detection proves to be an efficient extension to pressure-based shunt control. The implementation of case-dependent threshold values allows for a trade-off between fast and robust system response and will be a central aspect of future research. Possibilities arising from the FSM enable the extension to further position and activity states, rendering the controller personalised for every patient.

## Radiological signs of iNPH without clinical symptoms-a longitudinal study

### Karin Kockum^1^, Johanna Andersson^1^, Otto Lilja-Lund^1^, Katarina Laurell^2^

#### ^1^Department of Clinical Science, Neurosciences, Umeå university, Östersund, Sweden; ^2^Department of Neuroscience, Neurology, Uppsala University Hospital, Uppsala, Sweden

##### **Correspondence:** Karin Kockum (karin.kockum@regionjh.se)

*Fluids and Barriers of the CNS* 2022, **19(1)**

**Introduction:** Asymptomatic ventriculomegaly with iNPH features in MRI (AVIM) was first described by Iseki et al. in 2009. A later study from Japan, reported that about half of the patients with AVIM develop symptoms after three years (Kimihira et al. 2020). We aimed to see if we could confirm this finding in a Swedish population-based material of AVIM.

**Methods:** In a prospective prevalence study, conducted between 2014 and 2017, previously described by our group, 122 individuals over 65 completed follow-up with neurological examination and computed tomography of the brain. The iNPH Radscale (Kockum et al., 2018) and the Hellström iNPH scale (Hellstrom et al., 2012) were used to assess the degree of radiological signs and triad symptoms, respectively.

**Results:** Four individuals (3.2%) had radiological signs of iNPH (iNPH Radscale ≥ 5) with no or few symptoms (Hellström iNPH scale ≥ 80). At follow-up, two years later, three of the participants had increased in radiological scoring, whereas the NPH scale of symptoms remained above 80 points. Five years later, two of the three individuals had further deteriorated in radiological scoring and also presented the whole triad of iNPH symptoms.

**Conclusions:** Our results confirm previous findings that radiological signs of iNPH precede symptom development within a few years in about half of the cases. Therefore, yearly clinical follow-up is suggested for patients with radiological incidental findings of iNPH, to facilitate early detection and treatment.

## Intracranial pressure characteristics in patients with idiopathic intracranial hypertension after shunt implantation

### Mahmoud Samara^1^, Kyaw Zayar Thant^1^, Sebastian Yim^2^, Marian E Byrne^1^, Alexandra J Sinclair^3,4^, Susan P Mollan^3,5^, Georgios Tsermoulas^1,3^

#### ^1^Department of Neurosurgery, Queen Elizabeth Hospital Birmingham, United Kingdom; ^2^University of Birmingham Medical School, United Kingdom; ^3^Institute of Metabolism and Systems Research, University of Birmingham, United Kingdom; ^4^Department of Neurology, Queen Elizabeth Hospital Birmingham, United Kingdom; ^5^Birmingham Neuro-Ophthalmology, Queen Elizabeth Hospital Birmingham, United Kingdom

##### **Correspondence:** Mahmoud Samara (Mahmoud.Samara@nhs.net)

*Fluids and Barriers of the CNS* 2022, **19(1)**

**Introduction:** Patients with idiopathic intracranial hypertension (IIH) and vision threatening papilloedema require cerebrospinal fluid (CSF) diversion. We have developed a surgical protocol for CSF shunting in these patients that includes the integration of the M.scio telemetric sensor (Meithke, Potsdam, Germany). This observational study describes the intracranial pressure (ICP) characteristics of shunted patients with IIH.

**Methods:** Sixty-two consecutive IIH patients underwent ventriculoperitoneal shunt insertion from July 2019 to February 2022 and we have available telemetric ICP recordings for 53. The first telemetric ICP recording after shunt implantation was assessed, in order to analyse the main parameters of the ICP waveform. Recordings from malfunctioning shunts were included following revision.

**Results:** The ICP waveforms demonstrated arterial pulsatility in 45 (85%) patients. The lack of pulsatility did not indicate shunt malfunction in the remaining patients. In sitting position, the mean ICP was -5cmH_2_O (range -26 to 13) and the amplitude was 8cmH_2_O. In supine position, the mean ICP was 23cmH_2_O (-1 to 43) and the amplitude was 7cmH_2_O. The mean difference in ICP between sitting and supine positions was 28 cm H_2_O.

**Conclusions:** The results provide a normal range of values for ICP recordings via a telemetric sensor in shunted patients with IIH. They will assist in the interpretation of ICP data in cases of shunt malfunction and may advise in the optimal valve settings. This is the first study to provide insight into the ICP characteristics in IIH patients with functioning shunts in order to inform decision making.

## Correlation between cerebrospinal fluid opening pressure and body fat distribution in idiopathic intracranial hypertension (IIH) patients

### Sara Ho^1^, Yifan Zhang^2^, Abhay Moghekar^1^

#### ^1^Department of Neurology, Johns Hopkins University, Baltimore, MD, 21224, USA; ^2^Department of Biostatistics, Johns Hopkins University, Baltimore, MD, 21287, USA

##### **Correspondence:** Abhay Moghekar (am@jhmi.edu)

*Fluids and Barriers of the CNS* 2022, **19(1)**

**Introduction:** Although idiopathic intracranial hypertension is strongly associated with obesity, the relationship between body fat distribution and IIH has yet to be fully characterized. Pilot studies have shown that central obesity is a risk factor for IIH. This study aimed to examine parameters of body composition in IIH patients and determine if body fat distribution correlates with clinical measures of IIH.

**Methods:** Data was collected from 360 patients seen at the Johns Hopkins Center for CSF Disorders. Body composition parameters including visceral adipose tissue (VAT), body mass index (BMI), fat mass (FM), extracellular water (ECW), and total body water (TBW) were measured using bioelectrical impedance analysis (Seca, medical Body Composition Analyzer 515; seca mBCA 515). Lumbar puncture manometry was used to measure cerebrospinal fluid opening pressure in the left lateral decubitus position. Visual acuity was assessed using LogMar charts. Color vision was assessed using HRR plates. Spearman’s correlation was used to determine the correlation between opening pressure and body composition parameters.

**Results:** 333 female patients and 27 male patients were assessed (mean age 39.41 ± 13.17 years, range 13–80 years). Mean BMI across patients was 34.87 ± 14.49 and mean FM was 92.27 ± 0.39.22 kgs. BMI and FM demonstrated moderate correlations with intracranial pressure (r = 0.31, p < 0.001; r = 0.31, p < 0.001 respectively). VAT, ECW, and TBW showed weaker correlations with intracranial pressure (r = 0.16, p = 0.0027; r = 0.19, p = 0.00024; r = 0.17, p = 0.0014 respectively). There were no correlations of VAT with visual acuity and color vision (r = 0.059, p = 0.27; r = -0.074, p = 0.17 respectively).

**Conclusion:** In IIH patients, measures of obesity namely body mass index and fat mass correlate with intracranial pressure better than visceral adiposity and other body composition parameters. Body fat distribution does not contribute to additional risk as compared to standard measures.

## Obesity in female rats does not cause idiopathic intracranial hypertension –are secondary factors required?

### J. H. Wardman^1^, S. N. Andreassen^1^, M. N. Jensen^1^, B. Styrishave^2^, A. B. Sinclair^3^, N. MacAulay^1^

#### ^1^Department of Neuroscience and ^2^Department of Pharmacy, University of Copenhagen, Copenhagen, 2200, Denmark; ^3^Institute of Metabolism and Systems Research, University of Birmingham, Birmingham, B15 2SQ,United Kingdom

##### **Correspondence:** Jonathan Wardman jonathan.wardman@sund.ku.dk

*Fluids and Barriers of the CNS* 2022, **19(1)**

**Introduction:** Idiopathic Intracranial Hypertension (IIH) is a condition characterized by increased intracranial pressure (ICP), impaired vision and headache, but with unresolved etiology. IIH occurs predominantly in obese (body mass index (BMI) ≥ 30) women of childbearing age, though age, BMI and female sex do not encompass all aspects of IIH pathophysiology. Female IIH patients show a distinct hormonal profile, highlighted by androgen excess. To reveal the etiology of IIH, we modelled female obesity in rats.

**Methods:** Zucker rats genetically lacking the leptin receptor, were grown to obesity and their ICP, brain water content, ventricular morphology, cerebrospinal fluid (CSF) production, and choroidal transcriptomic profile assessed in comparison to their lean counterparts. The IIH-related androgen excess was mimicked by bi-weekly testosterone injections (4 weeks) of female Wistar rats followed by determination of their brain water content, CSF production, and choroidal transport activity.

**Results:** Obesity alone did not create the elevated ICP characteristic of IIH, nor did it alter various aspects of brain fluid dynamics or the choroidal transportome. Testosterone treatment, on the other hand, caused elevated CSF production due to increased activity of the NKCC1 transport protein.

**Conclusions:** These data indicate that while obesity and female sex are characteristic of IIH, these physiological aspects do not alone establish IIH pathophysiology, and that the IIH-related androgen excess may be crucial in IIH etiology. Future studies establishing an IIH animal model might require the combination of these, and possibly other, features.

## Improving diagnostic accuracy with ICP monitoring in patients with ambiguous pressure symptomatology

### Michael Meggyesy^1^, Gwendolyn Williams^1^, Enoch Kim^2^, Richard Um^1^, Dipankar Biswas^1^, Aruna Rao^1^, Mark G. Luciano^1^

#### ^1^Department of Neurosurgery, Johns Hopkins University School of Medicine, Baltimore, MD, 21205, USA; ^2^Nova Southeastern University Dr. Kiran C. Patel College of Allopathic Medicine, Fort Lauderdale, FL, 33314, USA

##### **Correspondence:** Michael Meggyesy (mmeggye1@jhmi.edu)

*Fluids and Barriers of the CNS* 2022, **19(1)**

**Introduction:** Diagnosis and treatment in potential hydrocephalus patients with ambiguous symptomatology is a challenge. Invasive ICP monitoring (ICPm), the gold standard in diagnostics, can be used to solve difficult cases with long term pressure readings. In this retrospective study, our aim was to determine if ICP monitoring can improve accuracy in diagnosis and prevent further unnecessary invasive treatment in patients with ambiguous clinical presentations.

**Methods:** Single center retrospective chart review of 268 patients who underwent ICP monitoring, grouped by previously known hydrocephalus related diagnosis and reported clinical symptoms, at the Johns Hopkins Cerebral Spinal Fluid Center within the departments of Neurosurgery and Neurology, between June 2016 and June 2021.

**Results:** Of the 268 patients analyzed here, 121 patients were suspected to be hypotensive, 114 were suspected to be hypertensive, and 33 had a mixed diagnosis. 46% of suspected hypotensive patients and 58% of suspected hypertensive patients were found to have normal ICPs after monitoring.

**Table:** 

ICP results	Suspected hypotensive (n = 121)	Suspected hypertensive (n = 114)
Normal ICP	56 (46%)	66 (58%)
Hypotensive	59 (49%)	17 (15%)
Hypertensive	4 (3%)	30 (26%)
Inconclusive	2 (2%)	1 (< 1%)

**Conclusions:** ICP monitoring should be considered in patients with ambiguous presentation to improve diagnostic accuracy and prevent unnecessary surgery.

## Towards an automated identification of typical walking patterns in NPH patients using wearable sensors

### Lennart Henning Stieglitz^1^, Sandra Dias^1^, Christina Graf^2^, Elisabeth Jehli^1^, Markus Florian Oertel^1^, Julia Mahler^1^, Marianne Schmid Daners^2^

#### ^1^Department of Neurosurgery, University Hospital Zurich, Zurich, 8091, Switzerland; ^2^Product Development Group Zurich, ETH Zurich, Zurich, 8092, Switzerland

##### **Correspondence:** Lennart Henning Stieglitz (Lennart.Stieglitz@usz.ch)

*Fluids and Barriers of the CNS* 2022, **19(1)**

**Introduction:** An experienced physician may tell the likelihood of iNPH from the walking pattern alone. However, the challenge is to provide the benefits of a “diagnostic view” to as many patients as possible to improve iNPH identification and treatment rates. We therefore performed a prospective controlled study using wearable inertia measurement units (IMUs) to identify typical walking patterns in preparation of a future automated patient identification system.

**Methods:** Five wearable inertial measurement units were placed in 20 iNPH patients, 20 elderly age- and gender-matched healthy controls (EHCs) and 20 young healthy controls (YHCs) for data collection in their home environment during 72 h, as well as 6 months after shunt implantation in case of the iNPH patients.

**Results:** There was a significant difference for most of the gait parameters when YHC, EHC and iNPH patients were compared. Postoperatively, iNPH patients showed an improvement of the swing phase (p = 0.039). In gait velocity, stance to swing ratio, stance phase, cycle time and cadence, a significant difference was observed for patients with a good VP-shunt response (NPH RR ≥ 5). A receiver operating characteristic analyses showed a good sensitivity for stride length ≥ 0.44 m, gait velocity ≥ 0.39 m/s and cadence ≥ 0.81 steps/min.

**Conclusions:** The walking-pattern characteristics in iNPH patients are far more evident in the home environment than during a physical examination. It can be used to distinguish patients from healthy persons and to predict response to VP shunt therapy.

## Three-dimensional gait analysis with an optical tracking system captures characteristic gait changes in iNPH

### Lena Kollén^1^, Roland Zügner^2,3^, Roy Tranberg^2,3^, Carsten Wikkelsø^1^, Mats Tullberg^1^

#### ^1^Hydrocephalus Research Unit, Institute of Neuroscience and Physiology, Department of Clinical Neuroscience, The Sahlgrenska Academy, University of Gothenburg, Sweden; ^2^Department of Orthopaedics, Institute of Clinical Sciences, Sahlgrenska Academy, University of Gothenburg, Gothenburg, Sweden; ^3^Orthopaedics, Sahlgrenska University Hospital, Gothenburg, Sweden

##### **Correspondence:** Lena Kollén (lena.kollen@vgregion.se)

*Fluids and Barriers of the CNS* 2022, **19(1)**

**Introduction:** Idiopathic normal pressure hydrocephalus (iNPH) is characterised by progressive disturbance of gait and postural function, affecting spatiotemporal gait parameters, kinematics and balance. To improve precision of the diagnostic work-up and optimise treatment of iNPH patients, there is a need for an objective, standardised quantification of the typical gait pattern. We explored gait function in iNPH patients in comparison with a healthy control group using an optical tracking system (OTS).

**Methods:** INPH patients (n = 26, mean age 75.7 range 68–87, 27% females) and age-matched healthy controls (HC) (n = 22, mean age 71.6 years, range 61–82, 55% females) underwent a three-dimensional gait analysis (3DGA) according to a standardized protocol using an OTS. Data of spatiotemporal and hip kinematic variables were compared across groups using non-parametric statistics.

**Results:** All temporal gait parameters such as speed (mean 0.68 ± 0.25 (± SD) vs 1.21 ± 0.22 m/s), cadence (108 ± 15 vs 114 ± 6 steps/min), step width (0.16 ± 0.04 vs 0.09 ± 0.03 m), step length (0.79 ± 0.23 vs 1.26 ± 0.21 m) and stance (67 ± 7 vs 62 ± 3%) differed significantly between iNPH and HC groups respectively (p < 0.013). Both hip extension (-3° vs -12°) and range of hip flexion/extension (31° vs 40°) were significantly lower in iNPH patients (p < 0.001).

**Conclusion:** 3DGA using OTS captures typical features of the iNPH gait, indicating that this method could add additional value to the routine clinical examination in the evaluation of iNPH patients pre- and postoperatively and be used to explore the iNPH gait pattern further.

## Clinical assessment of step-height and width in iNPH using wearable sensors

### Tomas Bäcklund^1^, Johan Eriksson^2^, Nina Sundström^1^

#### ^1^Department of Radiation Sciences, Biomedical Engineering, Umeå University, City, Umeå, 90187, Sweden; ^2^Department of Clinical Science, Umeå University, Umeå, 90187, Sweden

##### **Correspondence:** Tomas Bäcklund (tomas.backlund@regionvasterbotten.se)

*Fluids and Barriers of the CNS* 2022, **19(1)**

**Introduction:** Impaired gait is a common and early symptom in patients with idiopathic normal pressure hydrocephalus (iNPH). The typical gait pattern is broad-based, shuffling and short-stepped. Clinically, gait is generally visually assessed using blunt rating scales. Wearable sensors are suggested to bridge this gap for a more precise assessment of gait in daily clinic. The aim of this study was to use an inhouse developed sensor system (Striton), to assess gait in iNPH patients before and after tap-test and shunt surgery, with an emphasis on heel-height and step-width.

**Methods:** The Striton system was quick to attach and used to measure gait pattern during 25 m walking. Patients were assessed before and after tap-test (n = 13) or before and 3 months after shunt surgery (n = 10). Also, the gait of a group of healthy elderly (HE) (n = 83, age 70, 20 m walk) was measured. Preoperative gait parameters as well as change following tap-test/surgery were evaluated.

**Results:** Mean heel-height and -width in the HE was 16.7 ± 0.6 cm and 5.22 ± 0.89 cm. In iNPH, after tap-test, mean heel-height at push-off was increased from 13.9 to 15.1 cm (p = 0.016), but step-width was unchanged (6.5 to 6.2 cm). The HE was significantly different from the iNPH patients (p < 0.000 and p < 0.000 respectively). However, three months after surgery, heel-height as well as step-width were improved.

**Conclusions:** The measurements were easy to perform and generated objective results of parameters that are challenging to assess visually. This makes wearable sensors a promising tool for clinical assessment of gait.

## Objective assessment of NPH patients following ventriculoperitoneal shunt placement using activity monitoring data

### Desmond A. Brown^1^, Kenton R. Kaufman^2^, Jonathan Graff-Radford^3^, David T. Jones^3,4^, Jeremy K. Cutsforth-Gregory^3^, Petrice M. Cogswell^4^, and Benjamin D. Elder^2,5^

#### ^1^National Institute of Neurological Disorders and Stroke, United States National Institute of Health, Bethesda, Maryland, 20814, USA; ^2^Department of Biomedical Engineering, Mayo Clinic, Rochester, Minnesota, 55905, USA; ^3^Department of Neurology, Mayo Clinic, Rochester, Minnesota, 55905, USA; ^4^Department of Radiology, Mayo Clinic, Rochester, Minnesota, 55905, USA; ^5^Department of Neurosurgery, Mayo Clinic, Rochester, Minnesota, 55905, USA

##### **Correspondence:** Benjamin D. Elder (elder.benjamin@mayo.edu)

*Fluids and Barriers of the CNS* 2022, **19(1)**

**Introduction:** One goal of VP shunt placement in the treatment of idiopathic normal pressure hydrocephalus (iNPH) is to improve the patient’s gait. While various objective measures of gait are used in the clinic, these often provide a snapshot assessment, and there are limited data available on the real-world functional improvement following shunt placement.

**Methods:** Nineteen patients with iNPH were fitted with 4 activity monitors (hip, thigh, bilateral ankles), worn for 4 days preoperatively and 4 days postoperatively within 30 days of surgery. Continuous measurement of steps, cadence, body position, gait entropy, and the daily proportion spent “active” or “static” were obtained. The activity monitor data were compared to the standard gait assessments, modified Rankin Score (mRS), Incontinence Score, Montreal Cognitive Assessment (MoCA) Score, and Grooved Pegboard Tests.

**Results:** Postoperatively, there was significant improvement in mean entropy (0.60 to 0.80) and mean daily steps (2000 to 3000). There were no differences in mean cadence or percent of time spent active/static. There was no improvement noted on the mRS, MoCA, or Incontinence Score. Total steps were statistically significantly correlated with mean entropy (r = 0.742) and inversely correlated with mRS (r = − 0.501).

**Conclusions:** Daily activity monitors provided an early objective measure of improvement in mean gait entropy and total number of steps following shunt placement in iNPH patients. There was a 50% improvement in number of steps per day in the first 30 days postoperatively, with improved gait complexity, before any improvement in measures of cognitive function.

## Incidence and outcomes of patients with chronic secondary hydrocephalus undergoing VP shunting at a tertiary neurosurgery centre in the UK

### Sheikh M. B. Momin^1^, Samira Akmal^1^, Andrea J. Merchan^1^, Ahmed K. Toma^1^

#### ^1^Victor Horsley Department of Neurosurgery, The National Hospital for Neurology & Neurosurgery, London, WC1N 3BG

##### **Correspondence:** Sheikh Momin (sheikh@sheikhmomin.com)

*Fluids and Barriers of the CNS* 2022, **19(1)**

**Introduction:** Secondary hydrocephalus describes any CSF accumulation due to a concurrent central nervous system pathology, including subarachnoid haemorrhage (SAH) or tumour. There is little evidence on the incidence and outcomes of patients with chronic secondary hydrocephalus (defined as symptoms > 1 month in duration) undergoing a VP shunt.

**Methods:** We conducted a single-centre retrospective cohort study on all patients undergoing a VP shunt between 06/05/2016 to 30/03/2019 at the National Hospital for Neurology & Neurosurgery. Patients were included if they were over 18 and underwent shunts for chronic secondary hydrocephalus, whilst all other hydrocephalus patients (e.g. NPH) were excluded. Using our electronic health record, we collected data on patient demographics, indication, duration of symptoms, 30-day complications and patient survival to the present day, excluding duplicates.

**Results:** 65 patients (28 male, mean age 49) underwent 67 shunts (2 revisions) for secondary hydrocephalus during the study period. Of these, tumour was the most common indication (44.8%), followed by spinal (16.4%). 7 (10.4%) of the shunts inserted had a complication, of which 2 (28.6%) were within the first 30 days. Infection (4 patients) was the most common complication overall. Furthermore, 4 patients (6.0%) died during the follow-up period.

**Conclusions:** VP shunting for chronic secondary hydrocephalus mostly occurred for tumours at our centre. The complication rate is comparable to other studies in the literature. Given the diverse aetiology for secondary hydrocephalus, future studies may stratify analysis by aetiology, which may aid in developing predictive models for VP shunt treatment in chronic secondary hydrocephalus.

## Risk factors for post-traumatic hydrocephalus after decompressive craniectomy

### Sérgio M. F. Romualdo, T. Juratli, Gabriele Schackert, Kerim H. Sitoci-Ficici

#### Department of Neurosurgery, Universitätsklinikum Carl Gustav Carus Dresden, Germany

##### **Correspondence:** Sérgio Miguel Fernandes Romualdo (sergiomfromualdo@gmail.com)

*Fluids and Barriers of the CNS* 2022, **19(1)**

**Introduction:** Decompressive craniectomy (DC) is a main domain of traumatic brain injury (TBI) treatment. Little is known about the occurrence of post-traumatic hydrocephalus (PTH) in patients after DC.

**Methods:** We performed a retrospective study of TBI patients who underwent DC, dividing our study cohort into two groups, patients who did not develop a PTH (Non-PTH-group) and those who acquired a PTH (PTH-group). We evaluated several clinical and radiographic characteristics, including the type of TBI on the initial and follow-up CT-Scans, brain edema, ischemia, and volumetric measurements of the ventricle system. Moreover, we assessed the Rotterdam-, Helsinki-, and Stockholm-CT-scores in all patients.

**Results:** Our cohort comprised 126 patients (93 males and 33 females) with a median age of 53 years (18–84 years). In total, 34 patients (27%) developed a PTH. Clinical parameters with significant correlation with PTH development were a mRS higher than 3 points at the time of discharge after DC (PTH = 100% vs. Non-PTH = 78.3%; p = 0.002), and a shorter time interval from admission to DC (median time in PTH-group, 1.2 h vs. 1.7 h in the Non-PTH-group; p = 0.013). Radiographic parameters with a higher risk of developing a PTH included, a high Stockholm-CT-score (2–6 points) (PTH = 53% vs. Non-PTH = 28.3%; p = 0.038), and brain herniation through the DC defect (PTH = 82.4% vs. Non-PTH = 58.7%; p = 0.02). Further risk factors were post-traumatic ischemia (PTH = 70.6% vs. Non-PTH = 42.4%; p = 0.009), and hemorrhagic contusion’s progression (PTH = 47.1% vs. Non-PTH = 18.7%; p = 0.003).

**Conclusions:** Our study demonstrates that multiple factors are associated with PTH development, including a high Stockholm-CT-Score, brain herniation, post-traumatic ischemia, and hemorrhagic contusion progression.

## A benchtop simulation trainer for percutaneous shunt tap: model development and evaluation

### Jonathan P. Funnell^1,2^, Simon C. Williams^1,2^, John G. Hanrahan^1,2^, Zeid Abussuud^1^, Lewis Thorne^1^, Laurence D. Watkins^1^, Ahmed K Toma^1^

#### ^1^National Hospital for Neurology and Neurosurgery, London, UK; ^2^Wellcome / EPSRC Centre for Interventional and Surgical Sciences, London, UK

##### **Correspondence:** Jonathan P. Funnell (jonathan.funnell.13@ucl.ac.uk)

*Fluids and Barriers of the CNS* 2022, **19(1)**

**Introduction:** Percutaneous tap of a cerebrospinal fluid (CSF) reservoir can be a lifesaving procedure amongst patients with distal shunt catheter blockage. Despite this, availability of individuals trained in this procedure varies regionally, and no simulators are commercially available to assist in training.

**Methods:** A benchtop simulator model was developed by implantation of a fixed pressure shunt valve with integrated reservoir (Medtronic Delta Valve) into a simulated skin pad. The apparatus was filled with saline and volunteers working in a single neurosurgical unit were asked to perform percutaneous tap of the CSF reservoir. Participants completed a post-procedure questionnaire to evaluate Face and Content validity using 5-point Likert scales. Observers recorded time and number of passes required to complete the task to evaluate Construct validity.

**Results:** Thirteen participants (6 neurosurgery juniors, 7 neurosurgery registrars/senior fellows) completed the simulated shunt tap task. Participant-rated measures of Face validity revealed that the simulator looked and felt realistic (63/65 responses ‘Strongly Agree’/ ‘Agree’ to statements relating to Face validity). Participants particularly felt that the simulator was suitable and beneficial for teaching (65/65 responses ‘Strongly Agree’/ ‘Agree’ to statements relating to Content validity). Time and number of passes required to perform the procedure was a poor discriminator of prior experience (mean time: 18.9 s juniors, 15.7 s registrars/senior fellows, p = 0.634).

**Conclusions:** We describe the development and validation of a low-cost simulator to train healthcare professionals in percutaneous shunt tap. Participants rated the simulator highly in Face and Content validity; promising in the further development of this training simulator.

## Local guidelines for insertion of cerebrospinal fluid shunts reduce revision rate

### Kishan Karia^2^, Gopiga Thanabalasundaram^2^, Sebastian Yim^1^, Marian E. Byrne^2^, Georgios Tsermoulas^2,3^

#### ^1^University of Birmingham Medical School, United Kingdom; ^2^Department of Neurosurgery, Queen Elizabeth Hospital Birmingham, United Kingdom; ^3^Institute of Metabolism and Systems Research, University of Birmingham, United Kingdom

##### **Correspondence:** Georgios Tsermoulas (Georgios.Tsermoulas@uhb.nhs.uk)

*Fluids and Barriers of the CNS* 2022, **19(1)**

**Introduction:** Insertion of cerebrospinal fluid (CSF) shunts is a common neurosurgical operation. In an effort to reduce revision rate, guidelines on the management of patients that require CSF diversion procedure were developed and implemented in our Department. This study evaluates the impact of the guidelines on primary shunt revision rate.

**Methods:** A retrospective study was carried out between January 2018 and December 2020, in order to assess the change in the revision rate after the implementation of the CSF diversion guidelines in July 2019. The guidelines proposed: pathways for urgent and elective shunt operations; recommendations on peri-operative management, surgical techniques, insertion of external CSF drains and conversion to shunts; guidance in suspected malfunction; advice on clinical governance in relation to shunt surgery. We compared revision rates before and after the guidelines.

**Results:** There were 308 patients that underwent insertion of a primary shunt during the study period, 171 before and 137 after the introduction of the guidelines. The revision rate within 6 months of the implantation procedure was 17.2% for the whole cohort, 21.6% before and 11.2% after the guidelines. This represented a significant reduction of the revision rate of primary shunts (p = 0.021).

**Conclusions:** The introduction of local guidelines for insertion of CSF shunts significantly reduced primary shunt revision rates. We recommend development of robust surgical pathways and a reduction of variation in practice to improve outcomes for CSF shunting. Continuous monitoring of revision rates may identify modifiable factors and may further enhance shunt survival.

## Impact of transverse sinus dominance on intracranial pressure

### Sogha Khawari, Alaa Al-Mohammad, Matthew James Bancroft, Eleanor Moncur, Kanza Tariq, Peter Cowley, Laurence Watkins, Ahmed Toma

#### ^1^Victor Horsley Department of Neurosurgery, National Hospital for Neurology and Neurosurgery, London, UK

##### **Correspondence:** Sogha Khawari (sogha.khawari@nhs.net)

*Fluids and Barriers of the CNS* 2022, **19(1)**

**Introduction:** It is thought that the internal jugular veins (IJV) are the primary route for cranial venous outflow in supine position and the vertebral venous plexus when upright. Previous studies have noted a greater increase in intracranial pressure (ICP) when subjects turn their head in one direction. We hypothesised that in the supine position, head turning and consequently obstructing the IJV draining the dominant transverse sinus would lead to a greater rise in ICP compared to the non-dominant side.

**Methods:** A retrospective review of prospectively collected data in a single-centre study. Patients undergoing continuous ICP monitoring as part of their clinical management were recruited. ICP was measured in different head positions (neutral, turned right and left) when supine, seated and standing. Transverse sinus dominance was established by consultant radiologist report on venous imaging.

**Results:** Twenty patients were included in the study, with a median age of 44yrs and a mean weight of 95 kg. Venous system measurements revealed 85% right-sided vs 15% left-sided dominance. ICP rose more when turning head from neutral to dominant transverse sinus side (p = 0.007, CI 95% 0.96—5.63). The rise in ICP did not show a relationship with transverse sinus size. Additionally, transverse sinus dominance did not have a statistically significant relationship on ICP of head turning in standing and sitting position (p = 0.67 and p = 0.34 respectively).

**Conclusion:** This study confirms the importance of transverse sinus anatomy on ICP in the supine position. It may guide patient-specific advice and nursing care.

## Prognostic value of cerebrospinal fluid production rate in haemorrhagic stroke

### Kanza Tariq^1^, Mohamed A. Elborady^1^, Ahmed Toma^1^, Lewis Thorne^1^, Laurence Watkins^1^

#### ^1^National Hospital for Neurology and Neurosurgery, Queen Square, London, UK

##### **Correspondence:** Kanza Tariq (kanza.tariq@nhs.net)

*Fluids and Barriers of the CNS* 2022, **19(1)**

**Introduction:** Haemorrhagic stroke carries a 30-day mortality rate of 40%. Our study aimed to demonstrate cerebrospinal fluid production rate (PRcsf) as a potential prognostic tool for haemorrhagic stroke.

**Methods:** We performed a prospective observational study in all intracerebral haemorrhage (ICH) and subarachnoid haemorrhage (SAH) patients in our hospital who required external-ventricular drainage (EVD) of CSF as part of their treatment. The EVD was connected to LiquoGuard7 (Möller-Medical, Germany). The LiquoGuard7 was used to calculate PRcsf in the patients using the flow-rate data of the pump. Patients were followed up at 1 month post-haemorrhage and 6 months post-hospital discharge. Prognosis and patient outcome was recorded using the Glasgow Coma Scale (GCS) and Glasgow Outcome Scale (GOS).

Statistical analysis used SPSS (version 25.0, IBM) by independent t-test, comparing measured PRcsf to the observed GCS and GOS.

**Results:** 13 ICH and 21 SAH patients were studied. 7 ICH patients had PRcsf of 137 ml/hour ± 20(SD) and showed good prognosis. At 1 month post-bleed, these patients had GOS of 5 and GCS = 15 (p < 0.0001) which remained constant at 6 months follow-up. 6 ICH patients had ‘normal’ PRcsf (19 ml/hour ± 5SD) and showed poor prognosis, GCS = 3, GOS = 1 (p < 0.0001). 14 SAH patients had PRcsf 143 ml/hour ± 9(SD) and showed good prognosis. At 1 month post-bleed, these patients had GOS of 5 and GCS = 15 (p < 0.0001) which remained constant at 6 months follow-up. 7 SAH patients had ‘normal’ PRcsf (24 ml/hour ± 10SD) and showed poor prognosis, GCS = 3, GOS = 1 (p < 0.0001).

**Conclusion:** PRcsf may have a prognostic value in haemorrhagic stroke.

## Correlation between CSF NPTX2 and cognitive function, physiology, and biomarkers in idiopathic normal pressure hydrocephalus (iNPH) patients

### Megha Patel^1^, Yifan Zhang^2^, Paul Worley^1^, Abhay Moghekar^1^

#### ^1^Department of Neurology, Johns Hopkins University School of Medicine, Baltimore, MD, 21231, USA; ^2^Department of Biostatistics, Johns Hopkins University School of Medicine, Baltimore, MD, 21231, USA

##### **Correspondence:** Abhay Moghekar (am@jhmi.edu)

*Fluids and Barriers of the CNS* 2022, **19(1)**

**Introduction:** Neuronal Pentraxin 2 (NPTX2) is a synaptic protein responsible for regulating several processes both in the brain and in the periphery. While the role of NPTX2 as a novel synaptic biomarker in cognitive disorders is being elucidated, the effect of iNPH on synaptic integrity and function is not well understood.

**Methods:** iNPH patients (n = 361) referred for assessment to the Center for CSF disorders for shunt candidacy underwent a tap test (TT) during which CSF was banked. Demographic and clinical measures including age, cognitive function using the Montreal Cognitive Assessment (MoCA) test, gait performance using the Timed Up and Go (TUG) test, and Evan’s Index were ascertained. Abeta42/40 and phosphorylated-tau-181 (pTau-181) concentration were measured by chemiluminescent assays. CSF NPTX2 concentrations were determined using an ELISA. Spearman’s correlation was used to determine the correlation between CSF NPTX2 values and age, baseline MoCA score, baseline TUG score, post-shunt TUG score, Evan’s Index, abeta40/42 ratio, and pTau-181 concentration.

**Results:** There were 228 males and 133 females with a mean age of 77.5 ± 7.26 years. Evan’s index was 0.36 ± 0.05. MoCA was 21.4 ± 5.64. CSF NPTX2 levels in all iNPH patients referred for TT were 668.72 ± 1134.75 pg/ml. CSF NPTX2 levels in those selected for shunt surgery were 622.49 ± 1267.21 pg/ml. CSF NPTX2 concentrations did not show any correlations with age, MoCA (r = -0.012, p = 0.83 and r = 0.001, p = 0.87 respectively). CSF NPTX2 did not show correlations with abeta42/40 (r = -0.093, p = 0.076) but did show modest correlations with ptau181 (r = 0.44, p < 0.001).

**Conclusion:** CSF NPTX2 concentrations do not correlate with degree of ventriculomegaly, a screening cognitive measure commonly used in iNPH assessment or gait dysfunction. This reinforces the understanding that iNPH is a subcortical dementia and that synaptic degeneration is not an early feature of NPH pathophysiology.

## Time-dependent biomarker level change along with progression of CSF stagnation in idiopathic normal pressure hydrocephalus

### Kaito Kawamura^1,2^, Madoka Nakajima^2^, Masakazu Miyajima^3^, Chihiro Akiba^3^, Koichiro Sakamoto^2^, Chihiro Kamohara^2^, Hanbing Xu^2^, Ikuko Ogino^2^, Shinya Yamada^2,4^, Kostadin Karagiozov^2^, Akihide Kondo^2^

#### ^1^Department of Neurosurgery, Saiseikai Kawaguchi General Hospital, Saitama; ^2^Department of Neurosurgery, Juntendo University School of Medicine, Tokyo; ^3^Department of Neurosurgery, Juntendo Koto Geriatric Medical Center, Tokyo; ^4^Department of Neurosurgery, Kugayama Hospital, Tokyo

##### **Correspondence:** Kaito Kawamura (k-kawamu@juntendo.ac.jp)

*Fluids and Barriers of the CNS* 2022, **19(1)**

**Introduction:** We hypothesized that cerebrospinal fluid (CSF) stagnation in idiopathic normal pressure hydrocephalus (iNPH) will accelerate amyloid aggregation as was reported that CSF amyloid-beta oligomer (AβO^10−20^) accumulated in patients with iNPH, and decreased after CSF shunting. In this study, we assessed time-dependent CSF biomarker level and morphological changes along with the progression of CSF stagnation in cases of asymptomatic ventriculomegaly with features of iNPH (AVIM).

**Methods:** We retrospectively analyzed AVIM patients who later demonstrated iNPH and had received CSF shunting in Juntendo University (n = 34). We assessed time-dependent changes and correlations among AβO^10−20^ and morphological biomarkers, Evans Index (EI) as a parameter of ventricular expansion, and Z-EI and callosal angle (CA) as a parameter of disproportionately enlargement of subarachnoid space (SAS), in magnetic resonance images at AVIM diagnosis and treatment. We also assessed correlations of time-interval (TI) between AVIM and treatment, and AβO^10−20^ ratio(AβO^10−20^ at AVIM/AβO^10−20^ at treatment).

**Results:** The median [IQR] of TI was 17.5 [13.25–29.25] months. AβO^10−20^ was significantly increased at the point of treatment (p < 0.001). There was no correlation between TI and AβO^10−20^ ratio(p = 0.969). Z-EI was significantly increased, and CA was significantly decreased at the point of treatment while EI showed no significant change (p = 0.020, 0.040 and 0.173 respectively). The values of AβO^10−20^ significantly correlated with CA (p = 0.040).

**Conclusions:** We determined that Aβ aggregation, vertical expansion of lateral ventricles and disproportionate enlargement of SAS along with progression of CSF stagnation. Disproportionate enlargement of SAS was considered to correlate more with CSF stagnation than ventricular expansion.

## The effect of ventriculo-atrial shunt for the iNPH patients with high phosphorylated tau protein in cerebrospinal fluid

### Kiyoshi Takagi^1,2,3^, Shuichiro Asano^4^, Ryosuke Takagi^5^, Shuichiro Asano^3^, Shuichiro Asano^3^,

#### ^1^NPH Center, Abikoseijinkai Hospital, Abiko, 2700138, Japan; ^2^Institute of Industrial Science, The Tokyo University, Tokyo, 1530044, Japan; ^3^NPH Center, Jifukai Atsuchi Neurosurgical Hospital, Kagoshima, 8920842, Japan; ^4^Department of Neurosurgery, Kashiwatanaka Hospital, Kashiwa, 2770803, Japan; ^5^Department of Neurosurgery, Yokohama Minami Kyosai Hospital, Yokohama, 2360037, Japan

##### **Correspondence:** Kiyoshi Takagi (paulktkg@mac.com)

*Fluids and Barriers of the CNS* 2022, **19(1)**

**Introduction:** Idiopathic normal pressure hydrocephalus (iNPH) is one of the causes of treatable dementia and Alzheimer’s disease (AD) is one of the commonest comorbidity with regard to dementia. Phosphorylated tau protein (p-tau) in cerebrospinal fluid (CSF) is a biomarkers for AD. We retrospectively investigated whether the effects of the ventriculoatrial shunt (VAS) for iNPH were influenced by the preoperative CSF p-tau level.

**Methods:** We operated on 242 iNPH patients with pre-operative p-tau measurement at Kashiwatanaka hospital. MMSE and mRS were measured before, 6 m, and 1y after VAS. The patients were divided into 2 groups by the CSF p-tau values: L (< 30 pg/mL, n = 49) and H (≥ 30 pg/mL, n = 53). Data were shown in mean (SD). Statistically significant level was set p < 0.05.

**Results:** Mean ages were 76.6 (7.3) years for group L (n = 104) and 78.5 (5.8) years for group H (n = 138) (p = 0.039). Preoperative MMSE were 19.8 (7.7) and 18.9 (7.5) respectively (p = 0.352) and mRS were 2.8 (0.9) and 2.6 (0.8) respectively (p = 0.066). MMSE improved significantly at 6 m and 1y after VAS in group L (22.4 (7.9) and 22.3 (7.0) respectively). MMSE at 6 m and 1y after VAS in group H were 20.4 (7.9) and 20.2 (8.1) and only MMSE at 6 M was significantly better than preoperative MMSE. MRS at 6 m and 1y after VAS were significantly improved in both group (1.8 (1.2) and 1.7 (1.2) respectively in group L and 2.1 (1.0) and 2.0 (1.1) respectively in group H).

**Conclusions:** The results of this study suggest that VAS for the iNPH patients with AD is effective with respect to MMSE and mRS. We must not hesitate to give shunt surgery to the iNPH patients with AD.

## Experiences of performance and participation in daily activities in patients with idiopathic normal pressure hydrocephalus

### Katarina Owen^1^, Johanna Rydja^2^, Fredrik Lundin^3^, Johannes Österholm^4^

#### ^1^Department of Activity and Health, and Department of Health, Medicine and Caring Sciences, Linköping University, Linköping, Sweden; ^2^Department of Activity and Health, and Department of Biomedical and Clinical Sciences, Linköping University, Linköping, Sweden; ^3^Department of Neurology, and Department of Biomedical and Clinical Sciences, Linköping University, Linköping, Sweden; ^4^Unit of Occupational Therapy, Department of Health, Medicine and Caring Sciences, Linköping University, Linköping

##### **Correspondence:** Katarina Owen (katarina.owen@regionostergotland.se)

*Fluids and Barriers of the CNS* 2022, **19(1)**

**Introduction:** Previous research shows the effect of shunt surgery in patients with iNPH (idiopathic Normal Pressure Hydrocephalus) on performance and participation in daily activities and the burden for relatives. Few studies have addressed the experience of living with iNPH from an activity perspective. The aim of this study was to describe patients with iNPH experiences of performance and participation in daily activities.

**Methods:** Ten semi-structured interviews were conducted with patients diagnosed with iNPH. Recruitment and interviews took place before shunt surgery. Data were analyzed using qualitative content analysis.

**Results:** The patients described a need to adjust daily activities due to fear, uncertainty and lack of mental and physical energy. There was a need to abstain, not only from physical activities, but also from social—and calm activities. Changed social roles and the experience of decreased capacity resulted in a sense of an altered occupational identity, how the patients identified themselves based on the activities, they participated in.

**Conclusions:** This qualitative study, unusual in the field of iNPH, highlights the experience of performance and participation in daily activities in these patients. Gait impairment, cognitive decline and incontinence coexisting, may reinforce the experience and only a small impairment within each symptom may lead to severe consequences regarding occupational identity and participation in daily activities for the individual. The result suggests a need not only to focus on performance capacity in iNPH but also to address how each patient´s participation in daily activities is affected. This information is important when planning rehabilitation.

## The paced finger tapping assessment in idiopathic normal pressure hydrocephalus

### Yoko Shimizu^1,2^, Motoki Tanikawa^2^, Mitsuya Horiba^1^, Kento Sahashi^1^, Shoji Kawashima^3^, Akihiko Kandori^4^, Noriyuki Matsukawa^3^, Yoshino Ueki^1^, Mitsuhito Mase^2^

#### ^1^Department of Rehabilitation Medicine, Nagoya City University Graduate School of Medical Science, Nagoya, Japan; ^2^Department of Neurosurgery, Nagoya City University Graduate School of Medical Sciences, Nagoya, Japan; ^3^Department of Neurology and Neuroscience, Nagoya City University Graduate School of Medical Science, Nagoya, Japan; ^4^Hitachi, Ltd, Research and Development Group, Center for Exploratory Research, Tokyo, Japan

##### **Correspondence:** Yoko Shimizu (otyokos@med.nagoya-cu.ac.jp)

*Fluids and Barriers of the CNS* 2022, **19(1)**

**Introduction:** Several previous studies reported that the Finger Tapping(F-T) test is easily performed and useful for assessing motor function of upper limbs in patients with Idiopathic normal pressure hydrocephalus (iNPH). However, quantitative evaluation of F-T test for patients with iNPH has not been established. The purpose of this study is to investigate the usefulness of the F-T test with paced stimuli as motor evaluation and screening test for iNPH.

**Methods:** Sixteen age-matched healthy controls (mean age 73 ± 5 years;7/16 male) and fifteen participants with a diagnosis of definitive iNPH (mean age 76 ± 5 years; 8/15 male) completed the study(mean ± SD). F-T performance of the index finger and thumb was quantified using magnetic sensing device. The participants performed repetitive tapping for 15 s with each hand, following to the pace at the rate of 2.0 Hz. We evaluated F-T parameters including the mean of maximum amplitude, and closing velocity so on. We defined the mean of Maximum amplitude as *M-Amplitude* and the maximum of closing movement velocity as *cl-Velocity*.

**Results:** The M-Amplitude of iNPH patients (54.2 ± 27.5 mm) is significantly smaller than that of controls (84.2 ± 14.1 mm) on dominant hand (P < 0.01). The cl-Velocity of iNPH patients (1.1 ± 0.4 m/s) is significantly lower than that of controls (1.8 ± 0.4 m/s) on dominant hand (P < 0.01). We confirmed the improvement in F-T test parameters after shunt surgery.

**Conclusions:** The F-T test can be an alternative or supplementally method for screening and assessment of motor function in patients with iNPH.

## Prevalence of fecal incontinence in Normal pressure hydrocephalus – a prospective evaluation of 100 patients

### Uwe Kehler, Sven Petersen

#### Department of Neurosugery, Asklepios Klinik Altona, Hamburg, Germany.=

##### **Correspondence:** Uwe Kehler (uwekehler@hotmail.com)

*Fluids and Barriers of the CNS* 2022, **19(1)**

**Introduction:** Bladder dysfunction is one of the main symptoms of NPH beside gait disturbance and cognitive decline forming the Hakim Triad. Fecal urgency and incontinence is often described as an additional symptom, however, no exact numbers are known. The aim of this study was to investigate the prevalence of fecal urgency and incontinence in NPH patients.

**Methods and patients:** Patients who presented to our department with confirmed diagnosis of NPH between January and December 2021 were interviewed prospectively about fecal function. Additionally, the extent of gait disturbance, cognitive decline, ventriculomegaly (Evans-Index), DESH presence, age, gender and length of history were documented. In those who were operated upon the postoperative development of stool incontinence was documented.

**Results:** 100 patients were evaluated (67 men, 33 females, medium age 77.5 years, medium Evans Index: 0,37, 87 with positive DESH pattern). 97 patients showed gait disturbance, 84 a cognitive decline and 87 bladder dysfunctions. 78 patients showed the complete Hakim triad. 32 patients complained about fecal incontinence (20 with urge incontinence, 12 with complete incontinence). 29 of these patients were shunted, 17 (57%) recovered completely, 9 (31%) partially, and 3 (10%) did not show any change.

**Conclusion:** Fecal urgency and incontinence is a frequent finding in NPH (32%) and is essential for the quality of life. In the general population, fecal incontinence in the elderly is found in up to 15%. The more than twofold higher prevalence in NPH patients and the high percentage of post-shunted improvement suggests that NPH often directly causes fecal disturbance.

## Bowel and bladder symptoms in idiopathic normal pressure hydrocephalus

### Andreas Eleftheriou^1^, Susanna Walter^2^, Johanna Rydja^3^, Katarina Owen^4^, Rafael Holmgren^5^, Fredrik Lundin^1^

#### ^1^Department of Neurology, and Department of Biomedical and Clinical Sciences, Linköping University, Linköping, Sweden; ^2^Department of Biomedical and Clinical Sciences (BKV), Division of Inflammation and Infection, Linkoping University, Linkoping, Sweden; ^3^Department of Activity and Health, and Department of Biomedical and Clinical Sciences, Linköping University, Linköping, Sweden; ^4^Department of Activity and Health, and Department of Health, Medicine and Caring Sciences, Linköping University, Linköping, Sweden; ^4^Department of Neurosurgery and Department of Biomedical and Clinical Sciences, Linköping University, Linköping, Sweden

##### **Correspondence:** Andreas Eleftheriou (andelef2002@yahoo.gr)

*Fluids and Barriers of the CNS* 2022, **19(1)**

**Introduction:** Although urinary incontinence is one of the key symptoms in idiopathic Normal Pressure Hydrocephalus (iNPH) it has been poorly investigated. Bowel symptoms reported by patients has not yet been studied. The aim of this study is to investigate bladder and bowel symptom before and after the shunt surgery.

**Methods:** Forty-seven iNPH-patients, (27 m/ 20f), median age 77 (62–84) years have yet been included in this ongoing prospective single-centre study. The patients underwent a pre- and 3-months post-operative clinical work-up including Hellstrom´s iNPH-scale to characterize their clinical profile. Wexner Incontinence grading score, the International Consultation on Incontinence Questionnaire-Urinary Short Form (ICIQ-UI SF) and a Bowel symptom Questionnaire were used to study any bladder and/or bowel symptoms before and after surgery.

**Results:** The median disease duration was 24 months (6–120). Thirty-eight patients had gait/balance disturbance as first symptom whereas five had cognitive decline, and four bladder symptoms. The total Hellstrom’s iNPH scale score was significantly improved (p = 0.03) postoperatively (n = 20). Preoperatively, ICIQ-UI > 3 in 83% of patients indicating urinary incontinence. Faecal incontinence (FI) according to Wexner score was reported in 82%, with a median score of FI of 7 (range 3–10), indicating mild to moderate FI. Half of the patients had accidental leakage of intestinal gas at least once in a week.

**Conclusion:** It seems that both bladder and bowel symptoms are common in patients with iNPH. As these are preliminary results further analysis must be done to evaluate if the symptoms improve after a shunt operation.

## Urinary outcome after shunt placement in patients with Normal Pressure Hydrocephalus and urinary comorbidities: a single center experience

### Fernando Hakim^1,2^, Juan F. Ramon^1,2^, Diego F. Gómez^1,2^, Juan A. Mejia^1,2^, Andrés D. Ramírez^1,3^, Salvador M. Mattar^1,2^

#### ^1^Department of Neurosurgery, Fundación Santa Fe de Bogotá, Bogotá, Colombia; ^2^Normal Pressure Hydrocephalus Center of Excellence, Bogotá, Colombia; ^3^Universidad de los Andes, Bogotá, Colombia

##### **Correspondence:** Fernando Hakim-Daccach (fhakimd@gmail.com)

*Fluids and Barriers of the CNS* 2022, **19(1)**

**Introduction:** Urinary incontinence is one of the symptoms of NPH triad and is present in up to 80% of NPH cases. NPH is known for its reversible symptoms, nevertheless, it is widely under treated. In some cases the patients with NPH do not undergo surgery due to the notion that the patients´ comorbidities limit their range of improvement. This paper aims to describe the urinary outcome after shunt placement and its relation to other urinary diagnosis.

**Methods:** We made a retrospective cohort-study. Patients with NPH diagnosis with urinary incontinence, who underwent shunt placement surgery during 2016—2022 were included (n = 35). The population was divided into groups with and without other urinary diagnosis which may cause incontinence. We analyze and compare their ICIQ-UI SF score before and after shunt placement.

**Results:** 34.2% (n = 12) of patients had a prior urinary diagnosis. In general 68% of patients improved in at least 20% their initial ICIQ-UI SF score, and 22% of patients were symptom free. In the group with urinary comorbidities, 58.3% of patients improved by at least 20% of their initial ICIQ-UI SF score. In the group without prior urinary comorbidities 73.8% of patients improved in at least 20% their initial ICIQ-UI SF score.

**Conclusions:** Both groups displayed encouraging results in their one month postoperative evaluations. In our center´s experience patients with NPH, with or without urinary comorbidities have an important chance of partial to complete recovery of urinary incontinence.

## Implementing advanced psychometrics to identify significant change between cognitive test scores in response to CSF drainage

### Aishah Hannan^1^, Sharon Lau^1^, Emma King^1^, Abigail Beard^1^, Tina O’Farrell^2^, Elizabeth Cray^2^, Samuel Jeffery^2^, Samiul Muquit^2^, Thomas Davis^1^, Rupert Noad^1^

#### ^1^Department of Neuropsychology, University Hospital Plymouth NHS Trust, United Kingdom; ^2^Department of Neurosurgery, University Hospital Plymouth NHS Trust, United Kingdom

##### **Correspondence:** Aishah Hannan (aishah.hannan@nhs.net)

*Fluids and Barriers of the CNS* 2022, **19(1)**

**Introduction:** Change in cognition is a clinical hallmark of Idiopathic Normal Pressure Hydrocephalus (iNPH). Accurate measurement of outcomes is crucial to optimizing intervention during cerebrospinal fluid (CSF) drainage. However, repeated administration of cognitive tests, decreases measurement reliability. Simple change scores (SCS) do not account for test–retest error. The present study uses reliable change indices (RCI) to improve reliability and validity in cognitive measurement.

**Methods:** A retrospective record review of 15 patients who underwent CSF drainage for suspected iNPH between October 2021 to April 2022 was conducted. Pre-post CSF drainage data for six cognitive tests was extracted: Trail Making Task A and B, RBANS Coding, Semantic Fluency, Immediate and Delayed Story Recall. SCS and RCI scores were computed using Classical Test Theory (Duff, 2012), and categorized into overall improvement, deterioration, or no change.

**Results:** Fifteen patients (Female *n* = 10; Male *n* = 5; age *M* = 75 years), underwent successful CSF drainage. Change analysis using SCS categorised most patients as improved (*n* = 7), rather than deteriorated (*n* = 4) or no change (*n* = 4). Change analysis using RCI categorized most patients as no change (*n* = 12), rather than deteriorated (*n* = 2) or improved (*n* = 1).

**Conclusions:** Using simple difference in raw scores to measure change in cognition, introduces risk of type 1 error (false positives). The RCI equation is proposed as a method to improve reliability in cognitive outcome measurements following CSF drainage. Development of tests that are less susceptible to test–retest effects is encouraged.

## Diffusion tensor image analysis along the perivascular space (DTI-ALPS) reflects impaired activity of the glymphatic system in iNPH

### Charalampos Georgiopoulos^1,2^, Anders Tisell^2,3^, Rafael Holmgren Turczynski^4^, Andreas Eleftheriou^5^, Johanna Rydja^5^, Fredrik Lundin^5^, Lovisa Tobieson^4^

#### ^1^Department of Radiology and Department of Health, Medicine and Caring Sciences, Linköping University, Sweden; ^2^Center for Medical Image Science and Visualization (CMIV), Linköping University, Sweden; ^3^Department of Medical Radiation Physics and Department of Health, Medicine and Caring Sciences, Linköping University, Sweden; ^4^Department of Neurosurgery in Linköping, and Department of Biomedical and Clinical Sciences, Linköping University, Sweden; ^5^Department of Neurology, and Department of Biomedical and Clinical Sciences, Linköping University, Sweden

##### **Correspondence:** Charalampos Georgiopoulos (Charalampos.Georgiopoulos@liu.se)

*Fluids and Barriers of the CNS* 2022, **19(1)**

**Introduction:** The activity of the glymphatic system can be non-invasively measured with diffusion tensor image analysis along the perivascular space (DTI-ALPS). The aim of this study was to compare ALPS-index in idiopathic normal pressure hydrocephalus (iNPH) patients and healthy controls and evaluate potential correlation with clinical symptoms.

**Methods:** Twenty-six consecutive iNPH patients and twenty healthy age-matched controls underwent diffusion tensor imaging with MRI. The diffusivities in the x, y, and z axes along the projection and association fibers were calculated, to acquire the ALPS-index in each hemisphere. iNPH patients underwent clinical examination with the Hellstrom iNPH scale, Timed Up and Go (TUG), and Mini Mental State Examination (MMSE).

**Results:** There was no difference in age [mean age 75.7 (± 5.6) vs. 73 (± 5.7) years, respectively p = 0.107] or sex [10 (38%) vs. 8 (40%) males, respectively p = 0.577] between groups. iNPH patients demonstrated significantly lower ALPS-index compared to healthy controls, in the right hemisphere [median 1.08 (IQR 0.96–1.2) and 1.47 (1.33–1.64), p < 0.001] and the left hemisphere [1.06 (0.9–1.16) and 1.46 (1.29–1.7) p < 0.001]. ALPS-index was negatively correlated with the neuropsychology domain of the Hellstrom iNPH scale both in the right hemisphere (rho = − 0.465, p = 0.019) and the left hemisphere (rho = − 0.457, p = 0.022). There was no significant correlation between ALPS-index and TUG, MMSE or the other domains of the Hellstrom iNPH scale.

**Conclusions:** DTI-ALPS-index is lower in iNPH patients compared to healthy controls which may indicate impaired activity of the glymphatic system.

## Importance of oscillating flow of cerebrospinal fluid in idiopathic normal pressure hydrocephalus

### Shigeki Yamada^1,2,3^, Masatsune Ishikawa^3,4^, Hirotaka Itou^5^, Shinnosuke Hiratsuka^6^, Yoshiyuki Watanabe^6^, Satoshi Ii^7^, Tomohito Otani^8^, Shigeo Wada^8^, Marie Oshima^2^, Kazuhiko Nozaki^1^

#### ^1^Department of Neurosurgery, Shiga University of Medical Science, Shiga, Japan; ^2^Interfaculty Initiative in Information Studies / Institute of Industrial Science, The University of Tokyo, Tokyo, Japan; ^3^Normal-Pressure Hydrocephalus Center, Rakuwakai Otowa Hospital, Kyoto, Japan; ^4^Rakuwa Villa Ilios, Kyoto, Japan; ^5^Medical System Research & Development Center, FUJIFILM Corporation, Tokyo, Japan; ^6^Department of Radiology, Shiga University of Medical Science, Shiga, Japan; ^7^Faculty of System Design, Tokyo Metropolitan University, Tokyo, Japan; ^8^Department of Mechanical Science and Bioengineering, Graduate School of Engineering Science, Osaka University, Osaka, Japan

##### **Correspondence:** Shigeki Yamada (shigekiyamada39@gmail.com)

*Fluids and Barriers of the CNS* 2022, **19(1)**

**Introduction:** To assess the effect of CSF dynamics in iNPH, we measured the stroke volume (SV) and oscillatory shear stress (OSS) in the ventricles on 4D Flow MRI and complex small oscillating flow of CSF in the ventricles and subarachnoid spaces on intravoxel incoherent motion (IVIM) MRI.

**Methods:** 3D T2-weighted MRI, 4D Flow MRI, IVIM MRI were performed using a 3-T MRI in 75 patients with iNPH and 136 healthy volunteers aged ≥ 20 years. The 3D flow vectors and OSS in each ROI were measured using the 4D Flow application, and IVIM analysis was performed using the IVIM application on SYNAPSE 3D workstation (FUJIFILM Corporation).

**Results:** The normal reciprocating CSF movement was the largest at the foramen magnum, and was almost impeded at the foramen magnum. In iNPH patients, the foramen magnum was enlarged and oscillating CSF flow in the ventricular systems were increased. In addition, the high OSS in the cerebral aqueduct was observed iNPH. However, the mean values of fraction factor (f, %) on IVIM MRI in the whole lateral ventricles and anterior part of the third ventricle, and almost all parts of the subarachnoid spaces except the prepontine cistern and lower part of the interhemispheric fissure were significantly lower in iNPH.

**Conclusions:** This study performed the first quantitative evaluation of small and large oscillating CSF flow and OSS in the ventricles and subarachnoid spaces in iNPH and healthy volunteers by using 4D flow and IVIM MRI.

## CSF flows dynamics versus morphological and pressure-related parameters considered in NPH patients

### Olivier Balédent^1,2^, Kimi Owashi^1^, Serge Metanbou^1,3^, Vakaramoko Kone^1^, Zofia Czosnyka^4^, Marek Czosnyka^4^, Peter Smielewski^4^, Cyrille Capel^1,5^

#### ^1^Chimère UR 7516, Jules Verne University, Amiens, France; ^2^Image processing, Jules Verne University hospital, Amiens, France; ^3^Radiology, Jules Verne University hospital, Amiens, France; ^4^Department of Clinical Neurosciences, University of Cambridge, Cambridge, UK; ^5^Neurosurgery, Jules Verne University hospital, Amiens, France

##### **Correspondence:** Olivier Balédent (olivier.baledent@chu-amiens.fr)

*Fluids and Barriers of the CNS* 2022, **19(1)**

**Introduction:** Cerebrospinal fluid (CSF) stroke volume measured through the aqueduct of Sylvius (**SV**_**aq**_) by phase-contrast MRI (PC-MRI) is one of the most discussed image-based biomarkers proposed as a possible indicator of positive surgical outcome in normal pressure hydrocephalus (NPH) patients. Alternatively, CSF stroke volume through the spinal compartment (**SV**_**c2c3**_) can also be measured by PC-MRI. This study combines clinical, morphological, and pressure-related parameters considered in NPH patients’ management to evaluate potential relationship between these parameters and the **Sv**_**aq**_ and the **SV**_**c2c3.**_

**Methods:** 28 patients (74 ± 9 years) with suspected NPH underwent clinical examination, PC-MRI and infusion test. We measured by PC-MRI, CSF flows dynamics in the cerebral aqueduct and in the spinal canal at C2-C3 level. Then **SV**_**aq**_ and **SV**_**c2c3**_ were calculated**.** Moreover, physicians also estimated other different features: Evans’ index, callosal angle, DESH score, iNPH score, Resistance to CSF outflow (R_out_).

**Results: Sv**_**aq**_ showed a poor correlation with the different features evaluated, however, **SV**_**c2c3**_ presented statistically significant correlation with R_out_ (R^2^ = 0.33, p = 0.0028).

**Conclusions:** Although **SV**_**aq**_ has been proposed to predict surgical outcome of patients with NPH, it presented a poor correlation with complementary markers commonly evaluated for diagnosis. More interestingly, these preliminary results showed that **SV**_**c2c3**_ has a significant correlation with R_out_, a complementary parameter used to predict shunt responsiveness in NPH patients. Indeed, **SV**_**c2c3**_ represent the CSF volume flush quickly ejected from the intracranial compartment as a compensatory response to vascular brain expansion, which could explain the relationship with the resistance to CSF outflow.

**Study supported by**: Revert Project, Interreg, France (Channel Manche) England, funded by European Regional Development Fund.

## Automated quantification of lateral ventricle volumes in normal pressure hydrocephalus from computed tomography scans using deep learning

### Meera Srikrishna^1,2^, Woosung Seo^3^, Johan Virhammar^4^, David Fällmar^3^, Michael Schöll^1,2,5,6^

#### ^1^Wallenberg Centre for Molecular and Translational Medicine, University of Gothenburg, Gothenburg, 40530, Sweden; ^2^Department of Psychiatry and Neurochemistry, Institute of Physiology and Neuroscience, University of Gothenburg, Gothenburg, 40530, Sweden; ^3^Department of Surgical Sciences, Neuroradiology, Uppsala University, Uppsala, 75185, Sweden; ^4^Department of Medical Sciences, Neurology, Uppsala University, Uppsala, 75185, Sweden; ^5^Dementia Research Centre, Institute of Neurology, University College London, London, WC1N 3BG, UK; ^6^Department of Clinical Physiology, Sahlgrenska University Hospital, Gothenburg, 40530, Sweden.

##### **Correspondence:** Michael Schöll (michael.scholl@neuro.gu.se)

*Fluids and Barriers of the CNS* 2022, **19(1)**

**Introduction:** Brain computed tomography (CT) is an affordable and widely available modality. In idiopathic normal pressure hydrocephalus (iNPH), CT scans are used to assess ventricular enlargement and other morphological features. We aim to quantify the lateral ventricle volumes before and after shunt surgery in iNPH using automated CT-based volumetry, derived with a novel deep learning approach.

**Methods:** We developed U-Net-based deep learning models to segment ventricular cerebrospinal fluid (VCSF) from CT images. We initialized the U-Net with transferable features from a pre-trained model trained to detect VCSF-related morphological features from magnetic resonance imaging-based VCSF labels. The U-Net was then trained to identify VCSF in CT images learning from manual segmentations. The training set comprised of 62 iNPH datasets and paired manual labels from Uppsala University Hospital, of which 23 patients had post-shunt scans with intraventricular catheters. Post model development, we evaluated the segmentation performance of deep-learning-derived VCSF against manual-VCSF as standard criterion.

**Results:** In the iNPH training dataset (*n* = 62), high volumetric correlations of *r* = 0.94 were observed between automatically and manually derived VCSF. The volumetric correlations between automatically and manually derived VCSF in pre- (*r* = 0.98) and post-shunt images (*r* = 0.97) were similar.

**Conclusions:** Preliminary results demonstrate strong potential for automated CT-derived VCSF volumetry in the assessment of iNPH, with comparable performance to manual segmentations. The model performed surprisingly well on scans with an intraventricular catheter. Future work will explore the model performance in a validation cohort, analyze volumetric changes after shunting, and explore additional aspects of diagnostic potential of automated CT-volumetry.

## Deep-learning cortical thickness analysis as predictor for shunt surgery effectiveness in possible iNPH patients: a preliminary study

### Daniele Piccolo^1,2^, Sara Fabbro^1,2^, Daniele Bagatto^3^, Maria Cristina De Colle^3^, Enrico Belgrado^4^, Miran Skrap^2^, Francesco Tuniz^2^

#### ^1^Department of Neuroscience, University of Padua, Padua, Italy; ^2^Department of Neurosurgery, ASUFC Santa Maria della Misericordia, Udine, Italy; ^3^Department of Neuroradiology, ASUFC Santa Maria della Misericordia, Udine, Italy; ^4^Department of Neurology, ASUFC Santa Maria della Misericordia, Udine, Italy

##### **Correspondence:** Daniele Piccolo (ing.daniele@gmail.com)

*Fluids and Barriers of the CNS* 2022, **19(1)**

**Introduction:** While optimal treatment for iNPH is quite known, diagnosis remains a topic of debate, with different procedures suggested as potential predictors for shunt surgery effectiveness. Various tests, biomarkers, and neuroimaging techniques can be used; however, the diagnostic accuracy is below optimal when performed with neuroimaging techniques alone and not always cost-effective when achieved with CSF biomarkers. Quantitative computational methods for cortical thickness analysis have become available in the last decade, and they only require regularly available T1-weighted brain MR images.

**Methods:** We analyzed 226 total patients referred to our clinic from January 2015 until December 2020. After exclusion criteria, the final sample consisted of 64 possible iNPH patients: 42 were diagnosed as probable iNPH and underwent VPS surgery, while 22 did not receive a surgical indication after diagnostic procedures. We investigated differences in cortical thickness for all possible iNPH patients using a public deep-learning-based neuroimaging pipeline.

**Results:** In 25 total patients with a negative CSFTT or not responsive to shunt surgery, a significant localized cortical thinning was seen in the lateral surface of the frontal and parietal lobes (bilateral superior and middle frontal gyrus, bilateral pars opercularis, bilateral superior parietal gyrus, bilateral supramarginal gyrus, left inferior parietal gyrus, bilateral precuneus).

**Conclusions:** Preoperative cortical thickness is a feasible analysis that might be useful in defining the optimal therapeutic plan for possible iNPH patients. A large multicentric study is encouraged to elucidate specific patterns and ratios of cortical thickness that might be used as potential predictors for shunt surgery effectiveness.

## Association of aqueductal flow and desh features in a population-based study

### Petrice M. Cogswell^1^, Jeffrey L. Gunter^1^, Stephen D. Weigand^2^, Benjamin D. Elder^3^, Hugo Botha^4^, Jeremy K. Cutsforth-Gregory^4^, David T. Jones^1,4^, David S. Knopman^4^, Prashanthi Vemuri^1^, Ronald C. Petersen^2,4^, Clifford R. Jack Jr^1^, Jonathan Graff-Radford^4^

#### ^1^Department of Radiology, Mayo Clinic, Rochester, MN, 55905, USA; ^2^Department of Quantitative Health Sciences, Mayo Clinic, Rochester, MN, 55905, USA; ^3^Department of Neurologic Surgery, Mayo Clinic, Rochester, MN, 55905, USA; ^4^Department of Neurology, Mayo Clinic, Rochester, MN, 55905, USA

##### **Correspondence:** Petrice M. Cogswell (Cogswell.petrice@mayo.edu)

*Fluids and Barriers of the CNS* 2022, **19(1)**

**Introduction:** Disproportionately enlarged subarachnoid space hydrocephalus (DESH) is an indirect marker of disordered CSF dynamics, highly associated with NPH and found in 6–7% of the aging population. A more direct assessment of CSF dynamics may be achieved with measurement of CSF flow, and elevated flow through the cerebral aqueduct has been found in some patients with NPH. The goal of this study was to assess if aqueductal flow is associated with the DESH score in a population-based sample.

**Methods:** The study included 447 participants in the Mayo Clinic Study of Aging, 384 cognitively unimpaired and 61 with mild cognitive impairment or dementia, median (IQR) age 73 (64,81). The DESH score was determined via a support vector machine algorithm trained to identify DESH features on T1-weighted MRI. Phase contrast MRI was performed at the cerebral aqueduct using a standardized protocol.

**Results:** Average total flow increased with age, spearman rho = 0.27(0.19,0.35), ventricular volume, rho 0.40(0.32,0.47) and DESH score, rho = 0.18(0.09,0.26). In a linear regression model, average total flow increased significantly with a 10-year older age (11% ± 2%), male sex (13% ± 5%), and a one-unit higher DESH score (5% ± 2%).

**Conclusions:** Weak correlation of aqueductal flow with DESH score in this population-based sample may reflect a similar phenomenon as the variably elevated flow seen in NPH patients. To better understand mechanistic relationships of flow and morphologic features of NPH, future work will assess associations of individual DESH features with aqueductal flow in the population and clinical NPH patients.

## Vision and health-related quality of life in adolescents with surgically treated hydrocephalus in infancy

### Rezhna Taha Najim^1^, Marita Andersson Grönlund^1,2^, Susann Andersson^1,3^

#### ^1^Department of Clinical Neuroscience, Institute of Neuroscience and Physiology, Sahlgrenska Academy, University of Gothenburg, Gothenburg, Sweden; ^2^Deparment of Ophthalmology, Faculty of Medicine and Health, Örebro University, Örebro, Sweden; ^3^Region Västra Götaland, Sahlgrenska University Hospital, Department of Ophthalmology, Mölndal, Sweden

##### **Correspondence:** Susann Andersson (susann.andersson@gu.se)

*Fluids and Barriers of the CNS* 2022, **19(1)**

**Introduction:** Hydrocephalus (HC) is a medical condition that has a significant impact on children and their caregivers. The objective of this study was to evaluate visual acuity (VA) and health-related quality of life (HR-QoL) in relation to aetiology and additional diagnoses in a population-based group of individuals with HC surgically treated in infancy. The results were compared with a healthy age-and sex matched control group.

**Methods:** 23/26 adolescents (15 male; mean age 15.0 years) with HC and 31 controls (18 male; mean age 15.4 years) were able to participate. VA was tested with a vision chart. HR-QoL was measured with the Pediatric Quality of Life Inventory (PedsQL), consisting of physical and psychosocial self-report and parent proxy-report.

**Results:** Adolescents with HC and their parents showed lower total HR-QoL score (80.1 and 59.5) compared with controls (92.4 and 93.5); p = 0.0019 and p ≤ 0.0001. Parent-reported total HR-QoL scores were significantly lower compared with teen-reported (p = 0.010). The median VA was 1.0 decimal (range 0.2–1.25) in the HC-group. There were no significant differences between VA < 0.5 (n = 4) or VA ≥ 0.5 (n = 19) and HR-QoL, although parents tended to score lower HR-QoL in both psychosocial and physical health. Teenagers with myelomeningocele (n = 10) showed significantly lower physical health score (p = 0.0017) compared with other aetiologies. No significant differences in HR-QoL were found regarding prematurity, epilepsy, learning disabilities, or shunt complications.

**Conclusions:** In the present study, adolescents with HC and their parents showed worse HR-QoL compared with healthy controls, however teenagers with HC rate their health better than their parents do.

